# Dissipatively Fueled Unidirectionally Communicating DNA Circuits That Control Biocatalysis

**DOI:** 10.1002/anie.202523416

**Published:** 2026-03-12

**Authors:** Philippe Jung, Daniel Felder, Gurudas Chakraborty, Tim Seifert, Matthias Wessling, Lifei Zheng, Andreas Herrmann

**Affiliations:** ^1^ Institute of Technical and Macromolecular Chemistry RWTH Aachen University Aachen Germany; ^2^ DWI‐Leibniz Institute for Interactive Materials Aachen Germany; ^3^ AVT.CVT – Chair for Chemical Process Engineering RWTH Aachen University Aachen Germany; ^4^ Wenzhou Institute University of Chinese Academy of Sciences Wenzhou P.R. China

**Keywords:** biocatalysis, dissipative self‐assembly, DNA networks, unidirectional communication

## Abstract

Unlike most synthetic systems, life constantly reorganizes itself through the irreversible consumption of energy‐rich molecules and exhibits dynamic functionalities governed by spatiotemporally controlled biocatalytic processes. Inspired by this, we herein demonstrate unidirectionally communicating, out‐of‐equilibrium DNA circuits that enable network‐guided control of the biocatalytic activity of an enzyme. The unidirectional communication is realized through the programmed, dissipative manipulation of information transfer. In this process, transient activation of a DNAzyme generates the fuel required for the temporal activation of trypsin. Prior to establishing this information‐transfer framework, we employed fuel‐driven dissipation to autonomously and temporally regulate the activity of nucleic acid and protein‐based enzymes, each operating in individual cycles. The transient state of the systems is attained through rapid hybridization of DNA strands, while digestion of the DNA fuel by exonucleases regenerates the initial equilibrium state. These processes proceed in a cyclic manner, allowing the systems to attain an out‐of‐equilibrium state. Precise control over the lifetime of this transient state was achieved by regulating external factors, such as DNA fuel and exonuclease concentrations, and internally by exploiting the toe‐hold length‐dependent digestion kinetics of the exonucleases. To establish our findings, we adopted a combined approach that includes both experimental and computational methodologies.

## Graphical abstract

Unidirectionally communicating, out‐of‐equilibrium DNA circuits are used to control enzyme activity. Transient DNAzyme activation generates fuel for temporal trypsin activation. Cyclic fuel‐driven hybridization and exonuclease‐mediated reset enable precise control over lifetimes, which is analyzed using combined experimental and computational approaches.

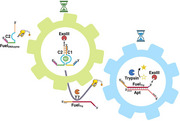

## 1 Introduction

Contrary to synthetic materials and structures, living systems continuously reorganize themselves by consuming energy‐rich molecular fuels, leading to autonomous, active, and adaptive behaviors. These dynamic functionalities stem from the spatiotemporally controlled, out‐of‐equilibrium operation of biomolecular networks, encompassing processes like GTP‐driven tubulin assembly into microtubules [1, 2], ATP‐fueled actin filament crosslinking [3, 4], and mitotic spindle formation [5]. Collectively, such processes govern crucial cellular functions, including cell adhesion [6], morphology [7], motility [8], and division [9]. Similarly, information processing in cells occurs through energy‐consuming processes. This can be exemplified by the central dogma, where DNA is transcribed into RNA and subsequently translated into proteins [10]. Beyond this linear flow of information, small RNAs introduce additional regulatory layers: microRNAs (miRNAs) mediate the downregulation of target messenger RNAs (mRNAs), thereby reducing protein output [11], while small interfering RNAs (siRNAs), incorporated into RNA‐induced silencing complexes (RISC), trigger endonucleolytic cleavage of complementary mRNAs, resulting in decreased protein levels [12]. These cascade processes demonstrate how nucleic acid‐encoded information can dynamically and actively regulate protein concentrations in a tightly controlled manner.

Inspired by these biological processes, researchers have designed dissipative circuits and functions involving nucleic acid building blocks due to several unique features of this class of materials: First, DNA and RNA undergo predictive self‐assembly due to base‐pairing and availability of various secondary structures, which include aptamers [13], deoxyribozymes (DNAzymes) [14, 15, 16], G‐quadruplexes [17, 18], i‐motifs [17, 19], and hairpins [20]. This led to a multitude of static DNA‐based self‐assembled nanostructures and materials, including DNA origami [21, 22], liquid crystals [23, 24, 25, 26, 27], bulk films [28, 29, 30], hydrogels [31, 32, 33, 34], nanotubes [35, 36], micelles made of DNA amphiphiles [37, 38, 39, 40], and DNA‐guided nanoparticle assemblies [41]. Second, defined nucleic acid sequences of different lengths are available by chemical synthesis and biosynthetic strategies or combinations of those, including solid‐phase synthesis [42, 43, 44], PCR [43, 44, 45], or cloning [44, 46, 47]. Third, a wide variety of nucleic acid‐modifying enzymes is available due to the advancements of molecular biology and cloning techniques [48, 49].

As a result, dissipative DNA structures were designed and fabricated relying on strand‐displacement reactions [50] with an emphasis on programming their lifetimes [51]. Aside from chemical fuels [52, 53, 54, 55, 56, 57, 58], DNA and RNA themselves have been used as fuels to drive the assembly of DNA‐based nanostructures [59, 60, 61, 62] in a dissipatively programmed manner. Apart from tuning the concentration of the fuel strands or the energy‐dissipating enzymes, a modular timer‐based [63] and a multi‐fuel‐reliant approach [64] have recently allowed further control over the kinetics of assemblies. Above all, notable efforts employing DNA have been made toward mimicking the complexity of natural reaction networks by incorporating bioactive and bioanalytical moieties [65, 66, 67, 68, 69, 70, 71] and fabricating dynamic multifunctional reaction networks through toe‐hold‐mediated strand displacement reactions (TMSD) [72], enzymatic reaction networks [73, 74], and dynamically gated and cascaded networks [75, 76, 77, 78]. Careful sequence design in these complex systems enables the programmed activation of multiple dormant functionalities. However, accessing each functionality typically requires either introducing different inputs sequentially or designing TMSD cascades to achieve orthogonal, transient activation of distinct functional states.

In our work, we connect two individual dissipative nucleic acid‐based systems to mimic information transfer in a cascade fashion, inspired by biological out‐of‐equilibrium systems. Specifically, we developed a novel feed‐forward approach to achieve autonomously regulated activation of a naturally occurring amino acid‐based enzyme. This is accomplished through the controlled activation of a nucleotide‐based synthetic enzyme, where the temporal activation of the amino acid enzyme is triggered by the transient activation of the synthetic DNA enzyme. These events occur within unidirectionally communicating DNA circuits governed by controlled information transfer. In the context of this work, we define autonomous behavior as the ability of a system, once triggered by a single fuel input, to undergo a self‐resetting cycle through the activation and deactivation of catalytic functions solely via internal, fuel‐dissipating reactions and preprogrammed design parameters, without any further external intervention. While catalytic transformations driven by transient responses within dissipative, dynamic DNA‐based networks have recently been explored by Willner and coworkers [60], the protein activation triggered by a transient information cascade described in this study has not yet been realized. Furthermore, the in situ generation of fuel to augment system autonomy and integration—addressing the reliance on multiple externally supplied fuels for precise control over functional behaviors—remains an unaddressed challenge in advanced design considerations. Additionally, we demonstrated that the kinetics of our interlinked system could be tuned by various pre‐programmed internal and external inputs.

**FIGURE 1:**
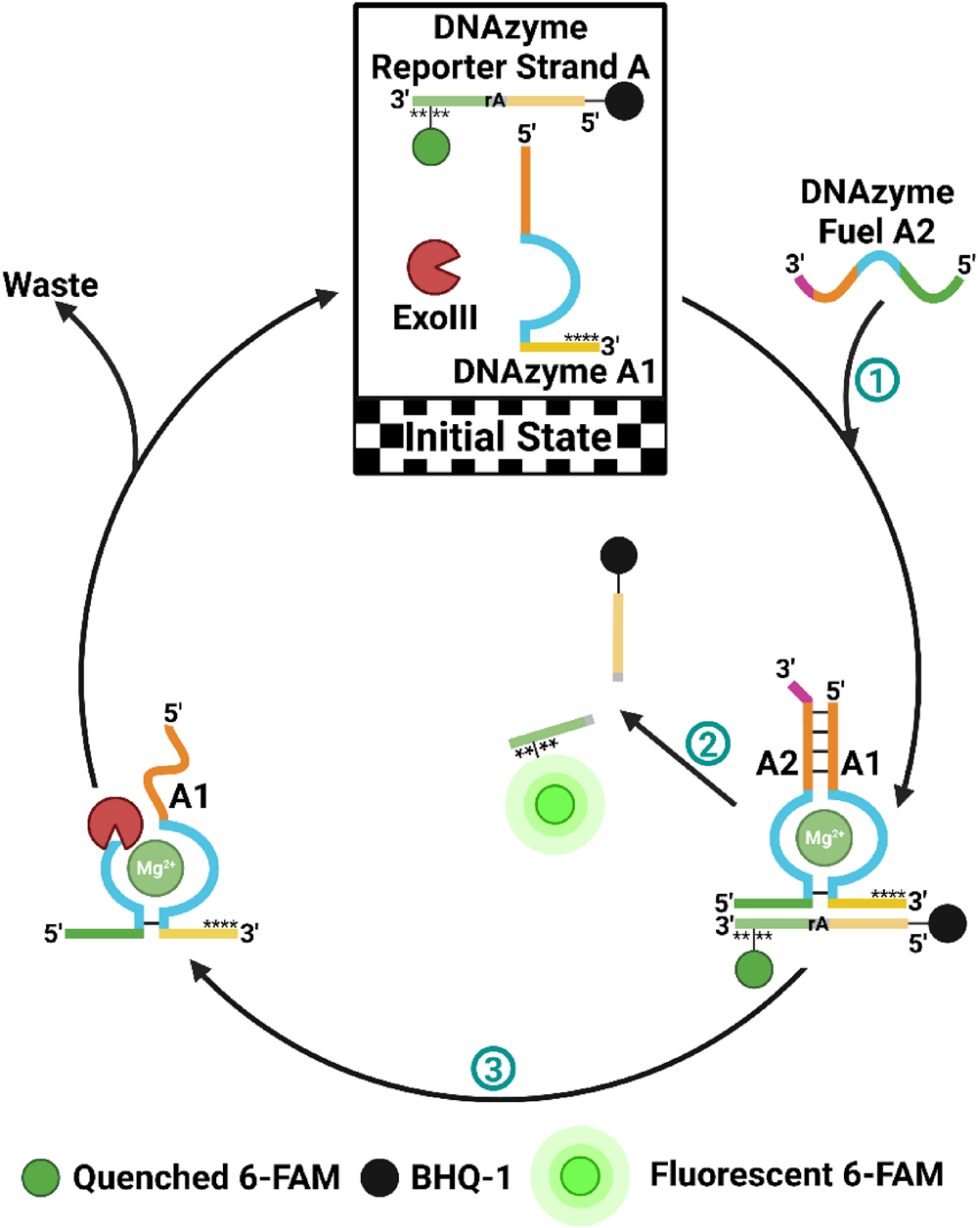
Schematic representation of Exo III‐governed dissipative regulation of the dsDNAzyme A1/A2. **Step 1**: Addition of DNAzyme fuel A2, leading to the formation of the active dsDNAzyme A1/A2. **Step 2**: Cleavage of the DNAzyme reporter strand A by the active dsDNAzyme A1/A2. **Step 3**: Dissipation of DNAzyme fuel A2 via Exo III digestion. Four consecutive phosphorothioate (PS) bonds at the 3′‐end protect DNAzyme A1 and the DNAzyme reporter strand A from Exo III digestion (indicated by four black stars). Created by Biorender.com.

To address these challenges, we developed a dissipatively active, DNA‐guided catalytic reaction network that converges to regulate the function of a protein enzyme. First, we describe two dissipative DNA‐based systems individually, then explain how they interact synergistically, with one system controlling the other to form a network. Our approach combines both experimental and computational methodologies. In the first part of our study, we demonstrate controlled manipulation of the catalytic activity of a DNAzyme (Figure 1) and the protein enzyme trypsin (Figure 3), each operating in individual cycles in the presence of exonuclease III (Exo III) or T7 exonuclease (T7) as fuel‐dissipating enzymes. The fuel dissipation results in the system attaining an out‐of‐equilibrium state. Precise control over the lifetime of the transient state was realized by regulating external measures, such as DNA fuel and exonuclease concentrations, and internally by exploiting the toe‐hold length‐dependent digestion kinetics of the exonucleases. The systems’ robustness in terms of fatigue resistance was investigated through several consecutive fuel additions.

In the second part, we introduce a novel network that operates dissipatively, enabling the precise and regulated activation of trypsin through the controlled activation of a synthetic DNAzyme (Figure 5). This was achieved by manipulating information transfer within the designed network. In this setup, communication occurs through the transient activation of the DNAzyme, which ultimately generates the fuel required for the temporal activation of trypsin (Figure 5). This was realized by employing an inhibitor strand capable of hybridizing with the dormant trypsin fuel strand. The inhibitor is designed to be susceptible to digestion by T7 after the cleavage of the phosphorothioate (PS) protection. The complete digestion of the inhibitor releases the trypsin fuel strand. The energy dissipation was materialized by involving Exo III, which selectively digests the DNA fuels. In this regard, it is worth highlighting the contribution of Su and coworkers [63], who exploited exonuclease activity to control the transient state of a DNA‐based dissipative system via the processive digestion of *λ*‐exonuclease (*λ* Exo). However, harnessing exonucleases to autonomously regulate biocatalytic pathways—specifically by directing the unidirectional propagation of information flow and terminating catalysis via the sequential, systematic manipulation of distinct enzymes operating across two interlinked DNA‐based circuits—remains uncharted territory. Precise control over the lifetime of the transient state of the DNAzyme and the trypsin cycle was achieved through DNAzyme fuel concentration, showcasing the influence of the lifetime of the DNAzyme system on the kinetics of the trypsin cycle. Additionally, we leveraged the intrinsic toe‐hold length‐dependent digestion kinetics of Exo III to preprogram the trypsin cycle's lifetime without altering the lifetime of the DNAzyme cycle. Lastly, we demonstrate control over the amount of released trypsin fuel strand by introducing a second competing substrate for the DNAzyme. The architectural flexibility of the network enables the envisioned creation of sophisticated DNA‐based circuits that can operate out‐of‐equilibrium, offering a high degree of programmability for targeted catalytic transformations.

## 2 Results and Discussion

### 2.1 Exonuclease‐Governed Dissipative Regulation of DNAzyme Activity

Since the discovery of the first DNAzyme three decades ago [79], numerous DNA‐based enzymes have been reported. These enzymes are capable of catalyzing a variety of reactions, including DNA/RNA cleavage and ligation, phosphorylation, dephosphorylation, ester and amide hydrolysis, Diels‐Alder reactions, and Friedel‐Crafts alkylation [22, 23, 24, 80]. Recently, there has been growing interest in utilizing these sequence‐dictated catalytic functions within out‐of‐equilibrium nucleic acid‐based dissipative circuits [58, 75, 81, 82, 83]. However, the feasibility of controlling DNAzyme activity through exonuclease‐based dissipative mechanisms remains unexplored. To investigate this, we selected a Mg^2+^‐ion‐dependent double‐stranded (ds) DNAzyme, capable of catalyzing the cleavage of a DNA reporter strand at an internal RNA modification site [84, 85]. Our design involves three key steps: first, the system starts in an inactive state, comprising one strand of the bipartite DNAzyme, the reporter strand, and the exonuclease (Figures 1 and S1, Initial State). The introduction of the second strand (fuel) triggers the formation of the active dsDNAzyme (Figures 1 and S1, Step 1), which then cleaves the reporter strand (Figures 1 and S1, Step 2). Subsequently, the DNAzyme deactivates due to the exonuclease digesting the fuel strand (Figure 1 and S1, Step 3), returning the system to its original dormant state. This state can be reactivated by adding more fuel strands. In this cycle, the exonuclease functions as the energy‐dissipating factor, enabling the system to operate in a dissipative manner. It is important to note that the reporter strand is irreversibly cleaved during each activation‐deactivation cycle, as it serves as the direct readout of DNAzyme activity. While this cleavage prevents the system from fully returning to its original chemical composition, the core regulatory components remain functionally intact, keeping the system resettable and enabling reuse across multiple cycles. We chose Exo III and T7 for the digestion of the DNA fuel. Both exonucleases catalyze the stepwise removal of mononucleotides from dsDNA with blunt ends and short single‐stranded (ss) overhangs. Exo III starts digestion from the 3′‐end, whereas T7 initiates at the 5′‐end of the DNA duplex [86, 87]. We deliberately selected this pair of exonucleases because their opposite directionalities prevent mutual interference and minimize the risk of unwanted digestion within the network. To ensure selective digestion of the DNA fuel, all other DNA strands were protected by introducing four consecutive PS bonds at either the 3′‐ or 5′‐end, depending on the exonuclease used [88]. Despite the PS‐modification, a swift increase in fluorescence was observed upon incubation of only the dye/quencher‐labeled DNAzyme reporter strand A′ with Exo III (Figure S2). DNAzyme reporter strand A′ contains 6‐Carboxyfluorescein (6‐FAM) at the 3′‐end and a black hole quencher (BHQ‐1) at the 5′‐end. Conversely, using the DNAzyme reporter strand A, which has an internal 6‐FAM modification and a BHQ‐1 modification at the 5′‐end, led to no increase in fluorescence in the presence of Exo III (Figure S2). This indicates that, despite the PS protection, Exo III was able to cleave the 3′‐end modification from the protected DNAzyme reporter strand A’, resulting in a rapid increase in fluorescence. For this reason, we decided to use DNAzyme reporter strand A with the internal 6‐FAM modification for all experiments conducted in the presence of Exo III. Unlike Exo III, T7 was unable to cleave the 5′‐end modification and therefore did not require any internal modification (Figure S2).

**FIGURE 2:**
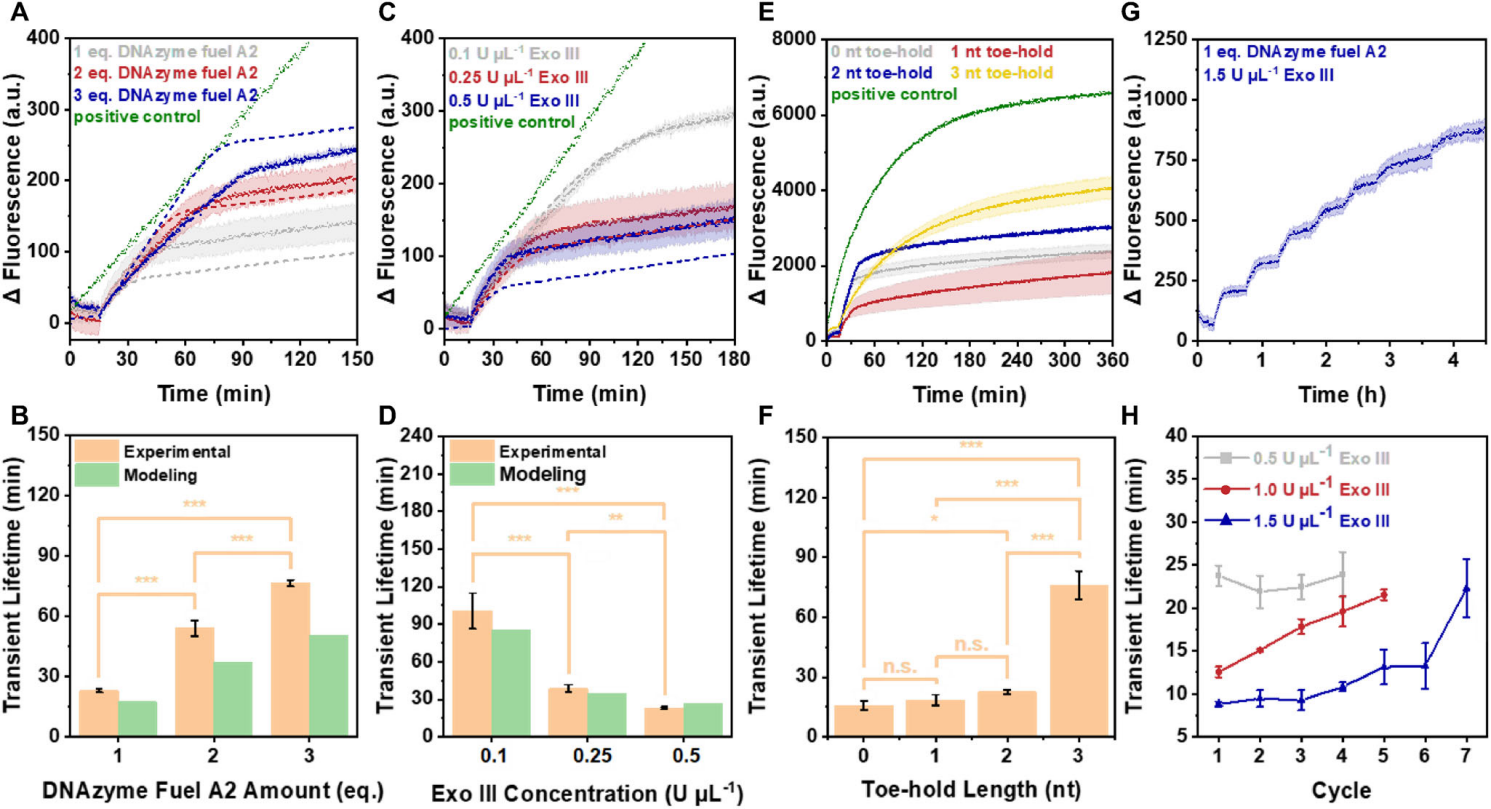
Dissipative control over the dsDNAzyme A1/A2 activity in the presence of Exo III. (A) Δ Fluorescence intensity at 520 nm after excitation at 490 nm plotted against time in the presence of 1, 2, and 3 eq. of DNAzyme fuel A2, DNAzyme reporter strand A (2.5 µM), and Exo III (0.5 U µL^−1^). Dashed lines represent simulated curves. (B) Transient lifetime of dsDNAzyme A1/A2 in the presence of different DNAzyme fuel A2 concentrations. Yellow: Transient lifetime determined from experimental data. Green: Transient lifetime determined from computationally generated curves. (C) Δ Fluorescence intensity at 520 nm after excitation at 490 nm plotted against time in the presence of 0.1, 0.25, and 0.5 U µL^−1^ Exo III, DNAzyme reporter strand A (2.5 µM), and DNAzyme fuel A2 (1 eq.). Dashed lines represent simulated curves. (D) Transient lifetime of dsDNAzyme A1/A2 in the presence of different Exo III concentrations. Yellow: Transient lifetime determined from experimental data. Green: Transient lifetime determined from computationally generated curves. (E) Δ Fluorescence intensity at 520 nm after excitation at 490 nm plotted against time after the addition of DNAzyme fuel A2 (1 eq.) containing toe‐holds of 0, 1, 2, and 3 nts in the presence of DNAzyme reporter strand A (2.5 µM) and Exo III (0.5 U µL^−1^). (F) Transient lifetime of dsDNAzyme A1/A2 in the presence of fuels containing different toe‐holds. (G) Successive additions of DNAzyme fuel A2 (1 eq.) in the presence of DNAzyme reporter strand A (2.5 µM), and Exo III (1.5 U µL^−1^) were performed at 15, 45.5, 76, 106, 137, 167.5, and 219 min. (H) Transient lifetime of dsDNAzyme A1/A2 (2.5 µM) for each consecutive fuel addition in the presence of 0.5, 1.0, and 1.5 U µL^−1^ Exo III. Data are presented as mean ± standard deviation (SD) of three independent experiments (n = 3); error bars represent the SD. The statistical significance of the experimentally obtained transient lifetimes was determined using analysis of variance (ANOVA). Significance levels are denoted as follows: n.s. = not significant; * = p < 0.05; ** = p < 0.01; *** = p < 0.001.

In the designed systems, only one strand of the dsDNAzyme acts as the DNA fuel (DNAzyme fuel A2 or B2). To note, DNA strands associated with the Exo III‐based dissipative cycle are labeled as A, A1, and A2 (Figure 1), whereas strands associated with the T7‐based dissipative system are designated as B, B1, and B2 (Figure S1). In the initial inactive state, no increase in the fluorescence signal at 520 nm, after excitation at 490 nm, was observed during the 15 min monitoring period (Figures 2A and S3). Upon the addition of the DNAzyme fuel (A2 or B2), the active dsDNAzyme is formed, leading to an immediate increase in fluorescence intensity (Figures 2A and S3). This increase occurs because the active dsDNAzyme catalyzes the cleavage of the DNAzyme reporter strand (A or B), resulting in the spatial separation of the fluorescent dye and the quencher. Subsequently, the digestion of the DNAzyme fuel (A2 or B2) by Exo III or T7 causes the autonomous deactivation of the system, as evidenced by the plateauing fluorescence intensity (Figures 2A and S3). The concentrations of fuel and exonuclease were chosen to ensure that strand hybridization—and thus the formation of the catalytically active DNAzyme—proceeded much faster than the subsequent digestion of the fuel by Exo III, thereby clearly separating the activation and deactivation phases. It is worth noting that the substrate cleavage capabilities of the employed dsDNAzymes under nondissipative conditions (i.e., in the absence of exonucleases) were also monitored (Figures S4 and S5). The results confirm that catalytic activity toward the reporter strand occurs only upon assembly of both strands into the active dsDNAzyme, and that exonuclease activity is required to render the system transient.

Following this, the ‘transient lifetime’, i.e., the time required for the system to return to its initial inactive state, was defined. Two key points were identified: the starting time when the fuel strand was added and the deactivation time, determined at the intersection of the linear fits for the initial increase after fuel addition and the plateau region after fuel consumption. The transient lifetime was then calculated by subtracting the time of fuel addition from the deactivation time (Figure S6). This quantitative parameter thus served as a unified criterion, enabling direct comparison of the kinetics of the dissipative cycle under varying reaction conditions, including fuel concentration, Exo III or T7 concentration, toe‐hold length of the fuel, and system recyclability. Precise control over the transient lifetime was achieved by varying the concentration of DNAzyme fuel A2 from 1 to 2 and 3 equivalents (eq.), resulting in a statistically significant prolongation of the transient lifetime from 23.0 to 54.1 and 76.4 min, respectively, in the presence of 0.5 U µL^−1^ Exo III (Figure 2A,B). In experiments with 2–3 eq. of fuel, we observe a slight upward drift of the signal during the apparent plateau phase. This effect is most pronounced at long experiment times and high fuel loadings and is likely attributed to a small fraction of residual fuel strands and partially digested intermediates that continue to be slowly processed by the exonuclease. These species give rise to ongoing low‐level formation of active catalyst and thus a gradual increase in reporter signal even after the main activation‐deactivation event has passed. Importantly, our transient lifetimes are determined from the intersection of linear fits to the initial rise and the early plateau, i.e., in a time window where this slow drift is minimal, so the upward drift has only a minor influence on the extracted lifetimes and does not affect the trends with fuel or exonuclease concentration. Conversely, increasing the Exo III concentration from 0.1 to 0.25 and 0.5 U µL^−1^ led to a statistically significant reduction in the transient lifetime from 100.6 to 38.8 and 23.0 min, respectively, at a constant DNAzyme fuel A2 concentration (1 eq.) (Figure 2C,D). To obtain a thorough insight into the designed systems, we further established a kinetic model that includes the formation of the catalytically active dsDNAzyme and its subsequent deactivation through the digestion of the DNAzyme fuel by Exo III or T7 (Eqn. S1–S4). The experimental data were simulated using this kinetic model, and the fitted predicted DNAzyme activity profiles were plotted as dashed curves (Figure 2A,C). Using the simulated kinetic parameters (Table S2), we could predict the system's transient lifetime depending on the concentrations of DNAzyme fuel A2 and Exo III (Figure 2B,D). Although the simulation slightly underestimates the transient lifetimes, the predicted curves are in good agreement with the experimental data. The higher transient lifetime values observed experimentally, especially in the presence of 2 and 3 eq. of DNAzyme fuel A2, can potentially be attributed to the influence of small, charged waste products (deoxynucleotide monophosphate) generated from Exo III fuel digestion. These waste products may interfere with the formation of the catalytically active secondary structure of the dsDNAzyme and the binding of the DNAzyme reporter strand to the dsDNAzyme. Additionally, they might contribute to the activity loss of Exo III due to nonspecific binding, thus reducing the effective Exo III concentration [89, 90, 91, 92]. All these factors together might have concomitantly contributed to the prolongation of the transient lifetime. This explanation is supported by the higher deviation of the transient lifetime from the prediction at lower Exo III concentrations, where the waste products would have a greater influence on the overall system, and at higher DNAzyme fuel concentrations, where larger amounts of waste are produced during one cycle.

Both Exo III and T7 are known to exhibit intrinsic toe‐hold length‐dependent digestion kinetics [86, 87]. Recognizing that Exo III can digest dsDNA with ss‐overhangs of up to four nucleotides (nts), we hypothesized that adding a short toe‐hold at the 3′‐end of the DNAzyme fuel would alter digestion kinetics, allowing intrinsic control over the system's lifetime. After thoroughly investigating the toe‐hold length‐dependent digestion kinetics of Exo III using polyacrylamide gel electrophoresis (PAGE) (Figure S7), we employed this molecular strategy in our DNAzyme system. We varied the 3′‐end toe‐hold length of the DNAzyme fuel A2 from 0 to 3 thymine units. Using the fully complementary fuel strand with no toe‐hold resulted in a transient lifetime of 15.7 min. No significant difference was observed with 1 and 2 nts toe‐holds, which produced transient lifetimes of 18.5 and 22.6 min, respectively. However, increasing the toe‐hold length to 3 nt significantly extended the lifetime to 76.1 min (Figure 2E,F). In the PAGE experiments, substrates bearing a 3‐nt toe‐hold were no longer detectably digested, whereas in the dissipative DNAzyme system, we still observe digestion but with strongly prolonged lifetimes. This difference is likely due to the much higher DNA concentrations required to obtain well‐resolved bands in PAGE, as well as differences in buffer composition between the gel and solution assays, both of which further disfavor Exo III processing under the PAGE conditions. We assume that the ss‐nature of the fuel strands with 1 and 2 nt toe‐holds does not differ sufficiently from the fuel without a toe‐hold, within the concentration range used in this study, to significantly affect digestion kinetics.

One characteristic feature of both natural and synthetic dissipative systems is their recyclability. After returning to the initial inactive state, the successive addition of the DNAzyme fuel A2 reactivated the system, allowing seven operational cycles in the presence of 1.5 U µL^−1^ Exo III (Figure 2G,H). During the first six cycles, the transient lifetime showed no significant increase, remaining between 8.8 min and 13.3 min. However, in the seventh cycle, it increased in a statistically relevant way to 22.3 min (Figure 2G,H, S8, S9, and Table S3). This overall increase in transient lifetime can be attributed to the continuous buildup of the aforementioned small, charged waste products generated during fuel digestion by Exo III, which interfered with the system [89, 90, 91, 92]. The simulated kinetic model of the system's recyclability aligned with the experimental data (Figures S8 and S9). As expected, the simulated transient lifetime did not increase over time, since the kinetic model does not account for the accumulation of waste products during consecutive cycles. This suggests that waste accumulation may contribute to system fatigue, allowing us to quantify the fatigue resistance of dissipative DNA‐based systems by comparing experimental data to the ideal simulation. Decreasing the Exo III concentration to 1.0 U µL^−1^ resulted in a more substantial and significant increase in transient lifetime, from 12.5 to 21.5 min over five cycles, underscoring the impact of waste buildup on system fatigue at lower Exo III concentrations (Figures 2H, S10, S11, and Table S4). Interestingly, at an Exo III concentration of 0.5 U µL^−1^, the transient lifetimes of 23.8, 21.9, 22.4, and 23.9 min remained relatively stable with each consecutive fuel addition. However, fluorescence enhancement systematically decreased with each cycle, and the slope of the plateau region increased, indicating that the system could not fully return to its initial deactivated state (Figures 2H, S12, S13, and Table S5). We attribute this behavior partly to the depletion of the fluorescent reporter: Due to the fact that reporter cleavage is irreversible and the reporter is not replenished, the first activation event consumes the largest fraction of available substrate, thereby producing the largest Δ fluorescence, while subsequent cycles generate progressively smaller Δ fluorescence values. Our kinetic model does not explicitly account for reporter depletion, as it was primarily designed to capture the lifetime of the transient activation rather than the absolute changes in Δ fluorescence observed with successive fuel additions.

Analogously, the control over DNAzyme activity in the presence of T7 was investigated (Figure S1). As expected, increasing the concentration of DNAzyme fuel B2 from 1 to 2 and 3 eq. resulted in a corresponding statistically significant increase in transient lifetime, from 42.7 to 95.2 and 147.7 min, respectively, in the presence of 0.5 U µL^−1^ T7 (Figures S3 and S14). Conversely, increasing the T7 concentration from 0.5 U µL^−1^ to 0.6 and 0.7 U µL^−1^, while keeping the DNAzyme fuel B2 concentration constant at 1 eq., led to a significant decrease in transient lifetime from 63.7 to 35.0 and 18.4 min (Figures S15 and S16), respectively. Consistent with the experimental data, simulations of the transient activity of dsDNAzyme matched these observations, showing greater deviations in transient lifetime with higher DNAzyme fuel concentrations and lower T7 concentrations due to the increased influence of waste products.

T7 is known to digest dsDNA with ss‐overhangs of up to 12 nts, which influences the digestion kinetics [87]. This was confirmed by PAGE (Figure S7). Subsequent investigations employing DNAzyme fuel B2 strands with 0, 5, 7, 10, and 12 thymine units (Figures S17 and S18) resulted in a statistically significant increase in transient lifetime, from 32.1 min for the fully complementary fuel to 44.4, 58.1, 78.3, and 94.9 min as the toe‐hold length increased in the presence of 0.6 U µL^−1^ T7.

Finally, we investigated the recyclability of the T7‐based system, which successfully completed up to six cycles. The transient lifetimes increased from 26.8 to 28.4, 32.3, 37.0, 40.7, and 52.2 min in the presence of 0.7 U µL^−1^ T7 (Figures S19 and S20), with the increase becoming statistically significant starting from the fourth fuel addition (Table S6). The gradual increase in transient lifetime, along with the growing deviation from the simulated curves and predicted transient lifetimes after each consecutive fuel addition, can be attributed to the buildup of waste products [89, 90, 91, 92]. The fatigue of the system was more pronounced at the lower T7 concentration of 0.6 U µL^−1^, where the transient lifetime increased from 41.1 to 54.7, 71.0, and 80.8 min over just four cycles (Figures S21, S22, and Table S7). At 0.5 U µL^−1^ T7, only three cycles were achieved, with transient lifetimes increasing from 65.4 to 76.2 and 154.4 min (Figures S23, S24, and Table S8).

### 2.2 Exonuclease‐Mediated Dissipative Control Over Trypsin Activity

**FIGURE 3:**
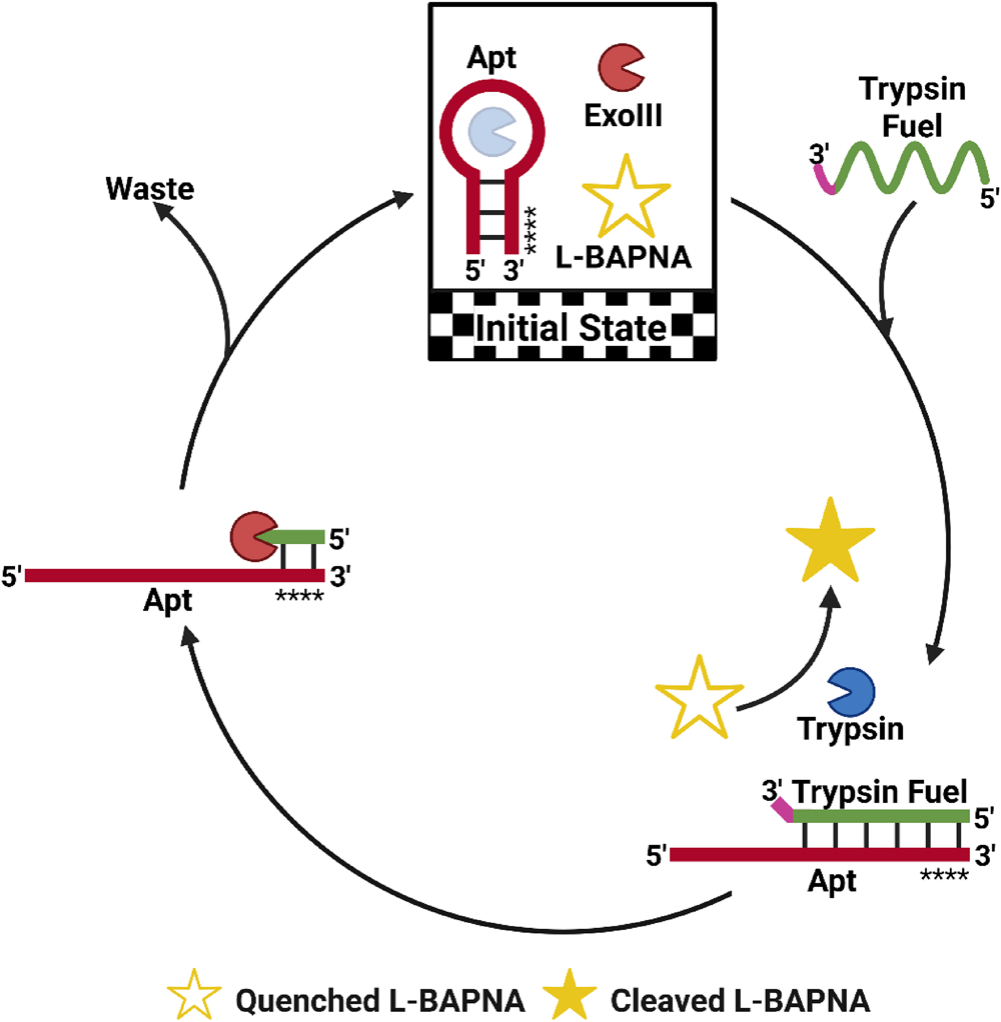
Schematic representation of Exo III‐governed dissipative regulation of trypsin. Step 1: Addition of the trypsin fuel activates trypsin. Step 2: Exo III digests the trypsin fuel, leading to dissipation. Step 3: Rebinding of the aptamer deactivates trypsin. Four consecutive PS bonds at the 3′‐end protect the trypsin aptamer from Exo III digestion (indicated by four black stars). Created by Biorender.com.

Next, we exploited our strategy to achieve temporal control over protein‐based enzyme activity, drawing inspiration from nature, where sophisticated processes have evolved to spatiotemporally regulate enzymes, ensuring activation only when necessary. Unintended enzyme activation can lead to life‐threatening diseases, so gaining precise control over enzyme activity can offer valuable insights into the underlying mechanisms [93]. Trypsin was selected as a model enzyme for the second dissipative cycle, primarily because of the availability of a well‐characterized trypsin‐binding aptamer and the simplicity of the proteolytic readout. Trypsin was initially deactivated through complexation with a ssDNA aptamer (Figures 3 and S25, Initial State) [94]. The transient activation of trypsin was achieved through the hybridization of the aptamer with a complementary trypsin fuel strand (Figures 3 and S25, Step 1). Nα‐Benzoyl‐L‐arginine 4‐nitroanilide hydrochloride (L‐BAPNA) was used as a reporter substrate to monitor trypsin activity. Upon cleavage of the amide bond adjacent to arginine, catalyzed by trypsin, the formation of p‐nitroaniline was detected at 405 nm [95]. The subsequent digestion of the fuel strand by Exo III or T7 (Figures 3 and S25, Step 2) enabled a temporal and autonomous activation‐deactivation cycle, driven by the rebinding of the dehybridized aptamer to trypsin, thereby regenerating the core regulatory components of the system (Figures 3 and S25, Step 3).

We first assessed the trypsin‐to‐aptamer ratio and the incubation time required for complete trypsin inactivation and subsequently used a 1:10 trypsin‐to‐aptamer ratio, as well as 30 min incubation time for all further experiments (Figures S26 and S27). Next, we evaluated the ability of the trypsin fuel to reactivate trypsin under nondissipative conditions. To minimize waste product formation during each cycle, we shortened the trypsin fuel to 30‐nt fragments. Upon adding the fuel strands, most fragments showed an immediate and gradual increase in absorbance at 405 nm, confirming successful trypsin reactivation (Figure S28). Consequently, trypsin fuel0‐30 was selected for all further experiments. The quantitative parameter, transient lifetime, was then defined (Figure S29) to compare kinetics under different reaction conditions, including fuel concentration, Exo III or T7 concentration, toe‐hold length of the fuel, and recyclability.

**FIGURE 4:**
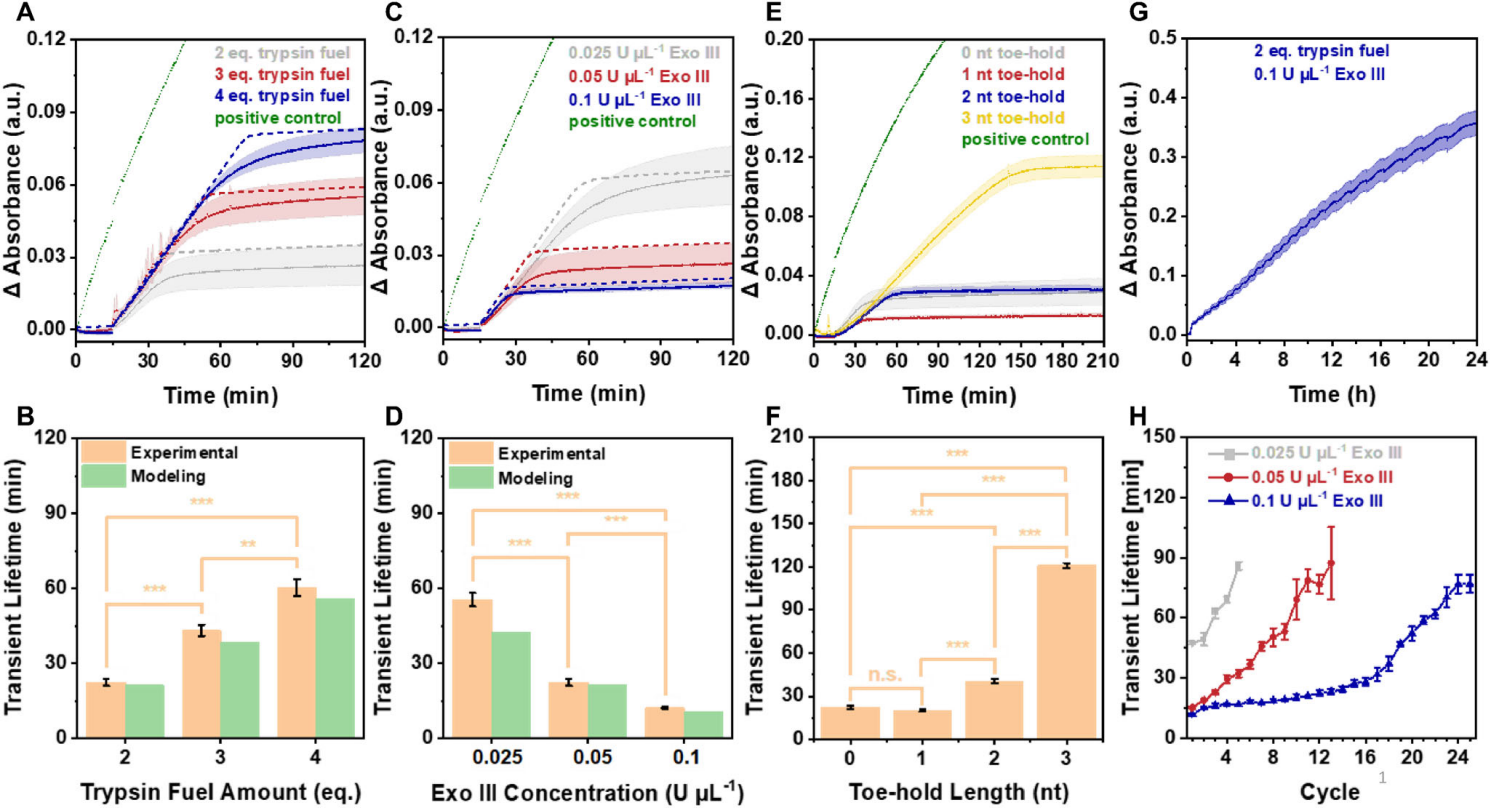
Dissipative control over trypsin activity in the presence of Exo III. (A) Δ Absorbance at 405 nm plotted against time for 2, 3, and 4 eq. of trypsin fuel, L‐BAPNA (100 µM), and Exo III (0.05 U µL^−1^). Dashed lines represent computationally simulated curves. (B) Transient lifetimes of trypsin with varying trypsin fuel concentrations. Yellow: Transient lifetime determined from the experimental data. Green: Transient lifetime obtained from computationally generated curves. (C) Δ Absorbance at 405 nm plotted against time for Exo III concentrations of 0.025, 0.05, and 0.1 U µL^−1^, L‐BAPNA (100 µM), and trypsin fuel (2 eq.). Dashed lines represent computationally simulated curves. (D) Transient lifetimes of trypsin with different Exo III concentrations. Yellow: Transient lifetime determined from the experimental data. Green: Transient lifetime obtained from computationally generated curves. (E) Δ Absorbance at 405 nm plotted against time after the addition of trypsin fuel (2 eq.) containing toe‐holds of 0, 1, 2, and 3 nts, in the presence of L‐BAPNA (100 µM) and Exo III (0.05 U µL^−1^). (F) Transient lifetimes of trypsin with trypsin fuel containing different toe‐holds. (G) Successive additions of trypsin fuel (2 eq.) in the presence of L‐BAPNA (100 µM), and Exo III (0.1 U µL^−1^) performed at 15, 40, 71, 107, 144, 180.5, 216, 252.5, 288.5, 323.5, 365, 407, 449, 488.5, 530.5, 576.5, 624, 677, 765.5, 806, 884, 968.5, 1058.5, 1158.5, and 1281.5 min. (H) Transient lifetime of trypsin for each consecutive fuel addition with Exo III concentrations of 0.025, 0.05, or 0.1 U µL^−1^. Data are presented as mean ± standard deviation (SD) of three independent experiments (n = 3); error bars represent the SD. The statistical significance of the experimentally obtained transient lifetimes was determined using analysis of variance (ANOVA). Significance levels: n.s. = not significant; * = p < 0.05; ** = p < 0.01; *** = p < 0.001.

Using a similar approach to the dsDNAzyme system described earlier, we investigated the kinetics of the transient activation of trypsin in the presence of Exo III as the fuel‐digesting enzyme (Figure 4). Increasing the trypsin fuel concentration from 2 to 3 and 4 eq., while keeping other parameters constant (0.1 µM trypsin, 1 µM aptamer, and 0.05 U µL^−1^ Exo III), resulted in a statistically significant increase in transient lifetime from 22.4 to 43.1 and 60.2 min, respectively (Figure 4A,B). Concomitantly, increasing the Exo III concentration from 0.025 to 0.05 and 0.1 U µL^−1^ led to a corresponding significant decrease in transient lifetime from 55.4 to 22.4 and 12.2 min, respectively (Figure 4C,D). Additionally, we developed a kinetic model to describe the transient activation of trypsin in the presence of Exo III or T7 (Eqn. S3–S7). The experimental data were simulated using this model, and the fitted predicted trypsin activity profiles were plotted as dashed curves (Figure 4A,C). Using the simulated kinetic parameters (Table S2), we could predict the system's transient lifetime based on the concentrations of trypsin fuel and Exo III (Figure 4B,D). For transient activation of trypsin in the presence of Exo III, the simulated curves closely matched the experimental data, with transient lifetimes falling within the error range of the experimentally determined values. The only exception was at the lowest Exo III concentration of 0.025 U µL^−1^, where the model underestimated the system's transient lifetime, likely due to its inability to account for the influence of waste products.

As observed in the DNAzyme system, introducing short toe‐holds (0‐3 adenine units) slowed Exo III digestion kinetics, leading to significantly prolonged transient lifetimes. These lifetimes ranged from 22.4 min for fuel without a toe‐hold to 20.2, 40.5, and 121.0 min as the toe‐hold length increased (Figure 4E,F). Notably, the use of a 1‐nt toe‐hold had no impact on the transient lifetime, a phenomenon also observed when analyzing Exo III digestion kinetics by PAGE (Figure S7). We assume that with only a 1 nt toe‐hold, the ss‐nature of the fuel does not differ enough from that of the fuel without a toe‐hold to slow down digestion kinetics. However, trypsin fuels with 2‐ or 3‐nt toe‐holds significantly increased the transient lifetime, with the 3‐nt toe‐hold producing a six‐fold increase.

Furthermore, the recyclability of the trypsin system was explored, achieving an impressive 25 cycles with just 0.1 U µL^−1^ Exo III. No significant increase in the transient lifetime was observed during the first 11 cycles, and it took 14 cycles to double the transient lifetime from 11.9 to 24.4 min. Even after 25 consecutive fuel additions, the system maintained the characteristic initial absorbance increase, taking 76.9 min to reach the plateau (Figures 4G,H, S30, S31, and Table S9). Despite greater fatigue at lower Exo III concentrations, 13 cycles were still achieved with 0.05 U µL^−1^ Exo III, with a statistically significant increase in transient lifetime starting after seven fuel additions, rising from 15.2 to 87.3 min (Figures 4H, S32, S33, and Table S10). Moreover, five cycles were possible with 0.025 U µL^−1^ Exo III, with transient lifetimes increasing significantly with the fifth cycle from 47.4 to 85.7 min (Figures 4H, S34, S35, and Table S11). The reason for the high fatigue resistance of the trypsin cycle might be the shortening of the trypsin fuel to 30 nts, which minimizes waste accumulation and allows optimal performance over multiple cycles. Consistent with this interpretation, an additional control experiment comparing the shortened 30‐mer fuel to a fully complementary 90‐mer fuel showed that the 30‐mer is digested markedly faster under identical conditions (Figure S36). The predicted outcome of consecutive trypsin fuel additions aligned closely with the experimental data, demonstrating that kinetic modeling can be a powerful tool in DNA‐fueled dissipative systems.

The system exhibited similar behavior in the presence of T7, though the fatigue resistance was less pronounced. Increasing the trypsin fuel concentration from 2 to 3 and 4 eq. significantly extended the transient lifetime from 17.6 to 24.4, and 35.6 min, respectively, in the presence of 0.6 U µL^−1^ T7 (Figures S37 and S38). As anticipated, increasing the T7 concentration from 0.4 to 0.5 to 0.6 U µL^−1^ notably reduced the transient lifetime from 39.2 to 29.3 and 17.6 min, respectively (Figures S39 and S40). The simulation of trypsin's transient activity aligns with the experimental data. However, due to the increasing influence of waste products, deviations in transient lifetime become more significant with higher trypsin fuel concentrations and lower T7 concentrations.

The introduction of toe‐holds with 0, 5, 7, 10, and 12 thymine units resulted in significant changes in transient lifetimes, ranging from 16.0 to 23.0, 30.4, 30.3, and 44.2 min, respectively, in the presence of 0.6 U µL^−1^ T7 (Figures S41 and S42) [87].

Although the fatigue resistance in the presence of T7 was less pronounced compared to Exo III, the experimental data were in good agreement with the predicted curves. Consequently, we could execute up to six cycles using 0.6 U µL^−1^ T7, and four cycles using 0.5 and 0.4 U µL^−1^ T7. This resulted in progressive and significant increases in transient lifetimes with each successive fuel addition, ranging from 15.9 to 71.2 min, 19.6 to 80.8 min, and 27.3 to 105.8 min, respectively (Figures S43–S48 and Tables S12–S14).

### 2.3 Unidirectionally Communicating DNA Circuits for Dissipatively Controlled Biocatalysis

**FIGURE 5:**
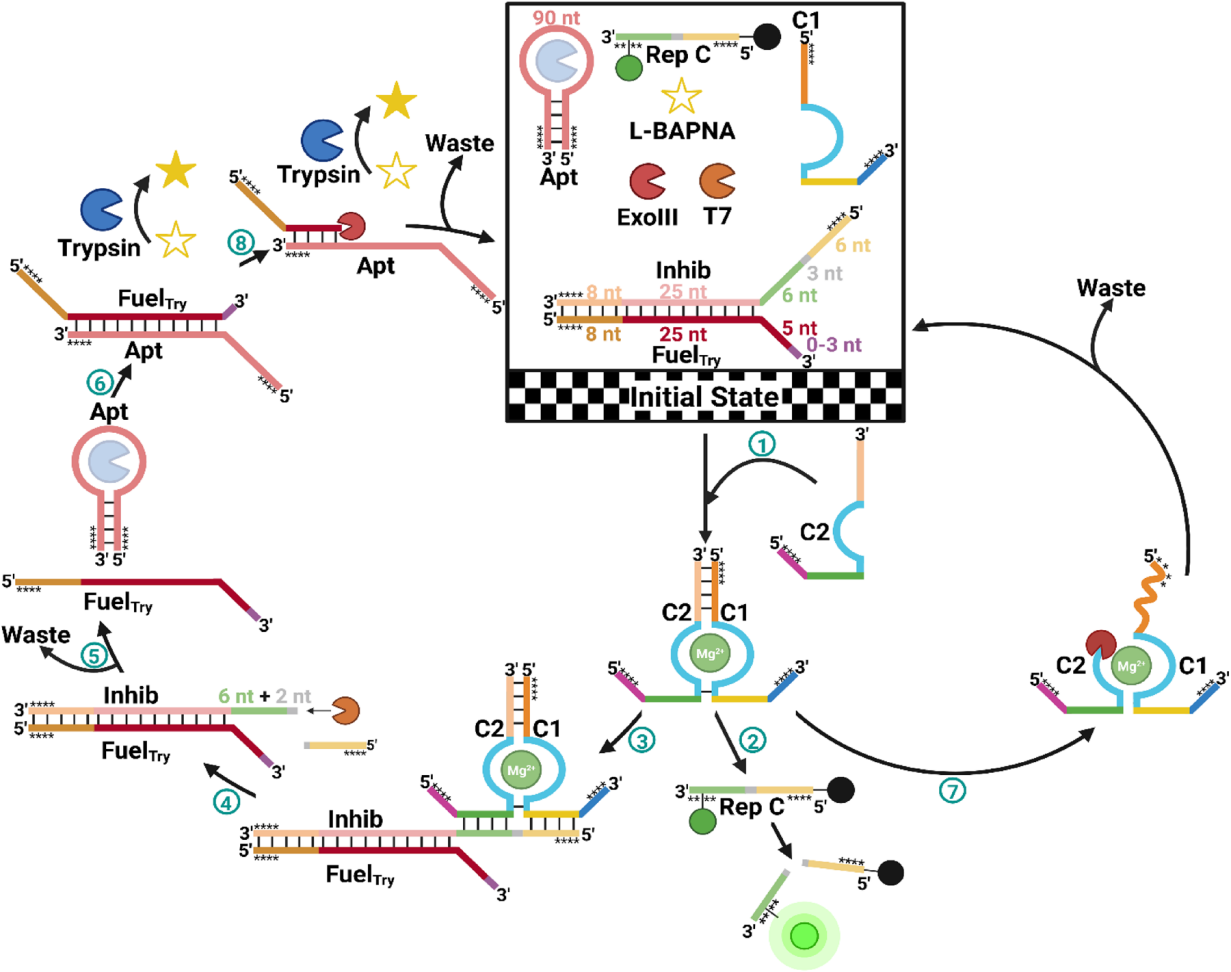
Schematic representation of dissipatively‐fueled unidirectionally communicating DNA circuits for controlled biocatalysis. In the system's initial state, the trypsin fuel is paired with an inhibitor strand, housing the recognition site for the dsDNAzyme. In addition to the deactivated trypsin fuel/inhibitor strand duplex, the initial state contains DNAzyme C1, L‐BAPNA, a small amount of DNAzyme reporter strand C, the aptamer‐bound trypsin, Exo III, and T7. **Step 1**: DNAzyme fuel addition, leading to the formation of the active dsDNAzyme. **Step 2**: Cleavage of the DNAzyme reporter strand by the active dsDNAzyme. **Step 3**: Binding of the dsDNAzyme to the recognition site of the inhibitor strand. **Step 4**: Cleavage of the inhibitor strand by the active dsDNAzyme. **Step 5**: Digestion of the inhibitor strand by T7, leading to trypsin fuel release. **Step 6**: Trypsin activation by hybridization of the released trypsin fuel and the trypsin aptamer. **Step 7**: DNAzyme fuel dissipation via Exo III digestion. **Step 8**: Trypsin fuel dissipation via Exo III digestion. Four consecutive PS bonds at the 3′‐end protect DNAzyme A1 and the DNAzyme reporter strand A from Exo III digestion (indicated by four black stars). Created by Biorender.com.

Inspired by natural energy‐dissipating, intercommunicating networks—such as the glycolytic pathway that powers, among others, Na^+^/K^+^‐ATPase ion pumps to maintain ion gradients across membranes [96]—we developed a dissipative DNA network for the autonomously controlled activation of a naturally occurring amino acid enzyme. This is achieved through the regulated activation of a synthetic, nucleotide‐based enzyme. The network operates upon the introduction of an oligodeoxynucleotide that serves as fuel for the transient activation of a dsDNAzyme (vide supra) present along with other components (vide infra), defining the initial state of this system. This activation subsequently generates the fuel for the temporal activation of trypsin (Figure 5). This DNA‐based communication for temporal control over enzyme activity not only mimics the sophistication of biological networks—predominantly operating in an energy influx‐guided, out‐of‐equilibrium manner—but also significantly contributes to contemporary efforts to exploit DNA‐guided relay and routing of information for the development of orthogonally operable DNA cascades [69] and enzymatic neural networks [97]. The interplay of four different enzymes (dsDNAzyme, trypsin, T7, and Exo III), along with multiple input signals such as fuel concentration, toe‐hold length, and competing substrates, programs the kinetics of the overall unidirectionally communicating circuits, showcasing the versatility of DNA‐based dissipative systems.

The first crucial step in the development of such a system was the design of the trypsin fuel strand (FuelTry) to prevent inadvertent activation of trypsin through uncontrolled hybridization with the trypsin aptamer (Apt) (Figure 5, Initial State). To achieve this, we opted for a hybridization strategy wherein FuelTry was paired with a partially complementary inhibitor strand (Inhib). Inhib, housing the recognition site of the dsDNAzyme C1/C2 at the 5′‐end, and FuelTry needed protection from digestion by Exo III and T7. Inhib was protected by introducing four PS‐bonds both at the 3′‐ and 5′‐ends, safeguarding it from the exonucleases. In contrast, four PS‐modifications at the 5′‐end protect FuelTry from T7 digestion, and a toe‐hold of five to eight nts at the 3′‐end was introduced to evade Exo III digestion. As the five to eight toe‐hold protection of the 3′‐end of FuelTry is part of the sequence complementary to Apt, additional base pairs are needed to thermodynamically stabilize the duplex, preventing spontaneous TMSD between FuelTry and Apt. In addition to this elaborately designed duplex, the initial state encompassed the 3′‐ and 5′‐end PS‐protected aptamer‐bound trypsin, the 3′‐ and 5′‐end PS‐protected DNAzyme C1, L‐BAPNA (trypsin substrate), and DNAzyme reporter strand C (Rep C), as well as Exo III and T7, as DNA‐digesting enzymes (Figure 5). The transient activation of the DNAzyme commenced with the addition of the 5′‐end PS‐protected DNAzyme fuel C2, leading to the formation of the active dsDNAzyme C1/C2 (Figure 5, Step 1). The presence of Rep C in the solution allowed monitoring of the activity of the transiently activated dsDNAzyme C1/C2 via the increase in fluorescence intensity at 520 nm (Figure 5, Step 2). Furthermore, binding of the dsDNAzyme C1/C2 to the recognition site of Inhib (Figure 5, Step 3) led to the dsDNAzyme C1/C2‐catalyzed cleavage at the RNA‐modification site, leaving Inhib with an eight‐nucleotide‐long 5′‐end toe‐hold while removing the part containing the PS‐protection, thus enabling the digestion of Inhib by T7 (Figure 5, Step 4) [84]. Subsequently, after complete digestion of the cleaved Inhib, FuelTry is released from its dormant state (Figure 5, Step 5), leading to the autonomous activation of trypsin through hybridization with Apt (Figure 5, Step 6). Concomitantly, the digestion of the 5′‐end protected DNAzyme fuel C2 by Exo III led to the autonomous deactivation of the DNAzyme and thus restricted the availability of FuelTry (Figure 5, Step 7). Once the availability of FuelTry was limited, digestion of FuelTry by Exo III from the 0 to 3 toe‐hold containing Apt/FuelTry duplex led to the autonomous deactivation of trypsin, through aptamer rebinding (Figure 5, Step 8).

Following the careful design of our interlinked system, we initially aimed to decrease the trypsin concentration and thus increase the sensitivity of the trypsin cycle, as DNAzymes are known to possess lower catalytic efficiency compared to their protein‐based counterparts (Figure S49) [98, 99, 100]. Additionally, we observed that the PS‐modification of the DNAzyme strands to assure selective digestion of the fuel strand only by Exo III led to a significant decrease in the catalytic activity of the dsDNAzyme D1/D2 (Figure S50). We hypothesize that, unlike in the previous DNAzyme system, introducing multiple PS‐bond protections within the recognition site slowed the hybridization between the dsDNAzyme D1/D2 and both the DNAzyme reporter strand C and the inhibitor strand, thereby reducing cleavage rates. To overcome the decrease in catalytic activity, we opted to extend DNAzyme C1 at the 3′‐end and the DNAzyme fuel strand C2 at the 5’‐end with five additional nts to avoid PS‐modification in the hybridization region and retain higher catalytic activity (Figure S50). We then assessed the potential for undesired activation of the initial state. Our design of the Inhib/FuelTry duplex effectively prevented spontaneous strand displacement in the absence of the dsDNAzyme C1/C2 (Figure S51). Upon adding DNAzyme C1, we observed no significant increase in fluorescence. However, a slight rise in absorbance hinted at possible strand displacement due to the hybridization of DNAzyme C1 and the recognition site of Inhib (Figure S52, red). Nonetheless, the addition of DNAzyme fuel C2 in the presence of only T7 resulted in an increase in fluorescence intensity and a notably higher absorbance at 405 nm, signifying the successful release of FuelTry after digestion of the cleaved inhibitor strand (Figure S52, blue). As Exo III functions as the fuel‐digesting enzyme of both the DNAzyme and the trypsin cycle, in its absence, the increase in absorbance and fluorescence only reached a plateau after the entirety of the L‐BAPNA and Rep C were cleaved. In contrast, when Exo III and T7 were both present, the absorbance and fluorescence reached a plateau well before the maximum intensities were reached (Figure S52, yellow).

**FIGURE 6:**
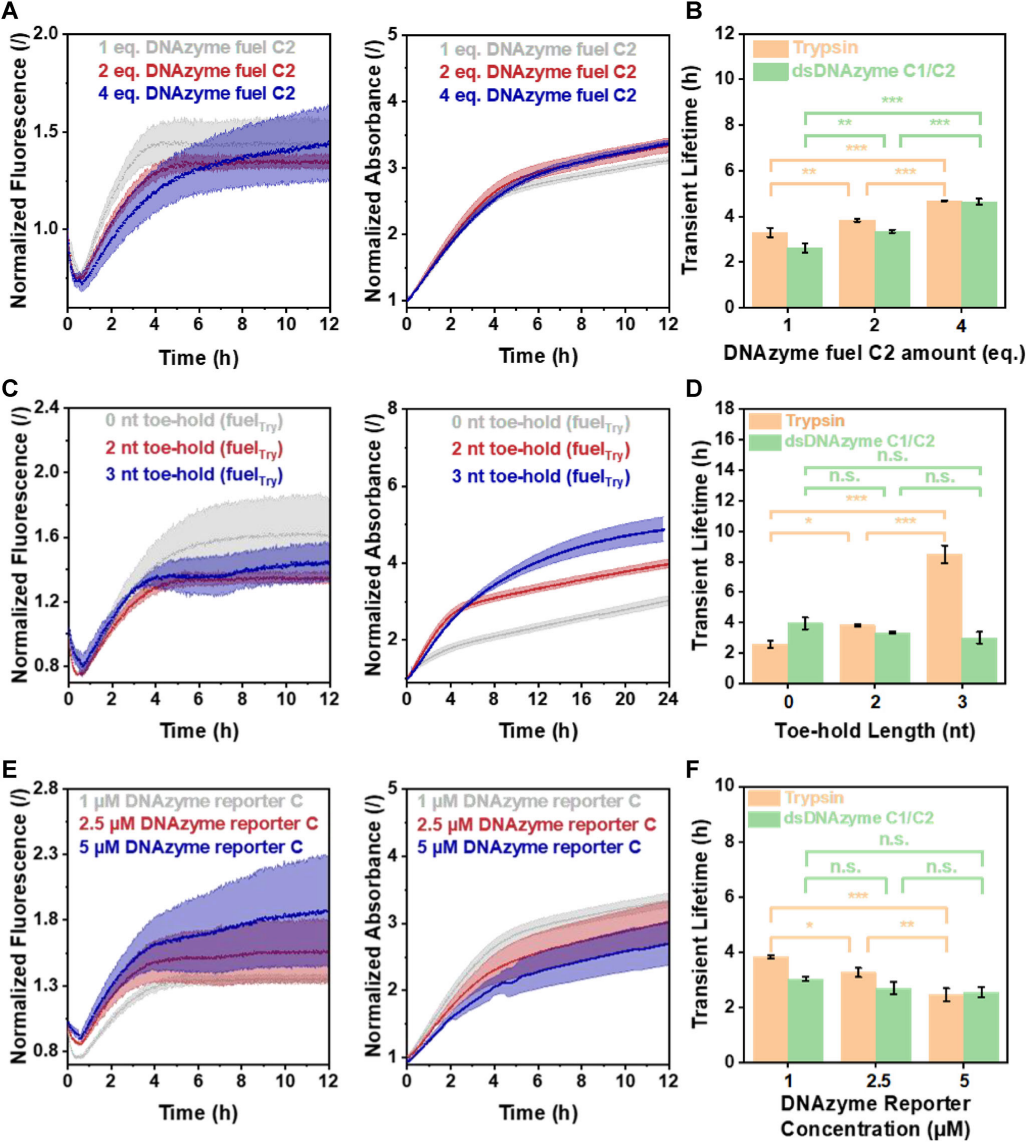
Dissipative orchestration of DNA fuel‐driven communication, where the transiently activated dsDNAzyme C1/C2 produces fuel for the dissipative activation of the protein enzyme trypsin. (A) The transient activity of the dsDNAzyme C1/C2, leading to the temporal activation of trypsin in the presence of 1, 2, or 4 eq. DNAzyme C2 fuel, DNA reporter strand C (1 µM), Exo III (0.025 U µL^−1^) and T7 (0.6 U µL^−1^), using trypsin fuel with a 2 nt toe‐hold. The normalized fluorescence at 520 nm after excitation at 490 nm plotted against the time monitors the temporal activity of dsDNAzyme C1/C2 (left panel), whereas the transient trypsin activity is monitored via the absorbance at 405 nm (right panel). (B) Transient lifetimes of the dsDNAzyme C1/C2 and trypsin in the presence of different amounts of DNAzyme fuel C2. (C) The transient activity of the dsDNAzyme D1/C2, leading to the temporal activation of trypsin in the presence of trypsin fuels with 3′‐end toe‐holds of 0, 2, or 3 nts, using DNAzyme fuel C2 (2 eq.), DNA reporter strand C (1 µM), Exo III (0.025 U µL^−1^), and T7 (0.6 U µL^−1^). Normalized fluorescence at 520 nm after excitation at 490 nm plotted against the time monitors the temporal activity of dsDNAzyme C1/C2 (left panel), whereas the transient trypsin activity is monitored via the absorbance at 405 nm (right panel). (D) Transient lifetimes of the dsDNAzyme C1/C2 and trypsin in the presence of trypsin fuels with different toe‐holds. (E) Transient activity of the dsDNAzyme C1/C2, leading to the temporal activation of trypsin in the presence of 1, 2.5, or 5 µM DNA reporter strand C, DNAzyme fuel C2 (2 eq.), Exo III (0.025 U µL^−1^), and T7 (0.6 U µL^−1^), using trypsin fuel with a 2 nt toe‐hold. Normalized fluorescence at 520 nm after excitation at 490 nm plotted against the time monitors the temporal activity of dsDNAzyme C1/C2 (left panel), whereas the transient trypsin activity is monitored via the absorbance at 405 nm (right panel). (F) Transient lifetimes of the dsDNAzyme C1/C2 and trypsin in the presence of different DNA reporter strand C concentrations. Data are presented as mean ± standard deviation (SD) of three independent experiments (n = 3); error bars represent the SD. The statistical significance of the experimentally obtained transient lifetimes was determined using analysis of variance (ANOVA). Significance levels: n.s. = not significant; * = p < 0.05; ** = p < 0.01; *** = p < 0.001.

With the successful orchestration of communication‐mediated activation in the second dissipative system, we systematically tuned various parameters to highlight control over the system's kinetics. By altering the concentration of DNAzyme fuel C2 from 1 to 2 and 4 eq., we aimed to manipulate the transient lifetime of both the DNAzyme and the trypsin cycle (Figure 6A). The fluorescence and absorbance of the initial state were monitored for 30 min before DNAzyme fuel C2 addition. Notably, the fluorescence intensity initially decreased, which was attributed to the insufficient mixing of components due to the presence of glycerol in the enzyme storage buffers and DMSO in the L‐BAPNA stock solution. Furthermore, some strand displacement due to the presence of DNAzyme C1 in the initial state can be observed by the slight initial increase in absorbance at 405 nm. The subsequent addition of DNAzyme fuel C2 resulted in an immediate and significant fluorescence and absorbance intensity increase. As expected, higher concentrations of DNAzyme fuel C2 led to statistically significant increases in the transient lifetimes of both the DNAzyme and trypsin cycles, with lifetimes increasing from 2.6 to 3.4 and 4.6 h for the DNAzyme, and from 3.3 to 3.8 and 4.7 h for trypsin (Figure 6B).

Additionally, the introduction of 3′‐end toe‐holds to the trypsin fuel was explored. Prehybridizing the inhibitor strand with trypsin fuels, featuring no additional toe‐hold, as well as 2 and 3 adenine units at the 3′‐end, revealed altered kinetics for the transient trypsin activation without changing the kinetics of the DNAzyme cycle (Figure 6C). Increasing the 3′‐end toe‐hold length resulted in a substantial increase in the transient lifetime, from 2.6 h for the trypsin fuel without an additional toe‐hold, to 3.8 and 8.5 h with 2‐ and 3‐nt toe‐holds, respectively. In contrast, the transient lifetime of the dsDNAzyme C1/C2 decreased, though not significantly, from 4.0 to 3.4 and 3.0 h (Figure 6D).

Lastly, the DNA reporter strand concentration might influence the kinetics of the trypsin cycle because it acts as a competitive substrate to Inhib. Increasing the availability of Rep C while keeping the Inhib/FuelTry duplex and dsDNAzyme C1/C2 concentrations constant should decrease the probability of inhibitor cleavage due to increased competition. For this, we investigated three different Rep C concentrations of 1, 2.5, and 5 µM (Figure 6E). Monitoring the absorbance at 405 nm revealed that the transient lifetime of trypsin significantly decreased with increasing DNA reporter strand concentration, from 3.8 to 3.3 and 2.5 h. Meanwhile, the transient lifetime of the dsDNAzyme C1/C2 showed a nonstatistically significant decrease from 3.0 to 2.7 and 2.6 h (Figure 6F).

## 3 Conclusion

This study introduces a unique, feed‐forward, DNA‐based information‐transfer framework that operates out‐of‐equilibrium, enabling the autonomous activation of a naturally occurring protein enzyme through the controlled, upstream activation of a nucleotide‐based synthetic enzyme. This process is governed by the precise, unidirectional transfer of information. While a recent study has demonstrated information routing within constitutional dynamic networks (CDNs) composed of nucleic acid‐enzyme conjugates acting as modules for triggered, network‐driven biocatalytic cascades and their intercommunication [101], we distinctively exploit this fundamental aspect of biological circuits. Specifically, we relay and process information in a way that controls biochemical reactions and pathways, thereby activating protein activity guided by DNA circuits. In the first part of this report, we discuss individual cycles designed for dissipatively controlling the catalytic activity of a metal‐dependent DNAzyme and the protein enzyme trypsin. By rationally designing the fuel strand, we achieved considerable control over the transient activation of a dsDNAzyme and temporal control over the enzymatic activity of aptamer‐bound trypsin. The transient out‐of‐equilibrium state was realized by adding a DNA fuel strand, with the digestion of the fuel by Exo III or T7 serving as the energy‐dissipating step. Significant control over the kinetics of the temporal activation of a Mg^2+^‐dependent dsDNAzyme was achieved not only through the concentrations of DNA fuel and fuel‐dissipating enzymes but also via the toe‐hold length‐dependent digestion kinetics of Exo III and T7. The design of toe‐hold‐bearing fuels represents a universal and straightforward tool for implementation in various out‐of‐equilibrium systems to gain additional control over the system's kinetics. The use of toe‐hold‐containing fuels could mitigate the effects of waste product buildup on the system fatigue of dissipative self‐assembling systems, as lesser amounts of DNA fuel are needed to achieve longer transient lifetimes. In regard to system fatigue, we further established that shortening the length of the DNA fuel increases fatigue resistance, monitored in the presence of very low concentrations of Exo III. Furthermore, we established a kinetic model to predict the dissipative activation of the dsDNAzyme and trypsin in the presence of Exo III and T7 as fuel‐digesting enzymes, providing greater insights into our systems. More importantly, simulations of recyclability allowed us to evaluate fatigue resistance and gain further understanding of the role of waste product accumulation in system fatigue. Although a quantitative assessment of the influence of small, charged waste products generated by exonuclease‐mediated digestion on system fatigue would require a more systematic investigation beyond the scope of the present work, we note potential strategies to improve recyclability in DNA‐fueled dissipative systems. Incorporating waste‐degrading enzymes, minimizing fuel consumption through sequence and toe‐hold design, and implementing flow‐based approaches, where fresh enzymes are continuously supplied while small waste products are removed, may provide promising routes toward more fatigue‐resistant dissipative DNA networks.

To conclude, we have combined the individually operating dissipative cycles (Figures 1 and 3) into an unidirectionally communicating network (Figure 5), characterized by temporally‐controlled directed flow of information and exceptional multifactorial control (vide supra) as well as system autonomy—thereby achieving a new level of advanced, synthetic, out‐of‐equilibrium biocatalytic self‐assembly. Our work expands the scope of programming DNA circuits in the context of DNA‐fueled, dissipatively orchestrated self‐assembling systems. This advancement will significantly benefit the development of functional, dynamic, and out‐of‐equilibrium self‐assemblies for dictated biocatalytic transformations. The growing number of newly discovered DNAzymes, which can catalyze a wide variety of reactions, along with novel aptamers [102] capable of binding and inhibiting different enzymes, will enable the creation of more biologically relevant dissipative self‐assembling systems. These systems may ultimately aid in understanding the naturally occurring, highly efficient spatiotemporal regulation of enzymes, which is crucial for maintaining physiological balance. For example, unintended activation of trypsinogen in the pancreas can lead to severe and life‐threatening pancreatitis [93]. Therefore, achieving reversible and intercommunicable control over enzyme activity could be key to engineering complex biological processes and, in the long term, preventing such pathological conditions. These advancements introduce fundamental concepts to the field of systems chemistry by providing concatenated, dynamic model systems that emulate core features of natural biochemical networks.

## Conflicts of Interest

The authors declare no conflicts of interest.

## Code Availability

This paper does not report original code.

## Supporting information



The authors have cited additional references within the Supporting Information [1, 2].

## Data Availability

All data related to this paper will be shared by the corresponding authors upon reasonable request. This study did not generate new unique materials or reagents.
