# Supplementary material for: Dissipatively Fueled Unidirectionally Communicating DNA Circuits That Control Biocatalysis
Source: Angew Chem Int Ed Engl. 2026 Mar 12;65(17):e23416. doi: 10.1002/anie.202523416 (PMC13098476; doi:10.1002/anie.202523416)
Supplement: Supplementary file 1 — The authors have cited additional references within the Supporting Information [1, 2]. [file ANIE-65-e23416-s001.docx]

**Supporting Information for**

**Dissipatively Fueled** **Unidirectionally Communicating DNA Circuits That Control Biocatalysis**

Philippe Jung, Daniel Felder, Gurudas Chakraborty*, Tim Seifert, Matthias Wessling, Lifei Zheng* and Andreas Herrmann*

*Correspondence: chakraborty@dwi.rwth-aachen.de, [zhenglf@ucas.ac.cn](mailto:zhenglf@ucas.ac.cn), [herrmann@dwi.rwth-aachen.de](mailto:herrmann@dwi.rwth-aachen.de)

**Table of contents**

| - Recurring Abbreviations | S6 |
| --- | --- |
| - Materials | S7 |
| - General | S7 |
| - Chemicals | S7 |
| - Buffers | S7 |
| - Oligonucleotides | S7 |
| - Supplemental Methods | S11 |
| - Analysis of Exonuclease Dissipation Kinetics via Polyacrylamide Gel Electrophoresis | S11 |
| - Standard procedure for the dissipative activation of dsDNAzyme A1/A2 or B1/B2 in the presence of Exo III or T7 | S11 |
| - Standard procedure for the dissipative activation of bovine trypsin in the presence of Exo III or T7 | S12 |
| - Standard procedure for the interlinked DNA system for dissipative control over biocatalysis | S12 |
| - Toe-hold-dependent DNA duplex digestion kinetics with Exo III and T7 | S13 |
| - Polyacrylamide gel electrophoresis (PAGE) | S13 |
| - Kinetic Modeling | S13 |
| - Simulation and parameter estimation for the enzyme kinetics | S13 |
| - DNAzyme system | S14 |
| - Trypsin system | S15 |
| - Table S2. Simulated kinetic parameters | S15 |
| - Supplementary Figures and Tables | S16 |
| - Figure S1. Schematic representation of T7-governed dissipative regulation of the dsDNAzyme B1/B2. | S16 |
| - Figure S2. Stability of the DNAzyme reporter strand modifications in the presence of Exo III or T7. | S17 |
| - **Figure S3. Dissipative control over dsDNAzyme B1/B2 activity in the presence of T7 and different amounts of DNAzyme fuel B2.** | S18 |
| - Figure S4. Normalized fluorescence intensity at 520 nm over time after excitation at 490 nm for ssDNAzyme A1, ssDNAzyme A2, and dsDNAzyme A1/A2 under non-dissipative conditions. | S19 |
| - Figure S5. Normalized fluorescence intensity at 520 nm over time after excitation at 490 nm for ssDNAzyme B1, ssDNAzyme B2, and dsDNAzyme B1/B2 under non-dissipative conditions. | S20 |
| - Figure S6. Graphical determination of the transient lifetime exemplified with the Exo III-regulated dsDNAzyme A1/A2 system. | S21 |
| - Figure S7. Design and evaluation of toe-hold-dependent DNA duplex digestion by Exo III or T7. | S22 |
| - Figure S8. Successive additions of the ssDNAzyme fuel A2 in the presence of 1.5 U µL^-1^ Exo III. | S24 |
| - Figure S9. Transient lifetime of the dsDNAzyme A1/A2 in the presence of 1.5 U µL^-1^ Exo III as a function of consecutive fuel additions. | S25 |
| - Table S3. Statistical significance determined by analysis of variance (ANOVA) using the Origin 2024b software for the successive additions of the ssDNAzyme fuel A2 in the presence of 1.5 U µL^-1^ Exo III. | S26 |
| - Figure S10. Successive additions of the ssDNAzyme fuel A2 in the presence of 1.0 U µL^-1^ Exo III. | S27 |
| - Figure S11. Transient lifetime of the dsDNAzyme A1/A2 in the presence of 1.0 U µL^-1^ Exo III as a function of consecutive fuel additions. | S28 |
| - Table S4. Statistical significance determined by analysis of variance (ANOVA) using the Origin 2024b software for the successive additions of the ssDNAzyme fuel A2 in the presence of 1.0 U µL^-1^ Exo III. | S29 |
| - Figure S12. Successive additions of the ssDNAzyme fuel A2 in the presence of 0.5 U µL^-1^ Exo III. | S30 |
| - Figure S13. Transient lifetime of the dsDNAzyme A1/A2 in the presence of 0.5 U µL^-1^ Exo III as a function of consecutive fuel additions. | S31 |
| - Table S5. Statistical significance determined by analysis of variance (ANOVA) using the Origin 2024b software for the successive additions of the ssDNAzyme fuel A2 in the presence of 0.5 U µL^-1^ Exo III. | S32 |
| - **Figure S14.** Transient lifetimes of dsDNAzyme B1/B2 after adding different amounts of ssDNAzyme fuel B2. | S33 |
| - **Figure S15. Dissipative control over dsDNAzyme B1/B2 activity in the presence of different T7 concentrations.** | S34 |
| - **Figure S16.** Transient lifetimes of dsDNAzyme B1/B2 in the presence of different T7 concentrations. | S35 |
| - **Figure S17. Dissipative control over dsDNAzyme B1/B2 activity using ssDNAzyme fuel B2 with different toe-hold lengths.** | S36 |
| - **Figure S18.** Transient lifetimes of dsDNAzyme B1/B2 **using ssDNAzyme fuel B2 with different toe-hold lengths**. | S37 |
| - Figure S19. Successive additions of the ssDNAzyme fuel B2 in the presence of 0.7 U µL^-1^ T7. | S38 |
| - Figure S20. Transient lifetime of the dsDNAzyme B1/B2 in the presence of 0.7 U µL^-1^ T7 as a function of consecutive fuel additions. | S39 |
| - Table S6. Statistical significance determined by analysis of variance (ANOVA) using the Origin 2024b software for the successive additions of the ssDNAzyme fuel B2 in the presence of 0.7 U µL^-1^ T7. | S40 |
| - Figure S21. Successive additions of the ssDNAzyme fuel B2 in the presence of 0.6 U µL^-1^ T7. | S41 |
| - Figure S22. Transient lifetime of the dsDNAzyme B1/B2 in the presence of 0.6 U µL^-1^ T7 as a function of consecutive fuel additions. | S42 |
| - Table S7. Statistical significance determined by analysis of variance (ANOVA) using the Origin 2024b software for the successive additions of the ssDNAzyme fuel B2 in the presence of 0.6 U µL^-1^ T7. | S43 |
| - Figure S23. Successive additions of the ssDNAzyme fuel B2 in the presence of 0.5 U µL^-1^ T7. | S44 |
| - Figure S24. Transient lifetime of the dsDNAzyme B1/B2 in the presence of 0.5 U µL^-1^ T7 as a function of consecutive fuel additions. | S45 |
| - Table S8. Statistical significance determined by analysis of variance (ANOVA) using the Origin 2024b software for the successive additions of the ssDNAzyme fuel B2 in the presence of 0.5 U µL^-1^ T7. | S46 |
| - Figure S25. Schematic representation of the dissipative activation of the protein enzyme trypsin in the presence of T7. | S47 |
| - Figure S26. Determination of the aptamer-to-trypsin ratio required for the complete deactivation of trypsin. | S48 |
| - Figure S27. Incubation time required for the complete deactivation of trypsin with 10 eq. of aptamer. | S49 |
| - Figure S28. Shortening of the trypsin fuel. | S50 |
| - Figure S29. Example for the graphical determination of the transient lifetime of trypsin in the presence of Exo III via the intersection point of the linearly fitted regions of the curve. | S51 |
| - Figure S30. Successive additions of trypsin fuel in the presence of 0.1 U µL^-1^ Exo III. | S52 |
| - Figure S31. Transient lifetime of trypsin in the presence of 0.1 U µL^-1^ Exo III as a function of consecutive fuel additions. | S53 |
| - Table S9. Statistical significance determined by analysis of variance (ANOVA) using the Origin 2024b software for the successive additions of trypsin fuel in the presence of 0.1 U µL^-1^ ExoIII. | S54 |
| - Figure S32. Successive additions of trypsin fuel in the presence of 0.05 U µL^-1^ Exo III. | S56 |
| - Figure S33. Transient lifetime of trypsin in the presence of 0.05 U µL^-1^ Exo III as a function of consecutive fuel additions. | S57 |
| - Table S10. Statistical significance determined by analysis of variance (ANOVA) using the Origin 2024b software for the successive additions of trypsin fuel in the presence of 0.05 U µL^-1^ Exo III. | S58 |
| - Figure S34. Successive additions of trypsin fuel in the presence of 0.025 U µL^-1^ Exo III. | S59 |
| - Figure S35. Transient lifetime of trypsin in the presence of 0.025 U µL^-1^ Exo III as a function of consecutive fuel additions. | S60 |
| - Table S11. Statistical significance determined by analysis of variance (ANOVA) using the Origin 2024b software for the successive additions of trypsin fuel in the presence of 0.025 U µL^-1^ Exo III. | S61 |
| - Figure S36. Dissipative control over trypsin activity in the presence of Exo III using two different trypsin fuels: a shortened 30-mer and a 90-mer fully complementary to the trypsin aptamer. | S62 |
| - **Figure S37. Dissipative control over trypsin activity in the presence of T7 and different amounts of trypsin fuel.** | S63 |
| - **Figure S38.** Transient lifetimes of trypsin after adding different amounts of trypsin fuel. | S64 |
| - **Figure S39. Dissipative control over trypsin activity in the presence of different T7 concentrations.** | S65 |
| - **Figure S40.** Transient lifetimes of trypsin in the presence of different T7 concentrations. | S66 |
| - **Figure S41. Dissipative control over trypsin activity in the presence of trypsin fuels with different toe-hold lengths.** | S67 |
| - **Figure S42.** Transient lifetimes of trypsin **in the presence of trypsin fuels with different toe-hold lengths**. | S68 |
| - Figure S43. Successive additions of trypsin fuel in the presence of 0.6 U µL^-1^ T7. | S69 |
| - Figure S44. Transient lifetime of trypsin in the presence of 0.6 U µL^-1^ T7 as a function of consecutive fuel additions. | S70 |
| - Table S12. Statistical significance determined by analysis of variance (ANOVA) using the Origin 2024b software for the successive additions of trypsin fuel in the presence of 0.6 U µL^-1^ T7. | S71 |
| - Figure S45. Successive additions of trypsin fuel in the presence of 0.5 U µL^-1^ T7. | S72 |
| - Figure S46. Transient lifetime of trypsin in the presence of 0.5 U µL^-1^ T7 as a function of consecutive fuel additions. | S73 |
| - Table S13. Statistical significance determined by analysis of variance (ANOVA) using the Origin 2024b software for the successive additions of trypsin fuel in the presence of 0.5 U µL^-1^ T7. | S74 |
| - Figure S47. Successive additions of trypsin fuel in the presence of 0.4 U µL^-1^ T7. | S75 |
| - Figure S48. Transient lifetime of trypsin in the presence of 0.4 U µL^-1^ T7 as a function of consecutive fuel additions. | S76 |
| - Table S14. Statistical significance determined by analysis of variance (ANOVA) using the Origin 2024b software for the successive additions of trypsin fuel in the presence of 0.4 U µL^-1^ T7. | S77 |
| - Figure S49. Reduction of the trypsin concentration. | S78 |
| - Figure S50. Comparison of the RNA cleavage kinetics of the extended dsDNAzyme C1/C2 and the non-extended dsDNAzyme D1/D2. | S79 |
| - Table S15. Summary of the sample compositions corresponding to the colored traces in Figure S51. | S81 |
| - Figure S51. Control experiments are conducted to ensure that no unwanted spontaneous strand displacement between the inhibitor/trypsin fuel duplex and the aptamer occurs in the presence or absence of Exo III and T7. | S82 |
| - Table S16. Summary of the sample compositions corresponding to the colored traces in Figure S52. | S84 |
| - Figure S52. Proof of concept for the dissipative control over a biocatalytic DNA cascade reaction. | S85 |
| - References | S86 |

**Recurring Abbreviations**

**nts** nucleotides

**ssDNA** single-stranded deoxyribonucleic acid

**dsDNA** double-stranded deoxyribonucleic acid

**RNA** ribonucleic acid

**Exo III** exonuclease III

**T7** T7 exonuclease

**6-FAM** 6-Carboxyfluorescein

**BHQ-1** black hole quencher-1

**PAGE** polyacrylamide gel electrophoresis

**PS** phosphorothioate

**L-BAPNA** N_α_-Benzoyl-L-arginine 4-nitroanilide hydrochloride

Materials

General:

The DNA concentrations were measured with a microvolume UV/Vis spectrophotometer (NanoDrop^TM^ One/One^C^, ThermoFisher Scientific). The annealing of DNA strands was performed in a thermocycler (Mastercycler Personal, Eppendorf). Polyacrylamide gel electrophoresis (PAGE) was performed in an 8x9 cm vertical mini-electrophoresis system (Mighty Small II, Hoefer) using an electrophoresis power supply (PowerPacbasic, BioRad) and imaged via a Gel imager (E-box, Vilber). Absorbance and fluorescence were measured in a plate reader using SpectraMax M3, Molecular Devices. The pH of the buffer solutions was adjusted using a pH meter (SD 305 pH/ORP, Lovibond^®^ Water Testing).

Chemicals:

ROTIPHORESE®NF-acrylamide/bis-solution 40 (29:1) and N,N,N',N'-Tetramethylethane-1,2-diamine (TEMED) were purchased from Carl Roth. Ammonium persulfate (APS), N_α_-Benzoyl-L-arginine 4-nitroanilide hydrochloride (L-BAPNA), boric acid, calcium chloride (CaCl_2_), disodium ethylenediaminetetraacetate dihydrate (EDTA-Na_2_ salt), magnesium chloride (MgCl_2_), potassium chloride (KCl), sodium chloride (NaCl), trypsin from bovine pancreas, and tris(hydroxymethyl)aminomethane (tris base) were purchased from Sigma-Aldrich (Germany). Exonuclease III, TriTrack DNA loading dye (6X), SYBR Gold Gel Stain (10000X), and Ultra Low Range DNA Ladder were purchased from Thermo Fisher Scientific (Germany), and T7 exonuclease was obtained from New England Biolabs Inc.

Buffers:

**TBE buffer:** 89 mM Tris, 89 mM boric acid, 20 mM EDTA

**Exo III buffer:** 25 mM Tris-HCl (pH 7.4 at 25 °C), 50 mM KCl, 20 mM MgCl_2_, 1 mM CaCl_2_

**T7 buffer:** 25 mM Tris-HCl (pH 8 at 25 °C), 20 mM KCl, 20 mM MgCl_2_, 1 mM CaCl_2_

**Exo III/T7 combi buffer:** 25 mM Tris-HCl (pH 8 at 25 °C), 50 mM KCl, 20 mM MgCl_2_, 1 mM CaCl_2_

Oligonucleotides:

Oligonucleotides used in this study were synthesized, labeled, and purified by biomers.net GmbH (Germany). The DNA strands were supplied as lyophilized powders and used without further purification. All strands were dissolved in sterile ultrapure water with a conductivity of less than 0.055 mS/cm. The oligonucleotides used in this work are summarized in Table S1.

Table S1. Oligonucleotides used in this work.

| **Oligonucleotide ID** | **Sequence 5’🡪 3’** | **nts** |
| --- | --- | --- |
| DNAPS (Exo III) | CCTCGCTCTGCTAATCCT*G*T*T*A | 22 |
| DNA fuel (toe-hold 0 nts) (Exo III) | TAACAGGATTAGCAGAGCGAGG | 22 |
| DNA fuel (toe-hold 1 nt) (Exo III) | TAACAGGATTAGCAGAGCGAGGT | 23 |
| DNA fuel (toe-hold 2 nts) (Exo III) | TAACAGGATTAGCAGAGCGAGGTT | 24 |
| DNA fuel (toe-hold 3 nts) (Exo III) | TAACAGGATTAGCAGAGCGAGGTTT | 25 |
| DNAPS (T7) | C*C*T*C*GCTCTGCTAATCCTGTTA | 22 |
| DNA fuel (toe-hold 0 nts) (T7) | TAACAGGATTAGCAGAGCGAGG | 22 |
| DNA fuel (toe-hold 5 nts) (T7) | TTTTTTAACAGGATTAGCAGAGCGAGG | 27 |
| DNA fuel (toe-hold 7 nts) (T7) | TTTTTTTTAACAGGATTAGCAGAGCGAGG | 29 |
| DNA fuel (toe-hold 10 nts) (T7) | TTTTTTTTTTTAACAGGATTAGCAGAGCGAGG | 32 |
| DNA fuel (toe-hold 12 nts) (T7) | TTTTTTTTTTTTTAACAGGATTAGCAGAGCGAGG | 34 |
| DNA fuel (toe-hold 15 nts) (T7) | TTTTTTTTTTTTTTTTAACAGGATTAGCAGAGCGAGG | 37 |
| DNA fuel (toe-hold 20 nts) (T7) | TTTTTTTTTTTTTTTTTTTTTAACAGGATTAGCAGAGCGAGG | 42 |
| DNAzyme A1 | TTTTTAACTTAATTTGCACCCATGTTA*C*T*C*T | 31 |
| DNAzyme A2 (toe-hold 0 nts) (Exo III) | GATATCAGCGATCAAATTAAGTTAAAAA | 28 |
| DNAzyme A2 (toe-hold 1 nt) (Exo III) | GATATCAGCGATCAAATTAAGTTAAAAAC | 29 |
| DNAzyme A2 (toe-hold 2 nts) (Exo III) | GATATCAGCGATCAAATTAAGTTAAAAACC | 30 |
| DNAzyme A2 (toe-hold 3 nts) (Exo III) | GATATCAGCGATCAAATTAAGTTAAAAACCC | 31 |
| DNAzyme reporter strand A | BHQ-1-AGAGTATrAGGA*6*A*T*C  6: dT-FAM | 15 |
| DNAzyme reporter strand A’ | BHQ-1-AGAGTATrAGGA*T*A*T*C-6-FAM | 15 |
| DNAzyme B1 | G*A*T*A*TCAGCGATCAAATTAAGAATTTTT | 28 |
| DNAzyme B2 (toe-hold 0 nts) (T7) | AAAAATTCTTAATTTGCACCCATGTTACTCT | 31 |
| DNAzyme B2 (toe-hold 5 nts) (T7) | TTTTTAAAAATTCTTAATTTGCACCCATGTTACTCT | 36 |
| DNAzyme B2 (toe-hold 7 nts) (T7) | TTTTTTTAAAAATTCTTAATTTGCACCCATGTTACTCT | 38 |
| DNAzyme B2 (toe-hold 10 nts) (T7) | TTTTTTTTTTAAAAATTCTTAATTTGCACCCATGTTACTCT | 41 |
| DNAzyme B2 (toe-hold 12 nts) (T7) | TTTTTTTTTTTTAAAAATTCTTAATTTGCACCCATGTTACTCT | 43 |
| DNAzyme reporter strand B | BHQ-1-A*G*A*G*TATrAGGATATC-6-FAM | 15 |
| Trypsin aptamer (Exo III) | CATGCTTCCCCAGGGAGATGGGTGTGAGGTTAGTTTGCGGGAACTAGAATTCCTGGCAAAGGGGGGCCTGGAGGAACATGCGTCGC*A*A*A*C | 90 |
| Trypsin fuel0-90 | GTTTGCGACGCATGTTCCTCCAGGCCCCCCTTTGCCAGGAATTCTAGTTCCCGCAAACTAACCTCACACCCATCTCCCTGGGGAAGCATG | 90 |
| Trypsin fuel0-30 (toe-hold 0 nts) (Exo III) | GTTTGCGACGCATGTTCCTCCAGGCCCCCC | 30 |
| Trypsin fuel10-40 | CATGTTCCTCCAGGCCCCCCTTTGCCAGGA | 30 |
| Trypsin fuel20-50 | CAGGCCCCCCTTTGCCAGGAATTCTAGTTC | 30 |
| Trypsin fuel30-60 | TTTGCCAGGAATTCTAGTTCCCGCAAACTA | 30 |
| Trypsin fuel40-70 | ATTCTAGTTCCCGCAAACTAACCTCACACC | 30 |
| Trypsin fuel50-80 | CCGCAAACTAACCTCACACCCATCTCCCTG | 30 |
| Trypsin fuel60-90 | ACCTCACACCCATCTCCCTGGGGAAGCATG | 30 |
| Trypsin fuel0-30 (toe-hold 1 nt) (Exo III) | GTTTGCGACGCATGTTCCTCCAGGCCCCCCA | 31 |
| Trypsin fuel0-30 (toe-hold 2 nts) (Exo III) | GTTTGCGACGCATGTTCCTCCAGGCCCCCCAA | 32 |
| Trypsin fuel0-30 (toe-hold 3 nts) (Exo III) | GTTTGCGACGCATGTTCCTCCAGGCCCCCCAAA | 33 |
| Trypsin aptamer (T7) | C*A*T*G*CTTCCCCAGGGAGATGGGTGTGAGGTTAGTTTGCGGGAACTAGAATTCCTGGCAAAGGGGGGCCTGGAGGAACATGCGTCGCAAAC | 90 |
| Trypsin fuel0-30 (toe-hold 0 nts) (T7) | GTTTGCGACGCATGTTCCTCCAGGCCCCCC | 30 |
| Trypsin fuel0-30 (toe-hold 5 nts) (T7) | TTTTTGTTTGCGACGCATGTTCCTCCAGGCCCCCC | 35 |
| Trypsin fuel0-30 (toe-hold 7 nts) (T7) | TTTTTTTGTTTGCGACGCATGTTCCTCCAGGCCCCCC | 37 |
| Trypsin fuel0-30 (toe-hold 10 nts) (T7) | TTTTTTTTTTGTTTGCGACGCATGTTCCTCCAGGCCCCCC | 40 |
| Trypsin fuel0-30 (toe-hold 12 nts) (T7) | TTTTTTTTTTTTGTTTGCGACGCATGTTCCTCCAGGCCCCCC | 42 |
| Trypsin aptamer (Apt) (T7/Exo III) | C*A*T*G*CTTCCCCAGGGAGATGGGTGTGAGGTTAGTTTGCGGGAACTAGAATTCCTGGCAAAGGGGGGCCTGGAGGAACATGCGTCGC*A*A*A*C | 90 |
| DNAzyme C1 | T*T*T*T*TAACTTAATTTGCACCCATGTTACTCTC*C*G*A*A | 36 |
| DNAzyme fuel C2 | A*A*G*C*CGATATCAGCGATCAAATTAAGTTAAAAA | 33 |
| DNAzyme D1 | T*T*T*T*TAACTTAATTTGCACCCATGTTA*C*T*C*T | 31 |
| DNAzyme fuel D2 | G*A*T*A*TCAGCGATCAAATTAAGTTAAAA A | 28 |
| DNAzyme reporter strand C (Rep C) | BHQ-1-A*G*A*G*TATrAGGA*6*A*T*C  6: dT-FAM | 15 |
| Inhibitor strand (Inhib) | A*G*A*G*TAT*rA*CGATATCGCCTGGAGGAACATGCGTCGCAAACTTTAAAA*A*A*A*A | 51 |
| Fuel_Try_ (toe 0) | T*T*T*T*TTTTAAAGTTTGCGACGCATGTTCCTCCAGGCCCCCC | 41 |
| Fuel_Try_ (toe 2) | T*T*T*T*TTTTAAAGTTTGCGACGCATGTTCCTCCAGGCCCCCCAA | 43 |
| Fuel_Try_ (toe 3) | T*T*T*T*TTTTAAAGTATTGCGACGCATGTTCCTCCAGGCCCCCCAAA | 44 |

* indicates a phosphorothioate bond (PS).

Supplemental Methods

Analysis of Exonuclease Dissipation Kinetics via Polyacrylamide Gel Electrophoresis

The complementary DNA strands (8 μM) were mixed in a stoichiometric ratio in the appropriate buffer and annealed. To determine the digestion kinetics of Exo III and T7, the hybridized DNA solution was incubated with Exo III (4 U µL^-1^) or T7 (0.4 U µL^-1^). 5 μL aliquots were taken at different time intervals and quenched with 15 μL of 1 M NaCl to stop digestion by the exonucleases. For band visualization, 5 μL of the quenched DNA solution was mixed with 15 μL of 1X TriTrack to achieve a final DNA concentration of 0.5 μM. 5 μL of this mixture was then loaded into the PAGE gel wells. Electrophoresis was performed at 110 V for 150 min in 1X TBE running buffer (89 mM Tris base, 89 mM boric acid, 2 mM EDTA-Na_2_). After staining with SYBR Gold for 20 min, the gels were imaged. To quantify digestion kinetics under different conditions, all lanes were analyzed using Fiji (ImageJ, National Institutes of Health, USA). The band intensities were integrated and normalized relative to the intensity before exonuclease addition. All experiments were performed at 25 °C and in triplicate.

PAGE-Exonuclease III buffer:

50 mM Tris-HCl (pH 7.4), 50 mM NaCl, 1 mM MgCl_2_

PAGE-T7 Exonuclease buffer:

50 mM Tris-HCl (pH 8.0), 20 mM KCl, 20 mM MgCl_2_

Standard procedure for the dissipative activation of dsDNAzyme A1/A2 or B1/B2 in the presence of Exo III or T7

The DNAzyme A1 or B1 (2.5 μM) was incubated in either a 25 mM Tris-HCl buffer (pH 7.4 at 25 °C) containing 50 mM KCl, 20 mM MgCl_2_ and 1 mM CaCl_2_ in the presence of Exo III or a 25 mM Tris-HCl buffer (pH 8 at 25 °C) containing 20 mM KCl, 20 mM MgCl_2_ and 1 mM CaCl_2_ for experiments conducted with T7. Then, Exo III or T7 with different concentrations and the corresponding DNAzyme reporter strand A or B (2.5 μM) were added to the mixture. The initial inactive state of the system was monitored via measuring the fluorescence intensity at 520 nm after excitation at 490 nm was measured every 30 s for 15 min. Subsequently, the DNA fuel (DNAzyme fuel A2 or B2) was added to initiate the dissipative activation of the dsDNAzyme, and the fluorescence intensity at 520 nm was again monitored every 30 s. After the system had returned to its initial state, another portion of DNA fuel was added to the system, and the next dissipative cycle was recorded. All experiments were conducted in triplicate using a black-walled 96 well-plate.

Fluorescence signals are reported in arbitrary units, and experiments were performed on different days using independently prepared plates. In addition, commercially obtained reporter strands, sometimes from different batches, were used. However, for any set of experiments intended for direct comparison, substrate from a single batch was employed. As a consequence, Δ fluorescence values are not directly comparable between figures and are interpreted only qualitatively. All quantitative analyses are therefore based on the transient lifetimes extracted from the timing of the signal rise and plateau/decay, rather than on the absolute Δ fluorescence values.

Standard procedure for the dissipative activation of bovine trypsin in the presence of Exo III or T7

Trypsin (0.1 μM) was deactivated by incubation for 30 min with trypsin aptamer (1 μM) in either a 25 mM Tris-HCl buffer (pH 7.4 at 25 °C), containing 50 mM KCl, 20 mM MgCl_2_ and 1 mM CaCl_2_ in the presence of Exo III or a 25 mM Tris-HCl buffer (pH 8 at 25 °C), containing 20 mM KCl, 20 mM MgCl_2_ and 1 mM CaCl_2_ for experiments conducted with T7. Subsequently, Exo III or T7 with different concentrations and L-BAPNA (100 µM; from a 10 mM stock solution in DMSO) were added to the mixture. The initial inactive state of the system was monitored by measuring the absorbance at 405 nm every 30 seconds for 15 min. Finally, the trypsin fuel was added to initiate the dissipative activation of trypsin, and the absorbance at 405 nm was again monitored every 30 s. After the absorbance reached a plateau, another portion of DNA fuel was added to the system, and the next dissipative activation cycle was recorded. All experiments were conducted in triplicate using a transparent 96 well-plate.

Standard procedure for the interlinked DNA system for dissipative control over biocatalysis

First, the inhibitor strand (5 µM) and the trypsin fuel strand (5 µM) were mixed in a 25 mM Tris-HCl buffer (pH 8 at 25 °C) containing 50 mM KCl, 20 mM MgCl2 and 1 mM CaCl2, and annealed by heating to 95 °C for 5 min in a thermocycler, before being cooled to 25 °C with a cooling rate of 0.4 °C min^-1^. After hybridization, L-BAPNA (250 µM; (from a 10 mM stock solution in DMSO) and DNAzyme reporter strand C (1, 2.5 or 5 µM) were added. In a separate vial, trypsin (0.025 µM) was incubated with the trypsin aptamer (0.25 µM) for 30 min, to ensure complete deactivation. After incubation, T7 (0.6 U µL^-1^), Exo III (0.025 U µL^-1^), and DNAzyme C1 (5 µM) were added. The two solutions were combined and added to a black-walled, clear bottom 96 well-plate, and the absorbance at 405 nm and the fluorescence at 520 nm after excitation at 490 nm were monitored every 2 min. After monitoring the system for 30 min appropriate amounts of DNAzyme fuel C2 (1, 2 or 4 eq.) were added and the absorbance at 405 nm and the fluorescence at 520 nm after excitation at 490 nm were once again monitored every 2 min.

Toe-hold-dependent DNA duplex digestion kinetics with Exo III and T7:

The complementary DNA strands (8 μM) were mixed in a stoichiometric ratio in either Exo III buffer or T7 buffer and annealed by heating to 95 °C for 5 min in a thermocycler before being cooled to 25 °C with a cooling rate of 0.4 °C min^-1^. To determine the digestion kinetics of Exo III, the hybridized DNA solution was subjected to Exo III (4 U µL^-1^) or T7 (0.4 U µL^-1^). 5 μL aliquots were taken at different time intervals and quenched with 15 μL NaCl (1 M) to stop the digestion of the DNA fuel by the different exonucleases. In order to visualize bands, 5 μL of the quenched DNA solution was mixed with 15 μL of TriTrack (1X). 5 μL of this mixture was pipetted into the PAGE gel wells. After electrophoresis, different lanes were quantified using ImageJ software.

PAGE:

20 % polyacrylamide gels were prepared in TBE buffer with 1 % APS (10 wt % sol.) as the initiator and 0.1 % TEMED as the catalyst. The electrophoresis was carried out at a voltage of 110 V for 120 min in TBE buffer.

Kinetic Modeling

Simulation and parameter estimation for the enzyme kinetics

Each enzyme reaction is either modelled with a Michaelis-Menten kinetic or approximated as first order kinetic.

The trypsin and DNAzyme reactions are not substrate limited in any of our experiments and described by first order kinetics. For the DNAzyme, we find that DNAzyme assembly is not a rate-limiting step. For trypsin, the amount of active enzyme as a function of the aptamer concentration is derived from experimental data and interpolated linearly. Digestion of hybridized fuel strands by Exo III is described by a Michaelis-Menten kinetic with a Michaelis constant obtained from literature^[1]^. For T7 a first order kinetic is applied as an approximation since the Michaelis constant was neither independently identifiable from the data nor available from literature. The exact equations can be found in the SI (Eqn. S1 – S7).

The reaction kinetics of all four systems (Trypsin/Exo III, Trypsin/T7, DNAzyme/Exo III, and DNAzyme/T7) are integrated in Python with SciPy’s solve_ivp^[2]^ and the BDF method. The catalytic constants for trypsin and the DNAzyme are obtained from linear fits to the initial reaction rate of the single addition experiments (Figure 2, 4, S13, S15, S36 and S38) and averaged. Similarly, the auto-hydrolysis rates or the different reporters are obtained from fits to the absorbance or fluorescence slopes that emerge after the fuel is depleted. Finally, the catalytic constants for Exo III and T7 are obtained by fitting the resulting model to experimental data for, both, single- and multi-addition experiments. For the fit, we apply SciPy’s minimize function with the Nealder-Mead method onto the mean squared error between the model and the experimental data.

DNAzyme system:

$$c_{hybridized fuel}=c_{\mathrm{DNAzyme}}=\left\{ \begin{aligned} c_{DNAzyme A1/B1}, &c_{\mathrm{fuel}}>c_{DNAzyme A1/B1} \\ c_{\mathrm{fuel}}, &c_{\mathrm{fuel}}\leq c_{DNAzyme A1/B1} \end{aligned} \right. (S1)$$

$$\frac{dI_{Flu.}}{dt}=k_{cat, DNAzyme}\cdot c_{\mathrm{DNAzyme}}+v_{min,reporter A/B} (S2)$$

Where $I_{Flu.}$ is the intensity of fluorescence, $c_{\mathrm{DNAzyme}}$ is the concentration of active DNAzyme, $c_{DNAzyme A1/B1}$ is the concentration of the PS-protected DNAzyme A1 or B1, $k_{cat, DNAzyme}$ is the catalytic constant of the DNAzyme, and $v_{min,reporter A/B}$ is the auto-hydrolysis rate (hydrolysis over time) of the DNAzyme reporter strand A or B.

With Exo III, fuel digestion is modeled as follows:

$$\frac{dc_{\mathrm{fuel}}}{dt}=-k_{cat,exo}\cdot c_{\mathrm{exo}}\cdot\frac{c_{hybridized fuel}}{K_{M,exo}+c_{hybridized fuel}} (S3)$$

Where $k_{cat,exo}$is the catalytic constant of Exo III, $c_{\mathrm{exo}}$ is the concentration of Exo III, and $K_{M,exo}$ is the Michaelis constant for Exo III.

With T7, fuel digestion is modeled as follows:

$$\frac{dc_{\mathrm{fuel}}}{dt}=-k_{cat,T7}\cdot c_{T7}\cdot c_{hybridized fuel} (S4)$$

Where $k_{cat,T7}$is the catalytic constant for T7, and $c_{T7}$ is the T7 concentration.

The enzyme kinetics for T7 were reduced to first-order kinetics. In the substrate-limited regime, this approximation introduces minimal error.

Trypsin system:

$$c_{hybridized fuel}=\left\{ \begin{aligned} c_{total aptamer}, &c_{\mathrm{fuel}}>c_{total aptamer} \\ c_{\mathrm{fuel}}, &c_{\mathrm{fuel}}\leq c_{total aptamer} \end{aligned} \right. (S5)$$

$$c_{active aptamer}=c_{total aptamer}-c_{hybridized fuel}(S6)$$

$$\frac{dA}{dt}=k_{cat, trypsin}\cdot c_{\mathrm{trypsin}}\cdot\mathrm{trypsin}_{\mathrm{activity}}\left( \frac{c_{active aptamer}}{c_{\mathrm{trypsin}}} \right)+v_{min,L-BAPNA} (S7)$$

Where $A$ is the absorbance at 405 nm of the cleaved L-BAPNA, $c_{hybridized fuel}$ is the concentration of hybridized fuel/aptamer duplex, $c_{\mathrm{fuel}}$ and $c_{total aptamer}$ are the total amounts of fuel and aptamer, $c_{\mathrm{trypsin}}$ is the trypsin concentration, trypsin_activity_ is a function that linearly interpolates between experimental values, $v_{min,L-BAPNA}$ is the auto-hydrolysis rate of the reporter molecule (L-BAPNA), and $k_{cat, trypsin}$ is the catalytic constant for trypsin.

Fuel digestion with Exo III or T7 is modeled identically to the DNAzyme system (see above).

Table S2. Simulated kinetic parameters

| **Parameter** | **Value** | **Unit** | **Comment** |
| --- | --- | --- | --- |
| $k_{cat, DNAzyme}$ | 1.5 | a.u./min/µM |  |
| $v_{min,reporter A/B}$ | 0.32 | a.u./min |  |
| $k_{cat,exo}$ | 34.5 | 1/min | For the trypsin system. |
| $k_{cat,exo}$ | 5.87 | 1/min | For the DNAzyme system. |
| $K_{M,exo}$ | 0.12 | µM |  |
| $k_{cat,T7}$ | 0.14 | µM/U/min | For the trypsin system. |
| $k_{cat,T7}$ | 0.087 | µM/U/min | For the DNAzyme system. |
| $k_{cat, trypsin}$ | 0.01167 | a.u./min/µM |  |
| $v_{min,L-BAPNA}$ | 0.0000254 | a.u./min |  |

The differences in the values of $k_{cat,exo}$ and $k_{cat,T7}$ obtained for trypsin and DNAzyme systems can potentially be ascribed to the different reaction buffer conditions used for the study.

Waste accumulation was not included in the model, as doing so would have required assumptions about waste kinetics and downstream effects that could not be reliably constrained by the available data. To avoid overfitting and overinterpretation, a simplified modeling approach was adopted.

Supplementary Figures and Tables


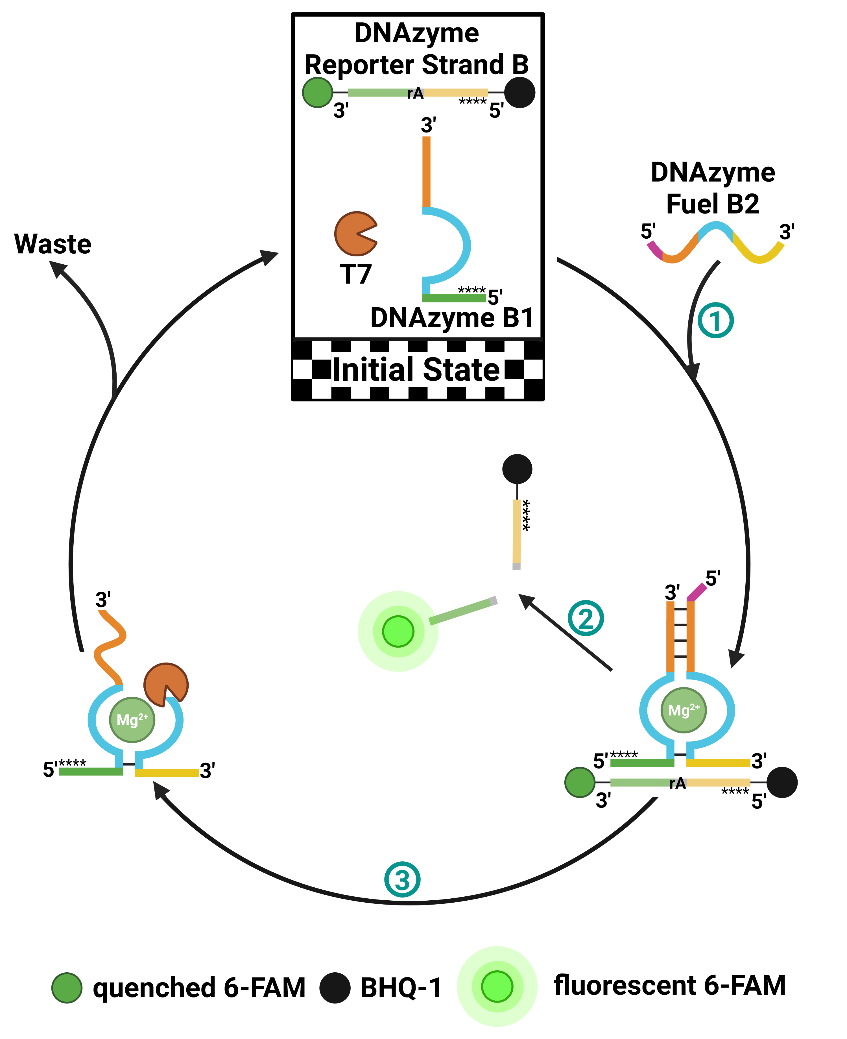


**Figure S1.** Schematic representation of T7-governed dissipative regulation of the dsDNAzyme B1/B2. **Step 1:** Addition of DNAzyme fuel B2, leading to the formation of the active dsDNAzyme B1/B2. **Step 2:** Cleavage of the DNAzyme reporter strand B by the active dsDNAzyme B1/B2. **Step 3:** Dissipation of DNAzyme fuel B2 via T7 digestion. Four consecutive PS bonds at the 5′-end protect DNAzyme B1 and the DNAzyme reporter strand B from T7 digestion (indicated by four black stars). Created by Biorender.com


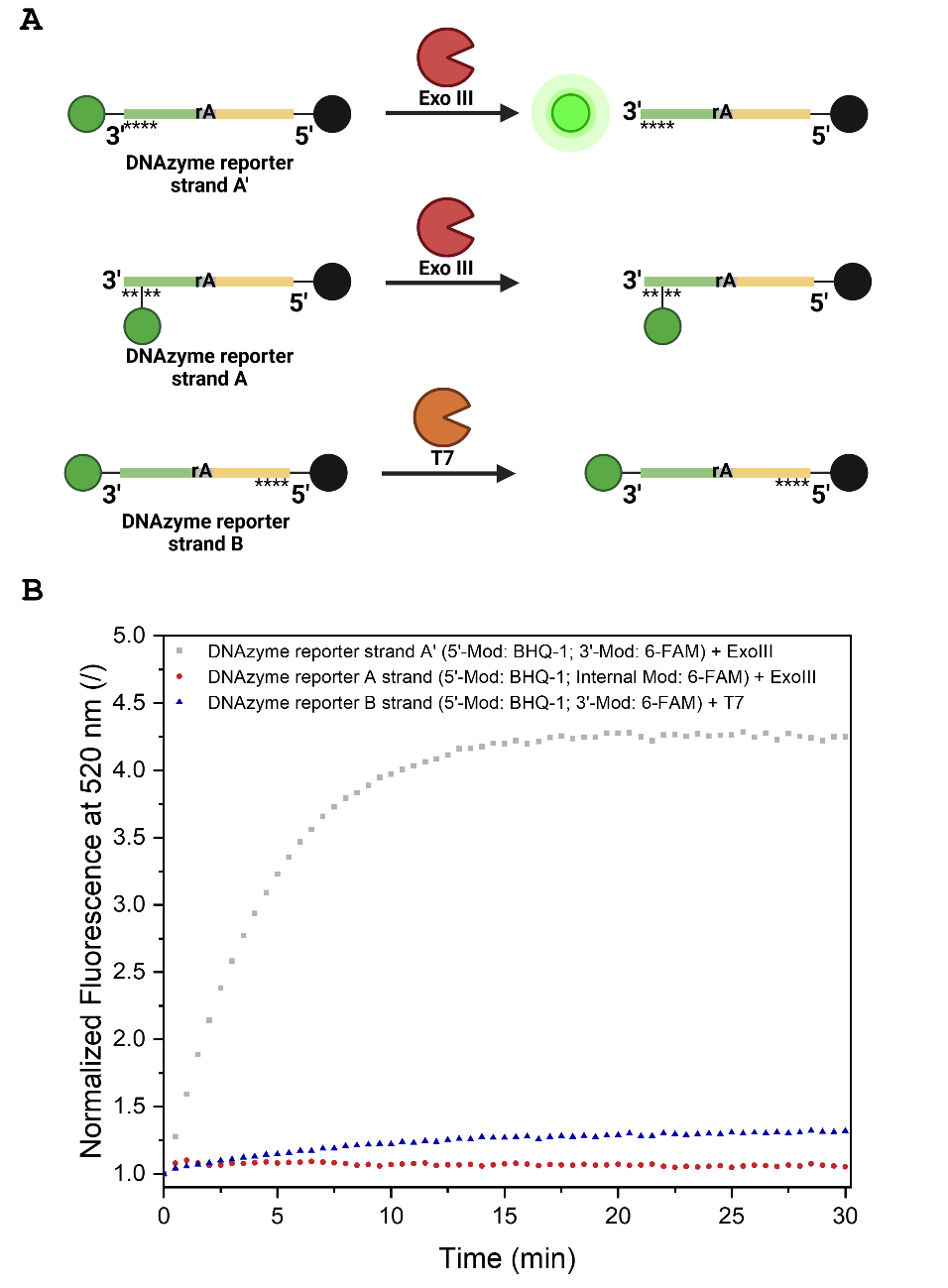


**Figure S2.** Stability of DNAzyme reporter strand modifications in the presence of Exo III or T7. (A) Schematic representation showing the influence of Exo III and T7 on the stability of DNAzyme reporter strands modified at terminal and internal positions. (B) Normalized fluorescence intensity at 520 nm over time following excitation at 490 nm of different DNAzyme reporter strands to assess their stability in the presence of Exo III and T7. Gray: DNAzyme reporter strand A’ (5 µM) with a 6-FAM modification at the 3’-end and a BHQ-1 modification at the 5’-end in the presence of Exo III (2 U µL^-1^). Red: DNAzyme reporter strand A (5 µM) with an internal 6-FAM modification and a BHQ-1 modification at the 5’-end in the presence of Exo III (2 U µL^-1^). Blue: DNAzyme reporter strand B (5 µM) with a 6-FAM modification at the 3’-end and a BHQ-1 modification at the 5’-end in the presence of T7 (0.8 U µL^-1^). Panel A created by Biorender.com


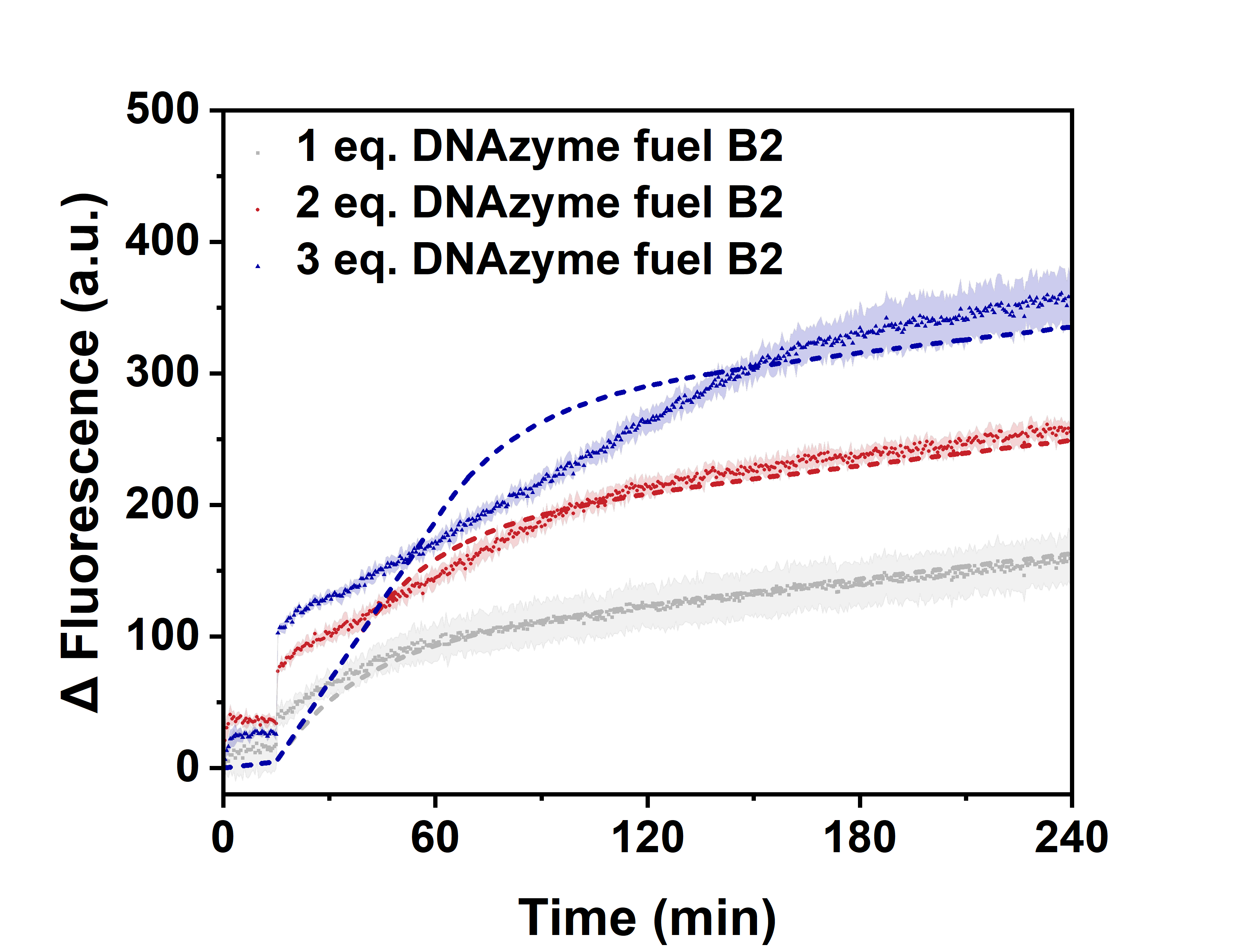


**Figure S3. Dissipative control over dsDNAzyme B1/B2 activity in the presence of T7 and varying amounts of DNAzyme fuel B2.** Δ Fluorescence intensity at 520 nm, after excitation at 490 nm, plotted against time in the presence of 1, 2, or 3 eq. of DNAzyme fuel B2, DNAzyme reporter strand B (2.5 µM), and T7 (0.5 U µL^-1^). Data are presented as mean ± standard deviation (SD) of three independent experiments (n = 3); error bars represent the SD.


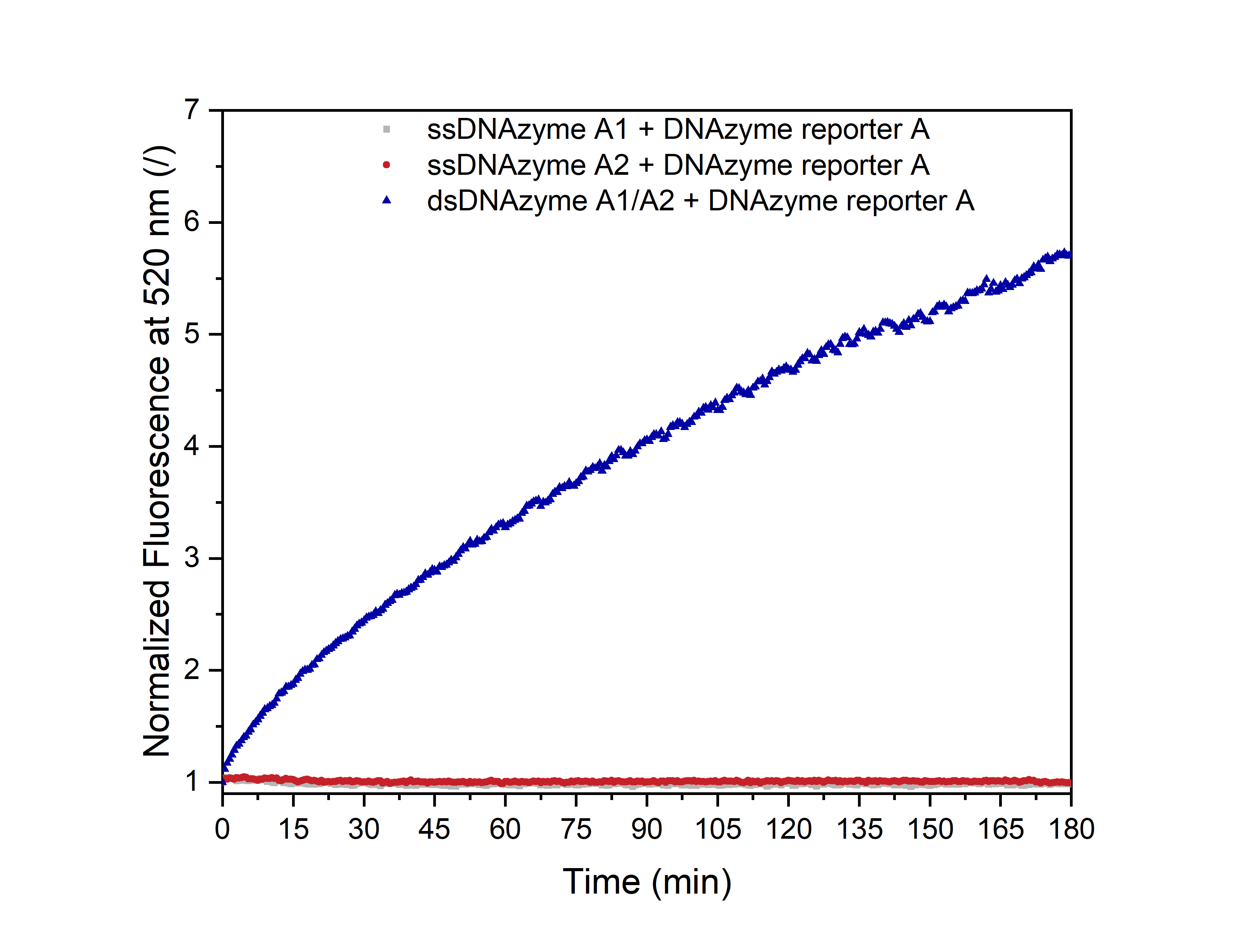
**Figure S4.** Normalized fluorescence intensity at 520 nm over time after excitation at 490 nm for ssDNAzyme A1, ssDNAzyme A2, and dsDNAzyme A1/A2 under non-dissipative conditions. Gray: ssDNAzyme A1 (2.5 µM) with DNAzyme reporter strand A (2.5 µM). Red: ssDNAzyme A2 (2.5 µM) with DNAzyme reporter strand A (2.5 µM). Blue: dsDNAzyme A1/A2 (2.5 µM) with DNAzyme reporter strand A (2.5 µM).**
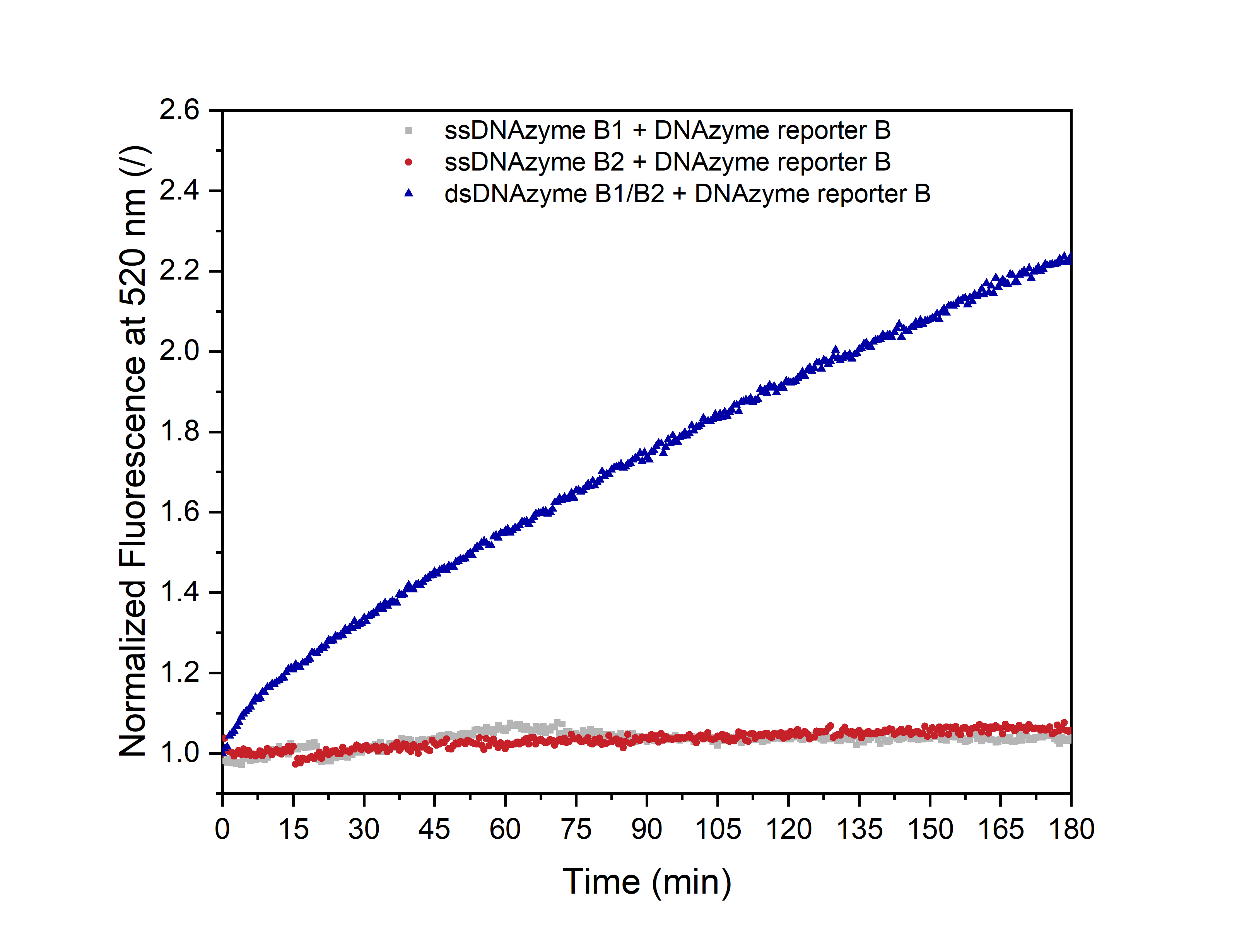
Figure S5.** Normalized fluorescence intensity at 520 nm over time after excitation at 490 nm for ssDNAzyme B1, ssDNAzyme B2, and dsDNAzyme B1/B2 under non-dissipative conditions. Gray: ssDNAzyme B1 (2.5 µM) with DNAzyme reporter strand B (2.5 µM). Red: ssDNAzyme B2 (2.5 µM) with DNAzyme reporter strand B (2.5 µM). Blue: dsDNAzyme B1/B2 (2.5 µM) with DNAzyme reporter strand B (2.5 µM).


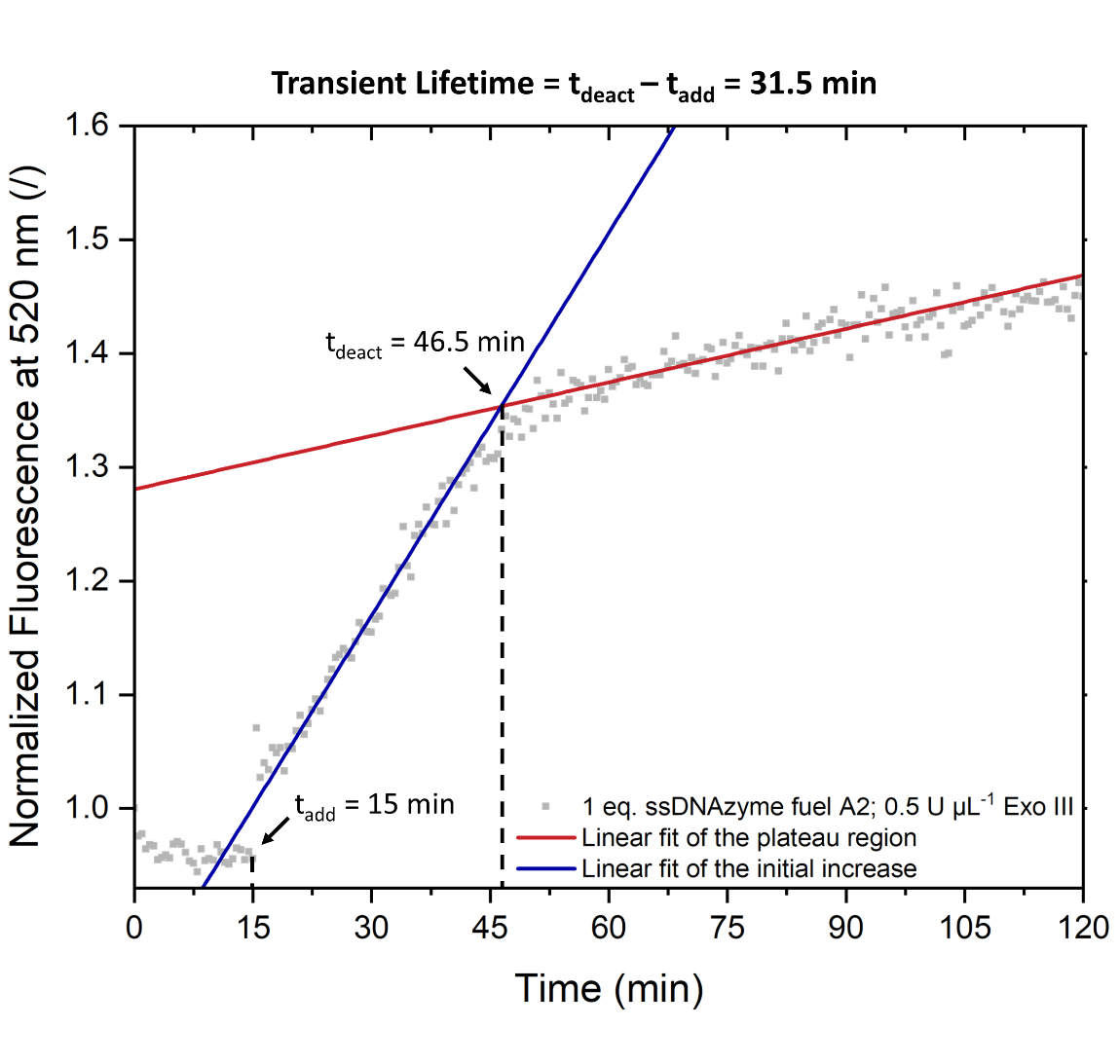
**Figure S6.** Graphical determination of the transient lifetime exemplified with the Exo III-regulated dsDNAzyme A1/A2 system. Gray: Normalized fluorescence intensity at 520 nm of dsDNAzyme A1/A2 (2.5 µM) activity in the presence of Exo III (0.5 U µL^-1^) plotted against time after excitation at 490 nm. Red: Linear fit of the plateau region of the curve. Blue: Linear fit of the initial increase of the curve. The fits were performed using the OriginPro software (2024b).


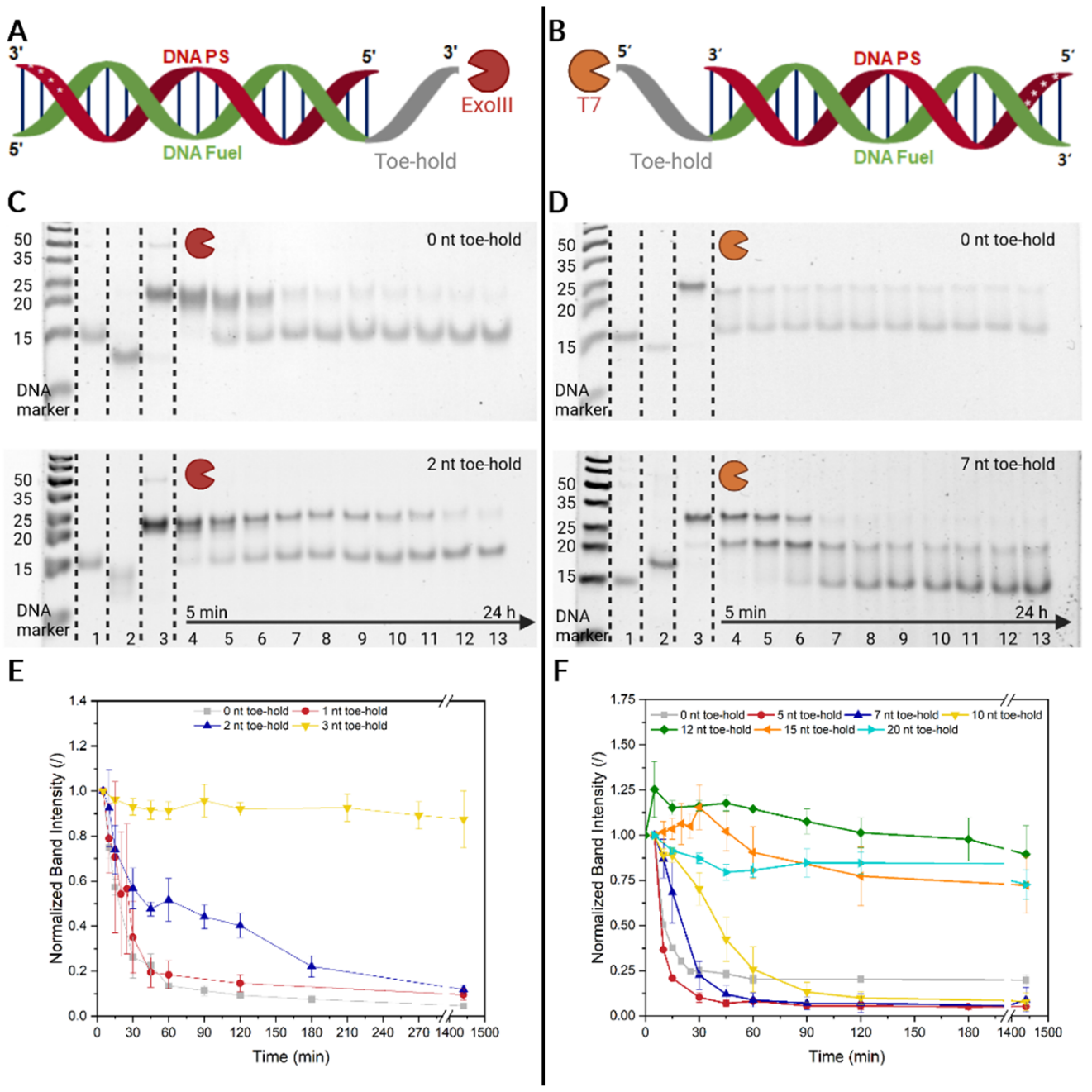


**Figure S7.** Design and evaluation of toe-hold-dependent DNA duplex digestion by Exo III or T7. **(A)** Schematic representation of the DNA sequence design for evaluating toe-hold dependent digestion kinetics with Exo III. **(B)** Schematic representation of the DNA sequence design for evaluating the toe-hold-dependent digestion kinetics with T7. In both cases, the DNA duplex contains a single-stranded DNA fuel (green) with a toe-hold sequence at the 3’-end for Exo III and at the 5’-end for T7. The toe-hold lengths vary as follows: 0, 1, 2, and 3 nts for Exo III, and 0, 5, 7, 10, 12, 15, and 20 nts for T7. A 22-mer DNA PS (red) sequence is complementary to the DNA fuel. DNA PS is modified with four consecutive phosphorothioate bonds at the 3’-end for use with Exo III or at the 5’-end for use with T7 (marked as white stars), providing protection from exonuclease digestion. **(C)** Time-dependent PAGE analysis of DNA duplexes (8 μM) with 0 nt (top panel) or 2 nt (bottom panel) toe-holds, in the presence of Exo III (4 U μL^-1^). Lane 1: DNA PS; Lane 2: DNA fuel; Lane 3: DNA duplex formed by DNA PS and DNA fuel; Lane 4-13: Aliquots taken after 5 min, 10 min, 15 min, 20 min, 25 min, 30 min, 45 min, 60 min, 120 min, and 24 h of Exo III incubation with the DNA duplex containing a 0 nt toe-hold, and after 5 min, 10 min, 15 min, 30 min, 45 min, 60 min, 90 min, 120 min, 180 min, and 24 h of incubation of Exo III with DNA duplex containing a 2 nt toe-hold. **(D)** Time-dependent PAGE analysis of DNA duplexes (8 μM) containing 0 nt (top panel) or 7 nt (bottom panel) toe-holds, in the presence of T7 (0.4 U μL^-1^). Lane 1: DNA PS; Lane 2: DNA fuel; Lane 3: DNA duplex formed by DNA PS and DNA fuel; Lane 4-13: Aliquots taken after 5 min, 10 min, 15 min, 20 min, 25 min, 30 min, 45 min, 60 min, 120 min, and 24 h of T7 incubation with the DNA duplex containing a 0 nt toe-hold, and after 5 min, 10 min, 15 min, 30 min, 45 min, 60 min, 90 min, 120 min, 180 min, and 24 h of incubation of T7 with the DNA duplex containing a 7 nt toe-hold. **(E)** Normalized band intensity plotted as a function of time for DNA duplexes containing 0, 1, 2, or 3 thymine units at the 3’-end of the DNA fuel in the presence of Exo III. **(F)** Normalized band intensity plotted as a function of time for DNA duplexes containing 0, 5, 7, 10, 12, 15, or 20 thymine units at the 5’-end of the DNA fuel in the presence of T7. Data are presented as mean ± standard deviation (SD) of three independent experiments (n = 3); error bars represent the SD.


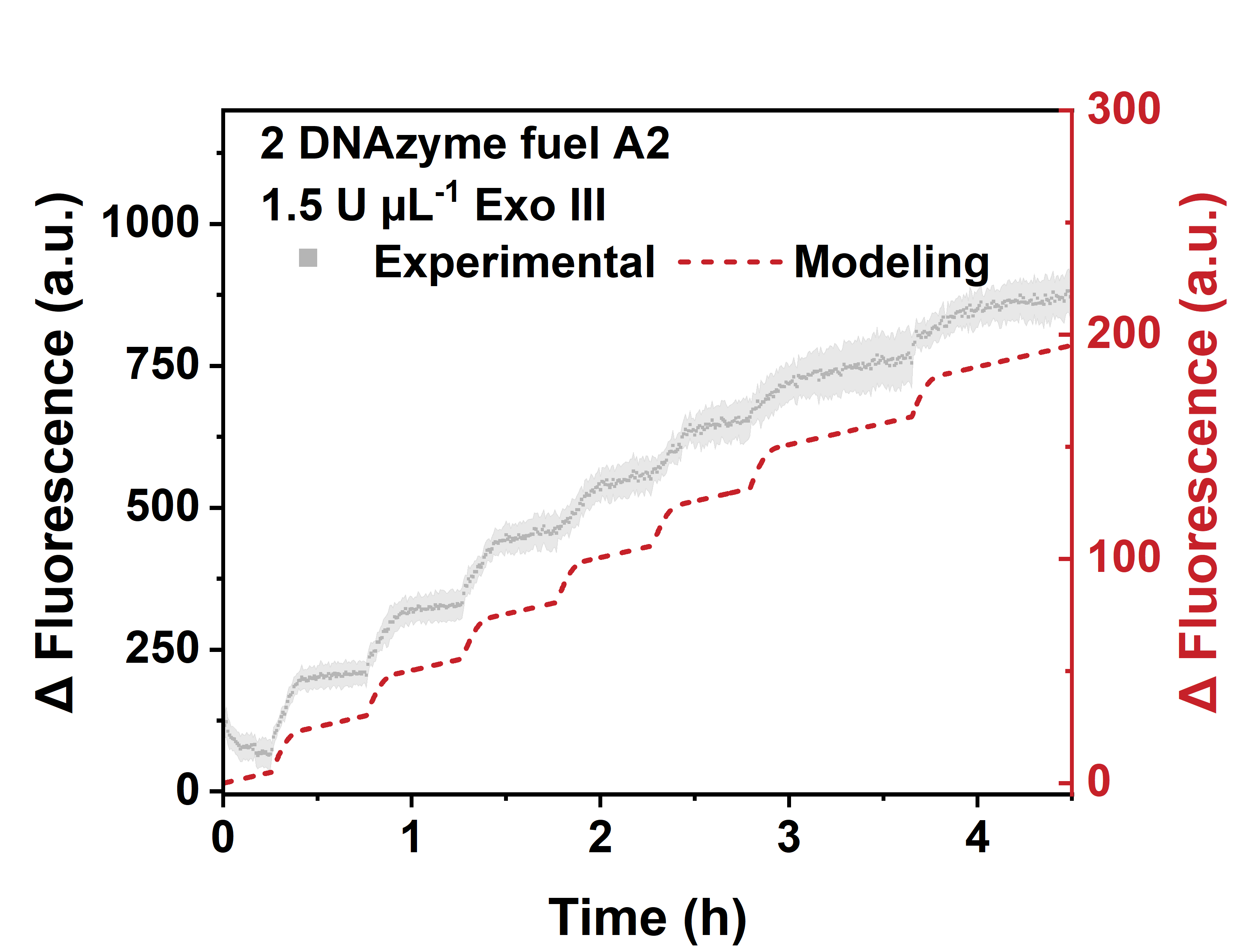


Figure S8. Successive additions of the ssDNAzyme fuel A2 in the presence of 1.5 U µL^-1^ Exo III. Fluorescence intensity at 520 nm was monitored for 15 min before the addition of ssDNAzyme fuel A2 (1 eq.) following excitation at 490 nm. Consecutive additions of ssDNAzyme fuel A2 (1 eq.) were performed at 15, 45.5, 76, 106, 137, 167.5, and 219 min in the presence of the DNAzyme reporter strand A (2.5 µM) and Exo III (1.5 U µL^-1^). Grey: Δ fluorescence intensity at 520 nm after excitation at 490 nm (left y-axis) of the dsDNAzyme A1/A2 (2.5 µM) activity in the presence of Exo III (1.5 U µL^-1^), plotted against the time (experimental data). Red: Modeled Δ fluorescence intensity (right y-axis) plotted against the time for dsDNAzyme A1/A2 (2.5 µM) activity in the presence of Exo III (1.5 U µL^-1^). The Δ fluorescence intensity of the modeled data was rescaled due to the higher starting fluorescence in the successive addition experiment compared to the single experiments used for determining the kinetic parameters for the model. Data are presented as mean ± standard deviation (SD) of three independent experiments (n = 3); error bars represent the SD.


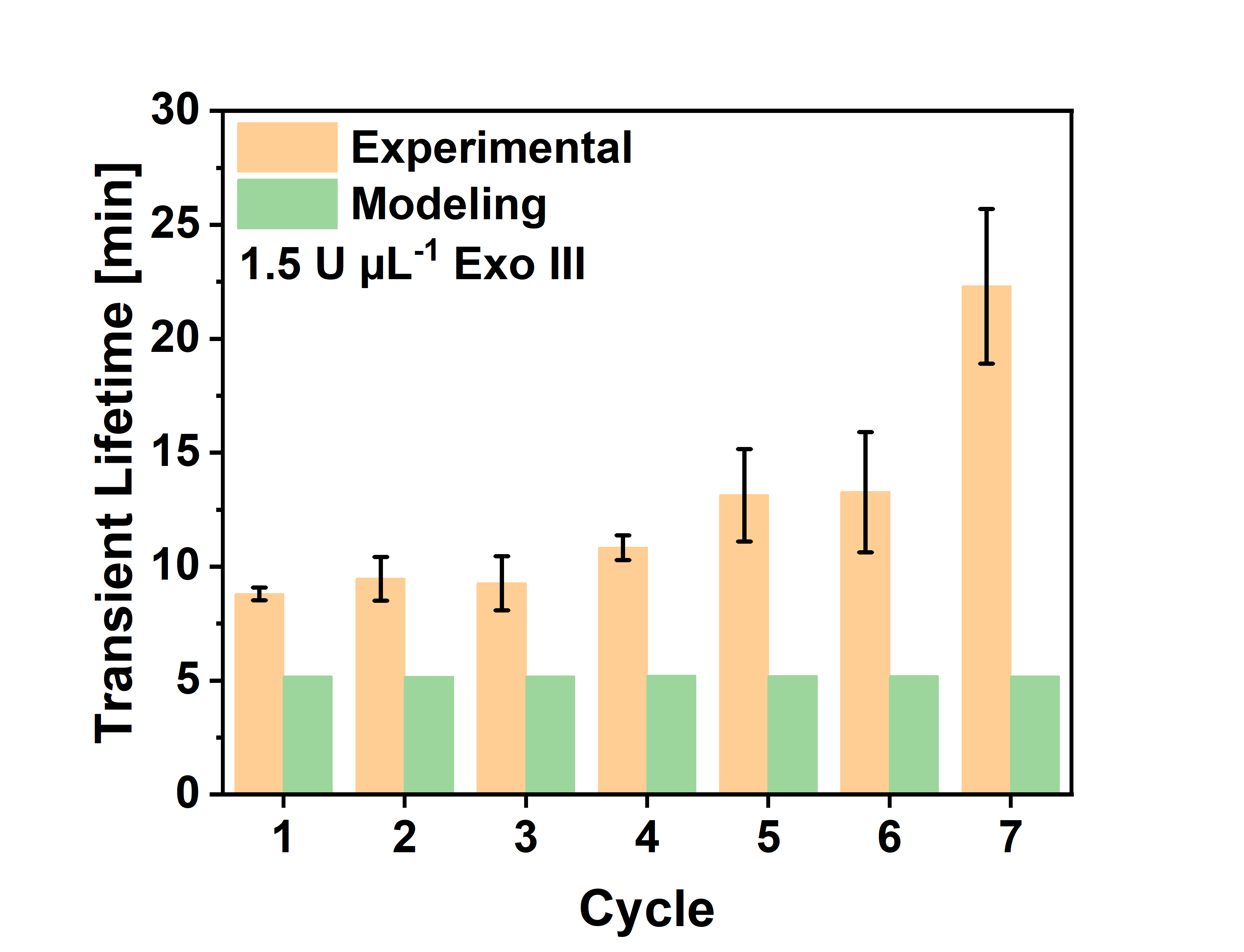


Figure S9. Transient lifetime of dsDNAzyme A1/A2 in the presence of 1.5 U µL^-1^ Exo III as a function of consecutive fuel additions. Transient lifetimes of dsDNAzyme A1/A2 (2.5 µM) in the presence of Exo III (1.5 U µL^-1^) determined from the experimental data (yellow) and the predicted transient lifetimes (green) derived from the kinetic model. Data are presented as mean ± standard deviation (SD) of three independent experiments (n = 3); error bars represent the SD.

Table S3. Statistical significance determined by analysis of variance (ANOVA) using the Origin 2024b software for the successive additions of the ssDNAzyme fuel A2 in the presence of 1.5 U µL^-1^ Exo III.

| Cycle Nr. | 1 | 2 | 3 | 4 | 5 | 6 | 7 |
| --- | --- | --- | --- | --- | --- | --- | --- |
| 1 |  | n.s. | n.s. | n.s. | n.s. | n.s. | *** |
| 2 | n.s. |  | n.s. | n.s. | n.s. | n.s. | *** |
| 3 | n.s. | n.s. |  | n.s. | n.s. | n.s. | *** |
| 4 | n.s. | n.s. | n.s. |  | n.s. | n.s. | *** |
| 5 | n.s. | n.s. | n.s. | n.s. |  | n.s. | ** |
| 6 | n.s. | n.s. | n.s. | n.s. | n.s. |  | ** |
| 7 | *** | *** | *** | *** | ** | ** |  |

Significance: n.s. = not significant; * = p < 0.05; ** = p < 0.01; *** = p < 0.001.


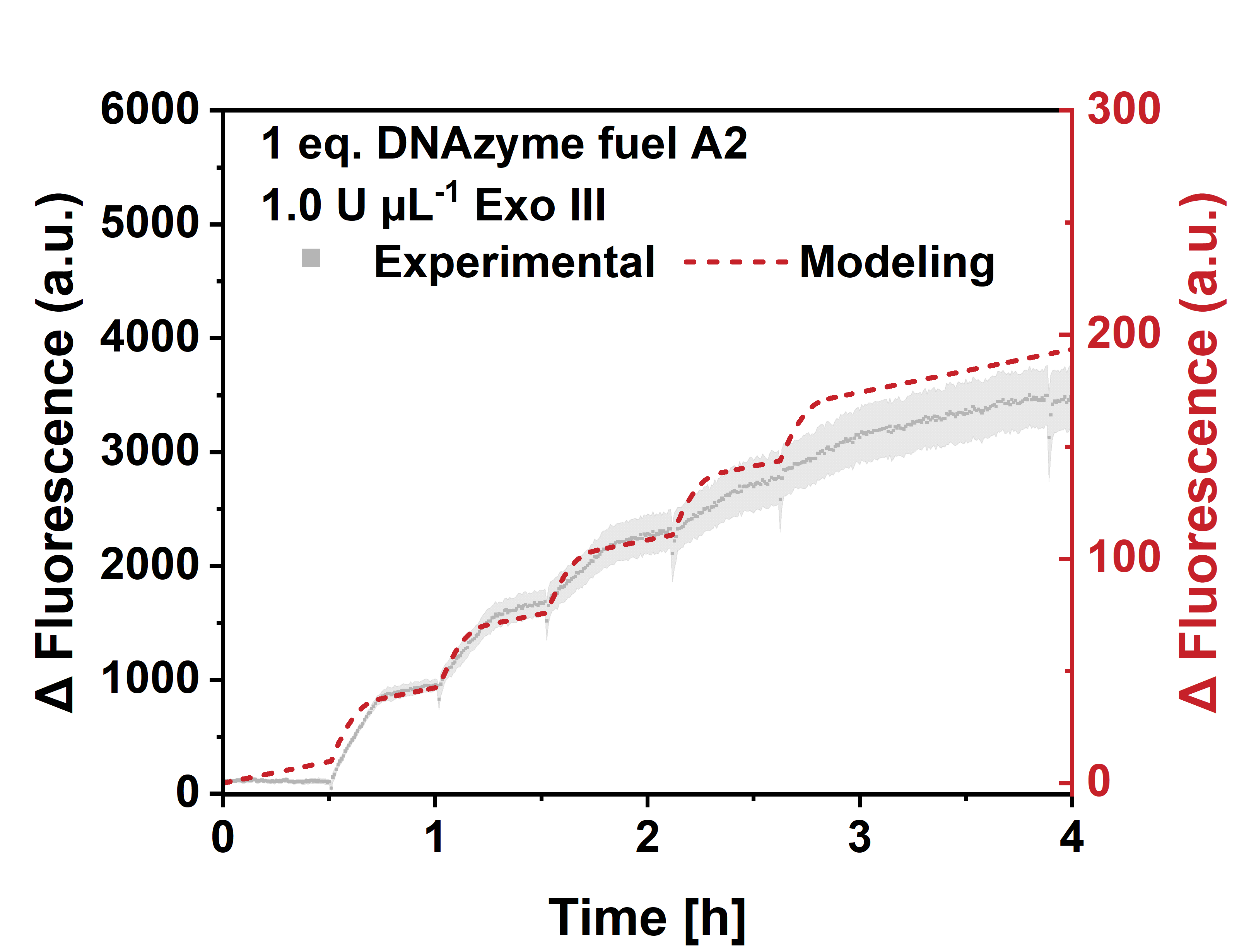
Figure S10. Successive additions of ssDNAzyme fuel A2 in the presence of 1.0 U µL^-1^ Exo III. Fluorescence intensity at 520 nm was monitored for 30 min before the addition of ssDNAzyme fuel A2 (1 eq.) after excitation at 490 nm. Consecutive additions of ssDNAzyme fuel A2 (1 eq.) were performed at 30, 61.5, 91.5, 127.5, and 158 min in the presence of DNAzyme reporter strand A (2.5 µM) and Exo III (1.0 U µL^-1^). Grey: Δ fluorescence intensity at 520 nm after excitation at 490 nm (left y-axis) of the dsDNAzyme A1/A2 (2.5 µM) activity in the presence of Exo III (1.0 U µL^-1^), plotted against time (experimental data). Red: Modeled Δ fluorescence intensity (right y-axis) plotted against time for dsDNAzyme A1/A2 (2.5 µM) activity in the presence of Exo III (1.0 U µL^-1^). The Δ fluorescence intensity of the modeled data was rescaled due to a higher starting fluorescence in the successive addition experiment compared to the single experiments used to determine the kinetic parameters for the model. Data are presented as mean ± standard deviation (SD) of three independent experiments (n = 3); error bars represent the SD.


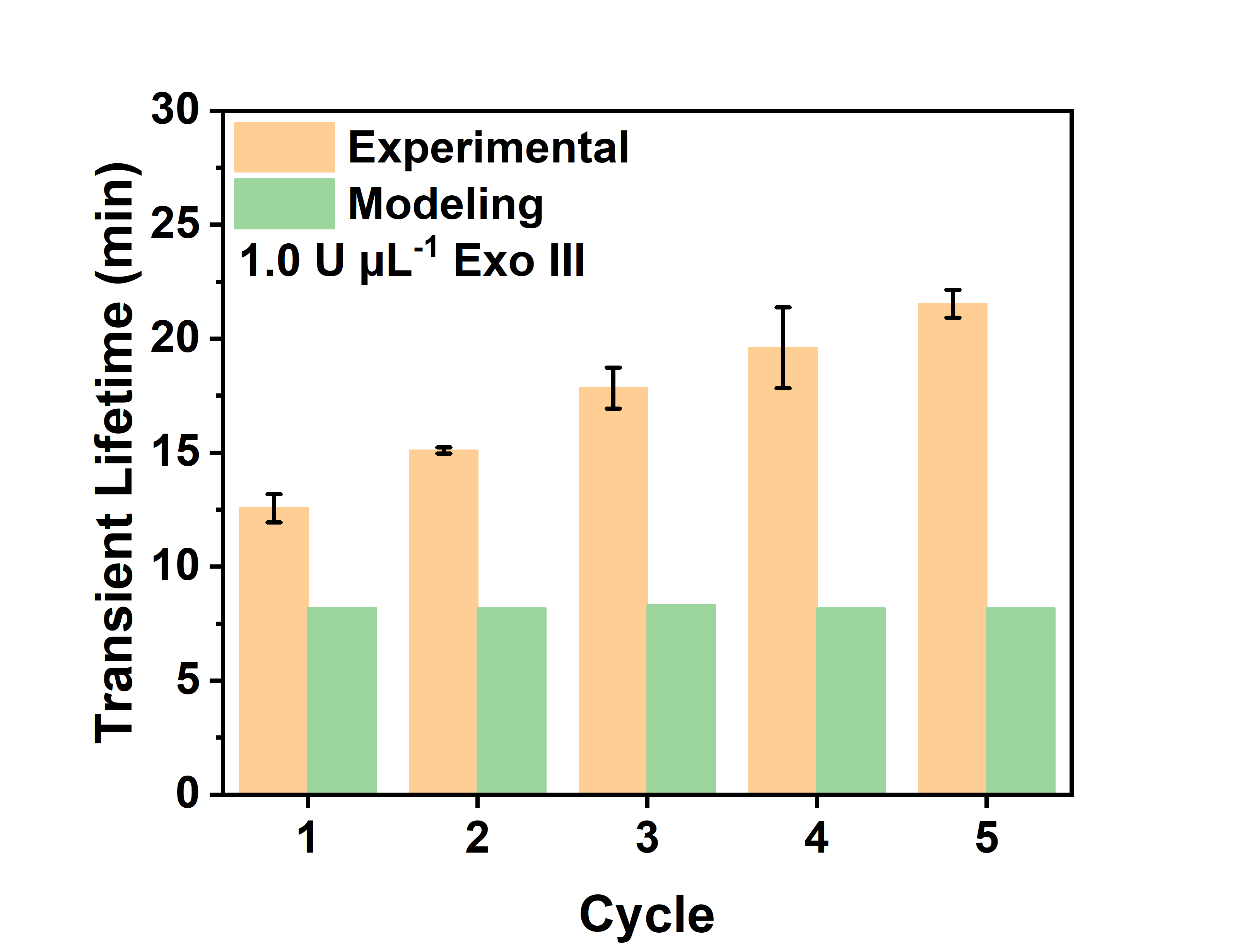


**Figure S11**. Transient lifetime of dsDNAzyme A1/A2 in the presence of 1.0 U µL^-1^ Exo III as a function of consecutive fuel additions. Transient lifetimes of dsDNAzyme A1/A2 (2.5 µM) in the presence of Exo III (1.0 U µL^-1^) determined from the experimental data (yellow) and predicted transient lifetimes (green) from the kinetic model. Data are presented as mean ± standard deviation (SD) of three independent experiments (n = 3); error bars represent the SD.

Table S4. Statistical significance determined by analysis of variance (ANOVA) using the Origin 2024b software for the successive additions of the ssDNAzyme fuel A2 in the presence of 1.0 U µL^-1^ Exo III.

| Cycle Nr. | 1 | 2 | 3 | 4 | 5 |
| --- | --- | --- | --- | --- | --- |
| 1 |  | n.s. | ** | *** | *** |
| 2 | n.s. |  | n.s. | ** | ***. |
| 3 | ** | n.s. |  | n.s. | * |
| 4 | *** | ** | n.s. |  | n.s. |
| 5 | *** | *** | * | n.s. |  |

Significance: n.s. = not significant; * = p < 0.05; ** = p < 0.01; *** = p < 0.001.


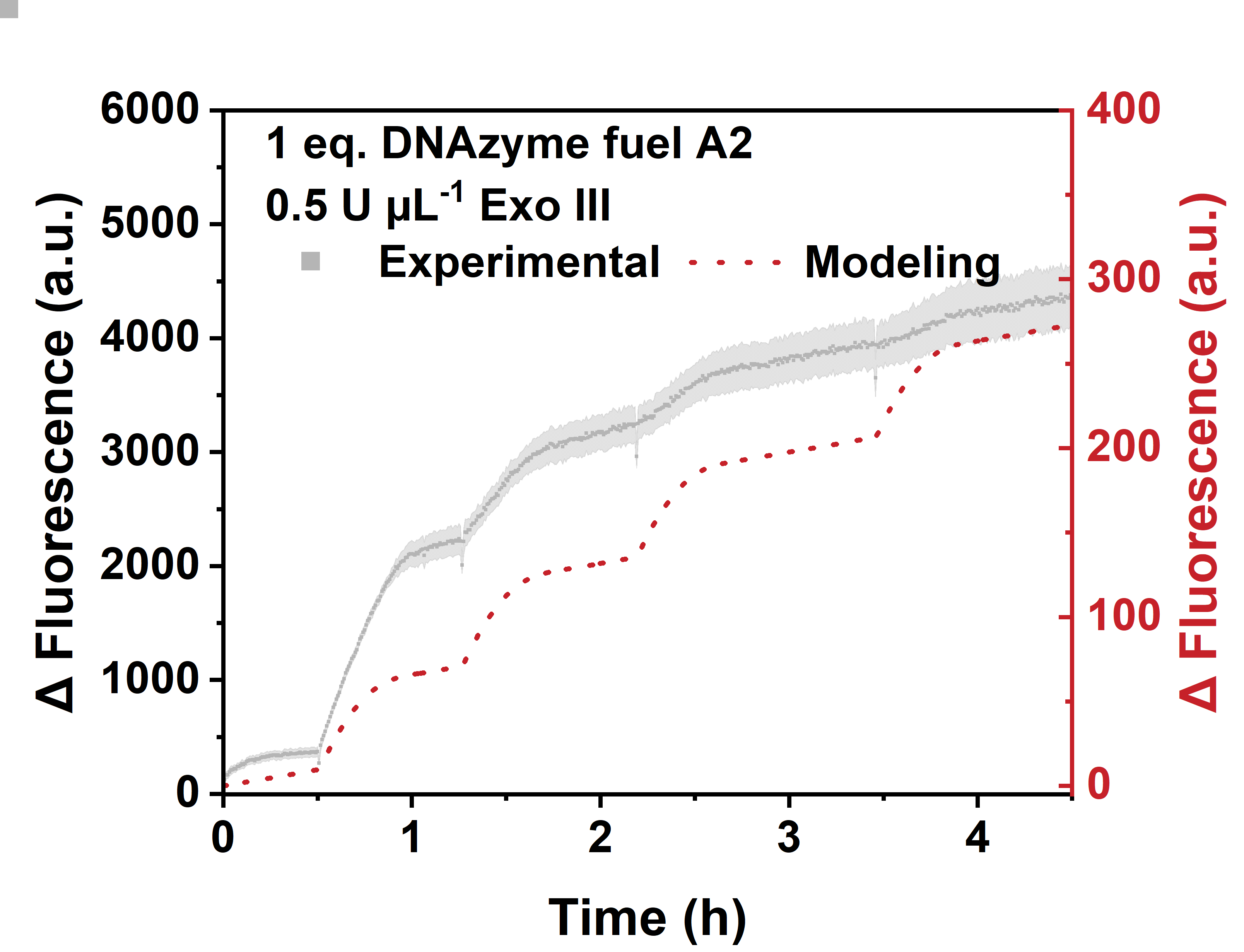
 Figure S12. Successive additions of ssDNAzyme fuel A2 in the presence of 0.5 U µL^-1^ Exo III. Fluorescence intensity at 520 nm was monitored for 30 min before the addition of ssDNAzyme fuel A2 (1 eq.) after excitation at 490 nm. Consecutive additions of ssDNAzyme fuel A2 (1 eq.) were performed at 30, 76.5, 131.5, and 207.5 min in the presence of DNAzyme reporter strand A (2.5 µM) and Exo III (0.5 U µL^-1^). Grey: Δ fluorescence intensity at 520 nm after excitation at 490 nm (left y-axis) of the dsDNAzyme A1/A2 (2.5 µM) activity in the presence of Exo III (0.5 U µL^-1^) plotted against time (experimental data). Red: Modeled Δ fluorescence intensity (right y-axis) plotted against time of dsDNAzyme A1/A2 (2.5 µM) activity in the presence of Exo III (0.5 U µL^-1^). The Δ fluorescence intensity of the modeled data was rescaled due to the higher starting fluorescence in the successive-addition experiment compared to the single-addition experiments used to determine the kinetic parameters. In the experimental traces, successive fuel additions lead to a clear decrease in Δ fluorescence, with the first addition producing the largest response. This trend can plausibly be attributed to progressive depletion of the fluorescent reporter, which is irreversibly cleaved and not replenished during the experiment. In our kinetic model, reporter depletion is not explicitly included; instead, the fluorescence signal is treated as a proxy for the concentration of activated DNAzyme, and the reporter is assumed to remain effectively in excess. As a result, the simulations capture the transient lifetimes and overall activation-deactivation kinetics but do not reproduce the experimentally observed reduction in step height with successive fuel additions. We note that reporter depletion primarily affects Δ fluorescence and is expected to have only a minor influence on the extracted transient lifetimes, which are determined from the timing of the rise and plateau. Data are presented as mean ± standard deviation (SD) of three independent experiments (n = 3); error bars represent the SD.


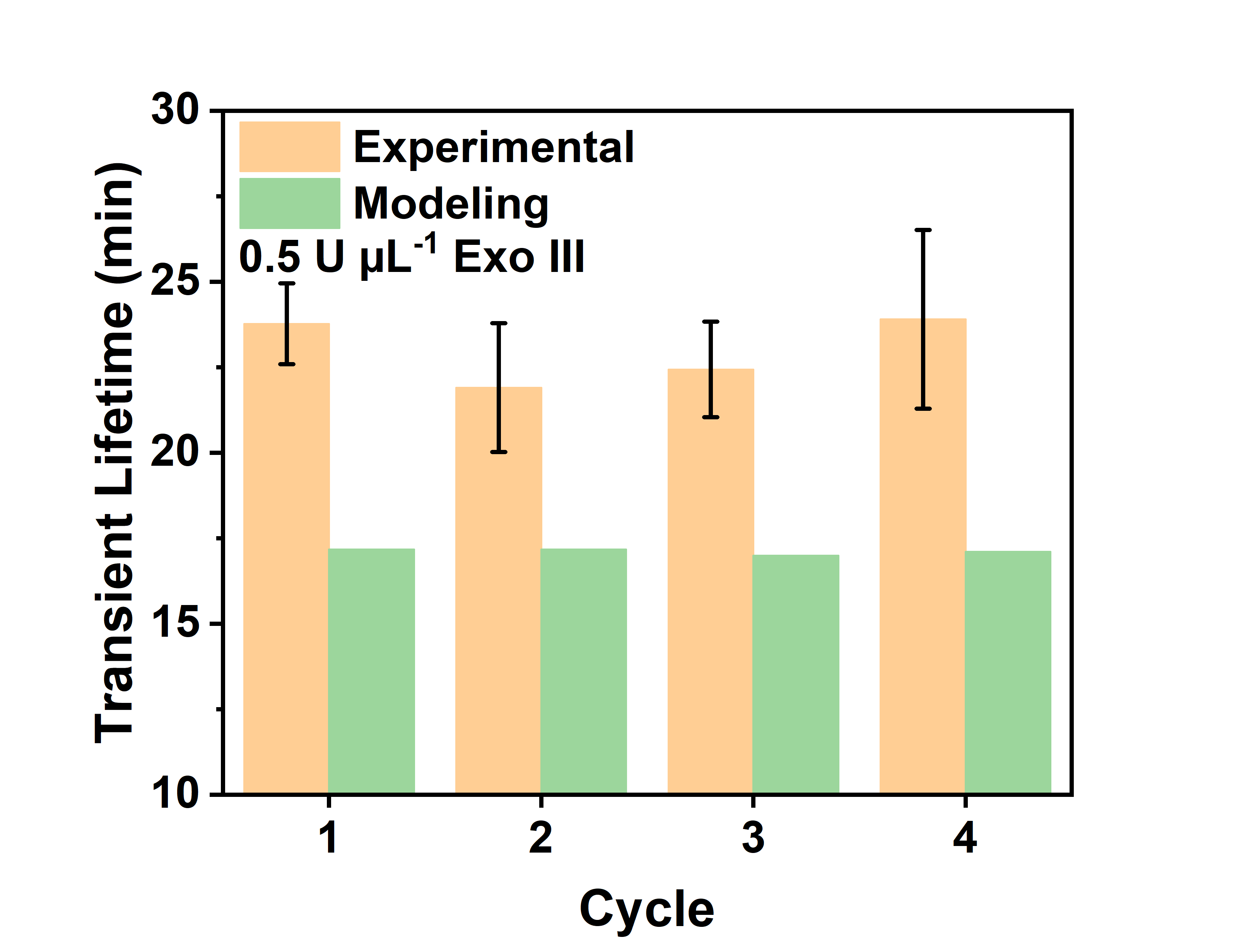


Figure S13. Transient lifetime of dsDNAzyme A1/A2 in the presence of 0.5 U µL^-1^ Exo III as a function of consecutive fuel additions. Transient lifetimes of dsDNAzyme A1/A2 (2.5 µM) in the presence of Exo III (0.5 U µL^-1^) determined from the experimental data (yellow) and predicted transient lifetimes (green) determined from the kinetic model. Data are presented as mean ± standard deviation (SD) of three independent experiments (n = 3); error bars represent the SD.

Table S5. Statistical significance determined by analysis of variance (ANOVA) using the Origin 2024b software for the successive additions of the ssDNAzyme fuel A2 in the presence of 0.5 U µL^-1^ Exo III.

| Cycle Nr. | 1 | 2 | 3 | 4 |
| --- | --- | --- | --- | --- |
| 1 |  | n.s. | n.s. | n.s. |
| 2 | n.s. |  | n.s. | n.s. |
| 3 | n.s. | n.s. |  | n.s. |
| 4 | n.s. | n.s. | n.s. |  |

Significance: n.s. = not significant; * = p < 0.05; ** = p < 0.01; *** = p < 0.001.


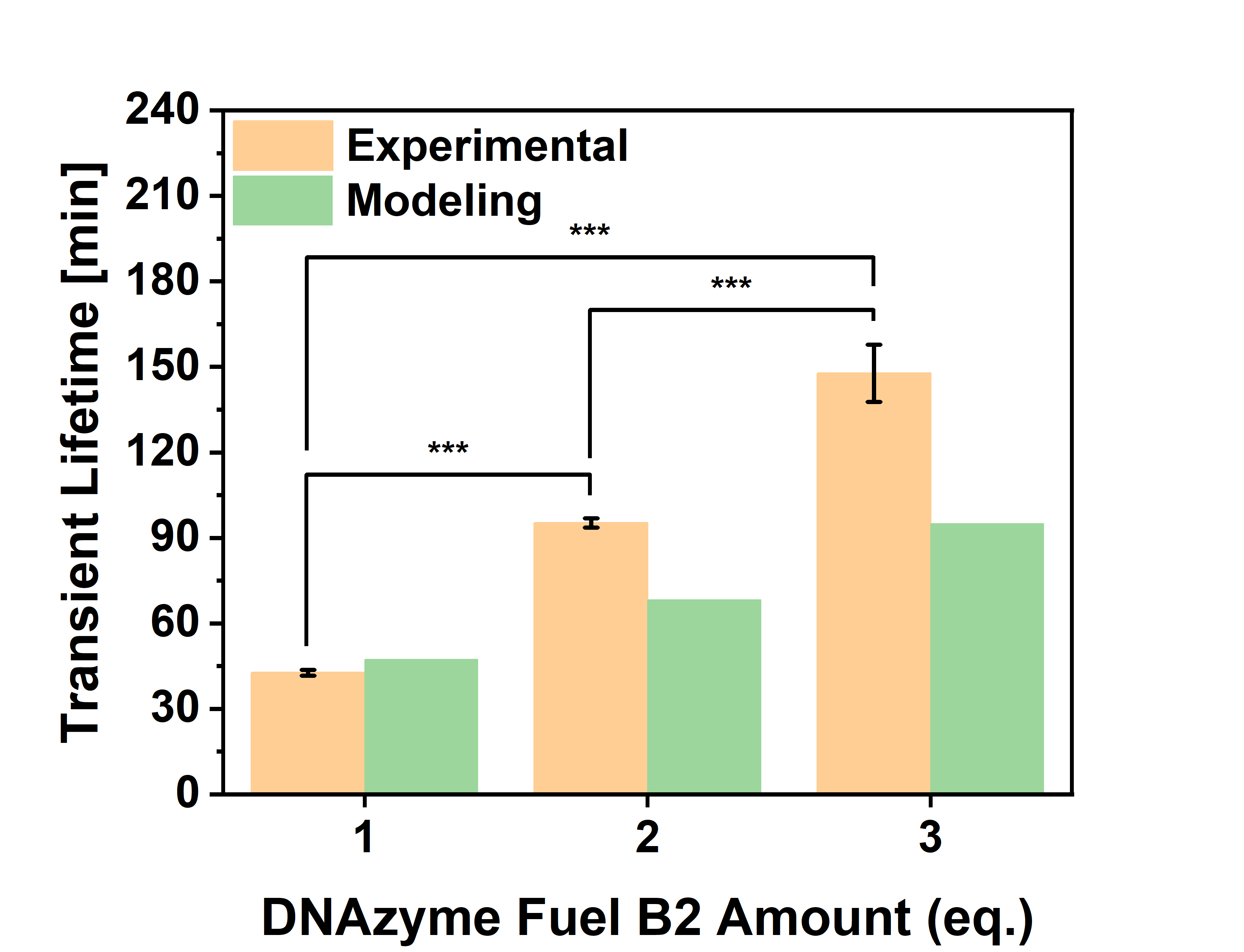


**Figure S14.** Transient lifetimes of dsDNAzyme B1/B2 following the addition of varying amounts of ssDNAzyme fuel B2. Transient lifetimes determined experimentally (yellow) and from kinetic modeling (green) in the presence of 1, 2, or 3 eq. DNAzyme fuel B2, DNAzyme reporter strand B (2.5 µM), and T7 (0.5 U µL^-1^). The statistical significance was determined via analysis of variance (ANOVA) using the Origin 2024b software. Data are presented as mean ± standard deviation (SD) of three independent experiments (n = 3); error bars represent the SD. Significance: n.s. = not significant; * = p < 0.05; ** = p < 0.01; *** = p < 0.001.


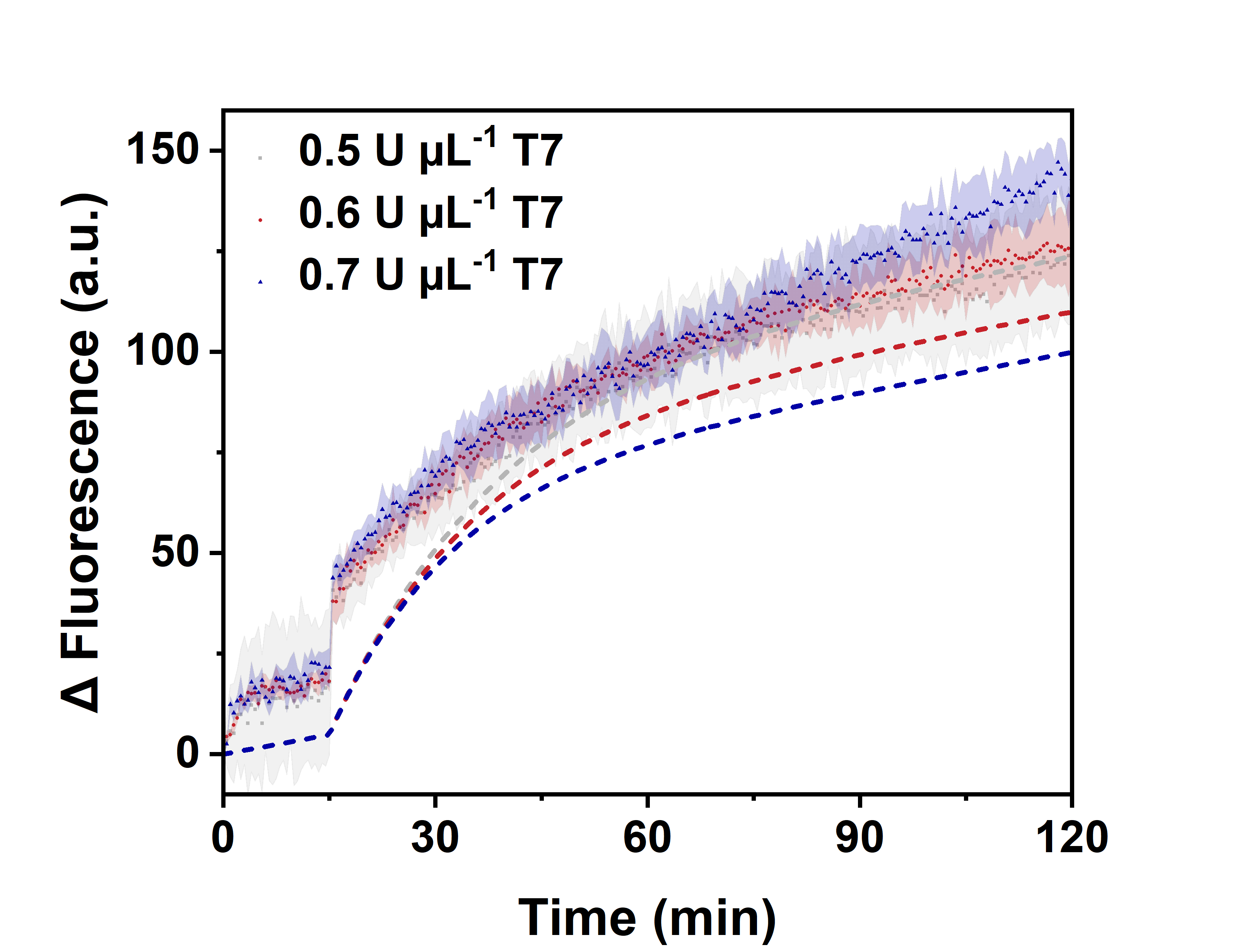


**Figure S15. Dissipative control of dsDNAzyme B1/B2 activity at varying T7 concentrations.** Δ Fluorescence intensity at 520 nm, after excitation at 490 nm, is plotted against time in the presence of 0.5, 0.6, or 0.7 U µL^-1^ T7, DNAzyme reporter strand B (2.5 µM), and ssDNAzyme fuel B2 (1 eq.). Data are presented as mean ± standard deviation (SD) of three independent experiments (n = 3); error bars represent the SD.


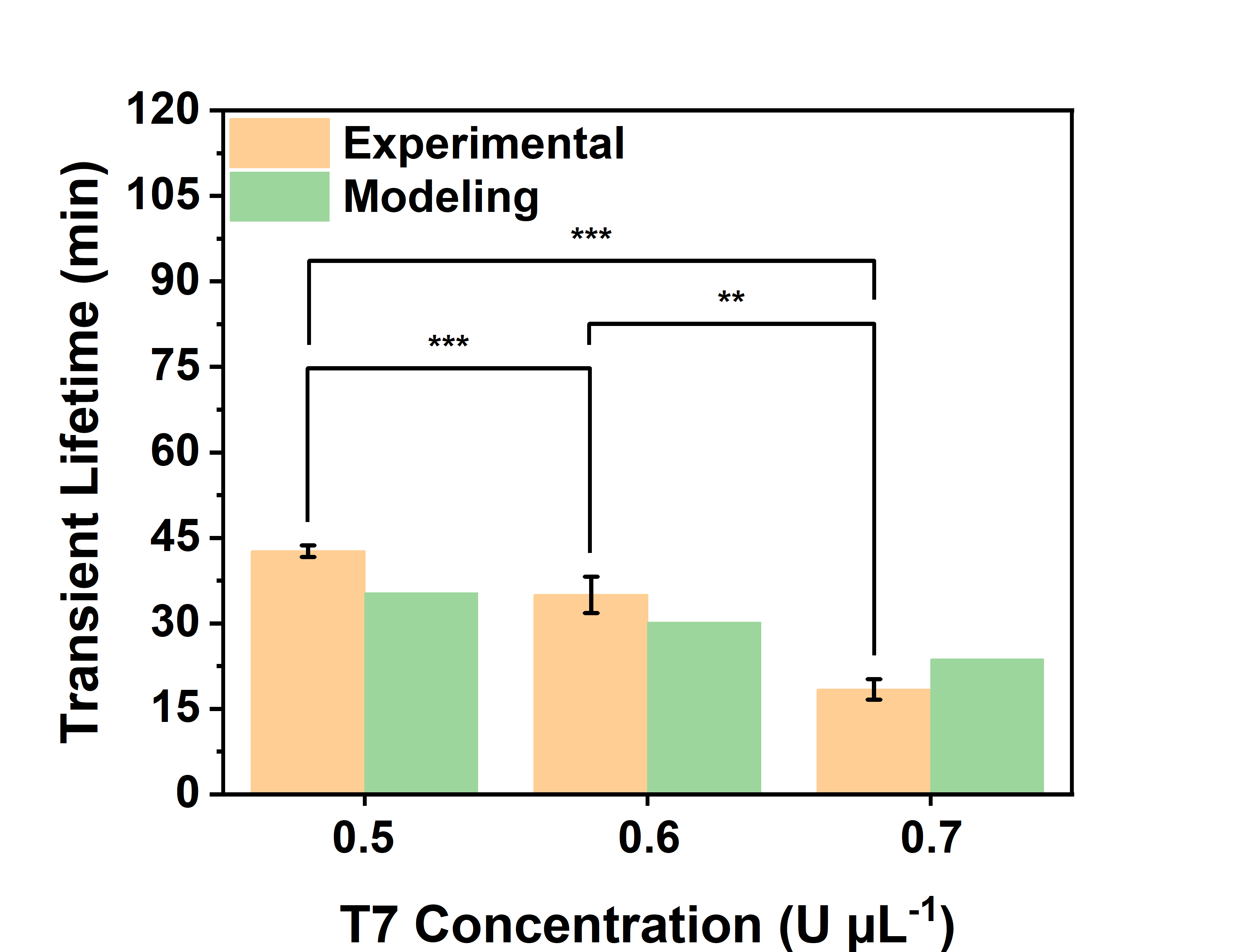


**Figure S16.** Transient lifetimes of dsDNAzyme B1/B2 at varying T7 concentrations. Transient lifetimes determined experimentally (yellow) and through kinetic modeling (green) in the presence of 0.5, 0.6, or 0.7 U µL^-1^ T7, DNAzyme reporter strand B (2.5 µM), and ssDNAzyme fuel B2 (1 eq.). Data are presented as mean ± standard deviation (SD) of three independent experiments (n = 3); error bars represent the SD. The statistical significance was determined via analysis of variance (ANOVA) using the Origin 2024b software. Significance: n.s. = not significant; * = p < 0.05; ** = p < 0.01; *** = p < 0.001.


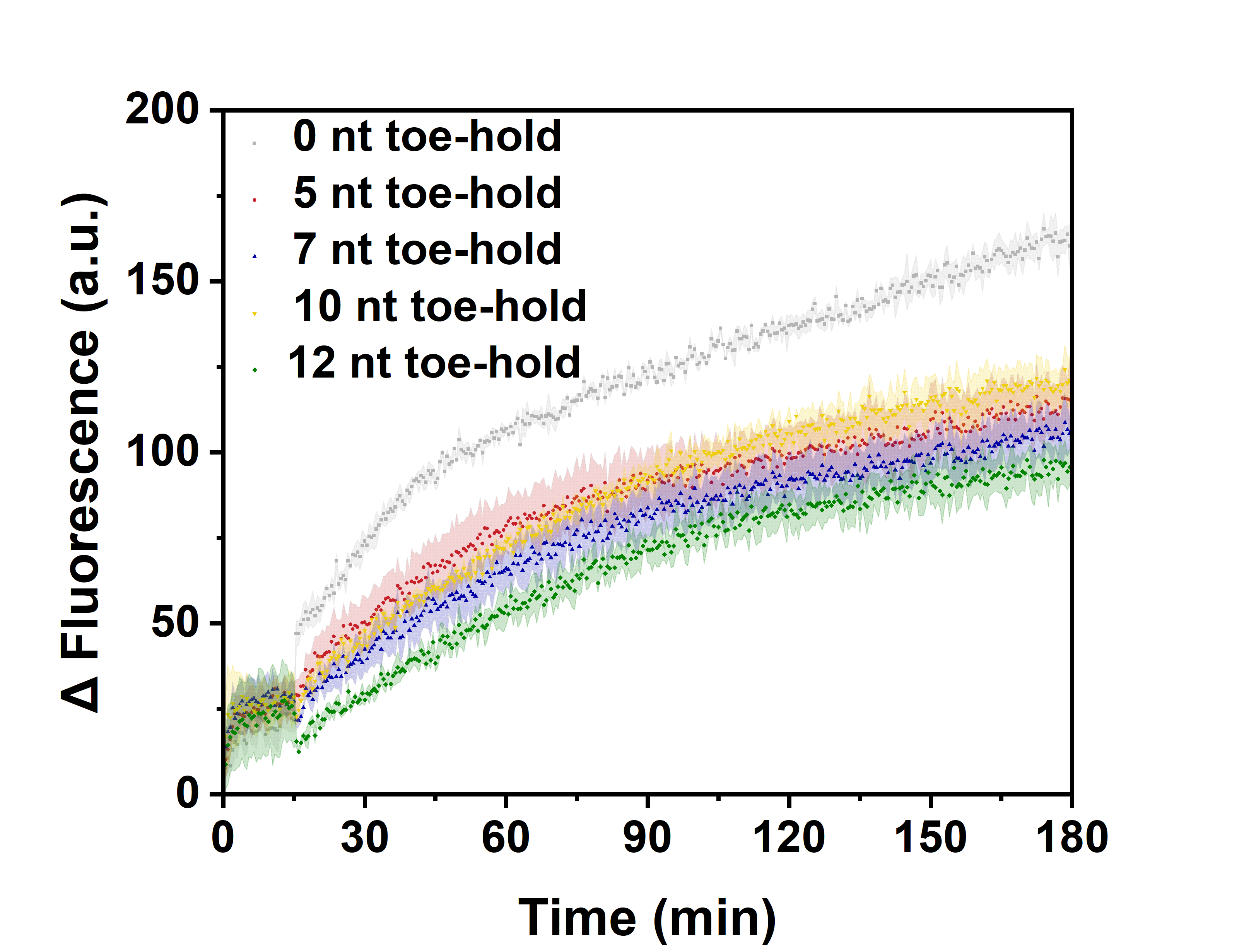


**Figure S17. Dissipative control over dsDNAzyme B1/B2 activity using ssDNAzyme fuel B2 with varying toe-hold lengths.** Δ Fluorescence intensity at 520 nm, after excitation at 490 nm, is plotted against time following the addition of ssDNAzyme fuel B2 (1 eq.) with toe-holds of 0, 5, 7, 10, or 12 nts in the presence of DNAzyme reporter strand B (2.5 µM) and T7 (0.6 U µL^-1^). Data are presented as mean ± standard deviation (SD) of three independent experiments (n = 3); error bars represent the SD.


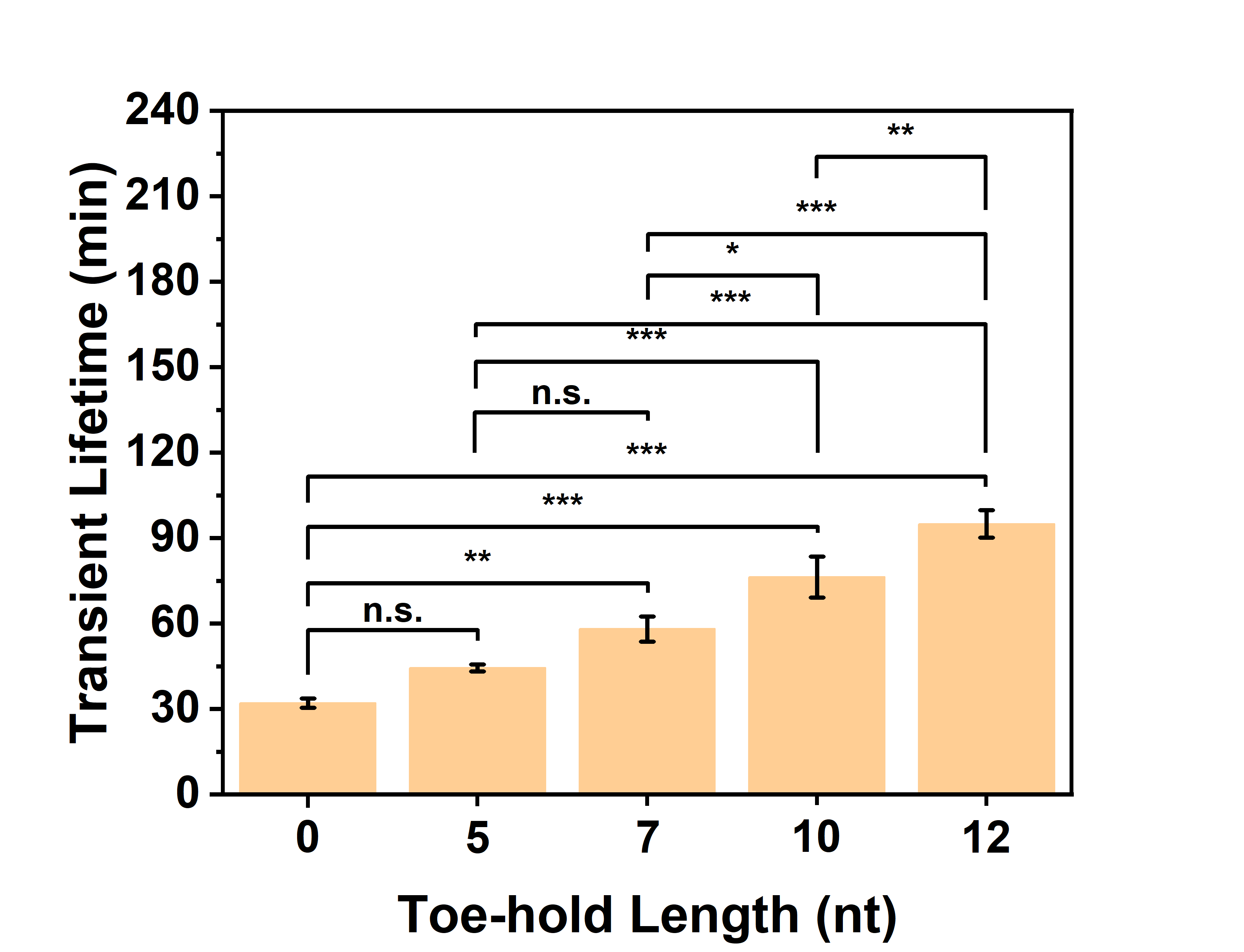


**Figure S18.** Transient lifetimes of dsDNAzyme B1/B2 **using ssDNAzyme fuel B2 with varying toe-hold lengths**. Transient lifetimes after the addition of ssDNAzyme fuel B2 (1 eq.) containing 0, 5, 7, 10 or 12 nts toe-hold, DNAzyme reporter strand B (2.5 µM), T7 (0.6 U µL^-1^) and ssDNAzyme fuel B2 (1 eq.). Data are presented as mean ± standard deviation (SD) of three independent experiments (n = 3); error bars represent the SD. The statistical significance was determined via analysis of variance (ANOVA) using the Origin 2024b software. Significance: n.s. = not significant; * = p < 0.05; ** = p < 0.01; *** = p < 0.001.


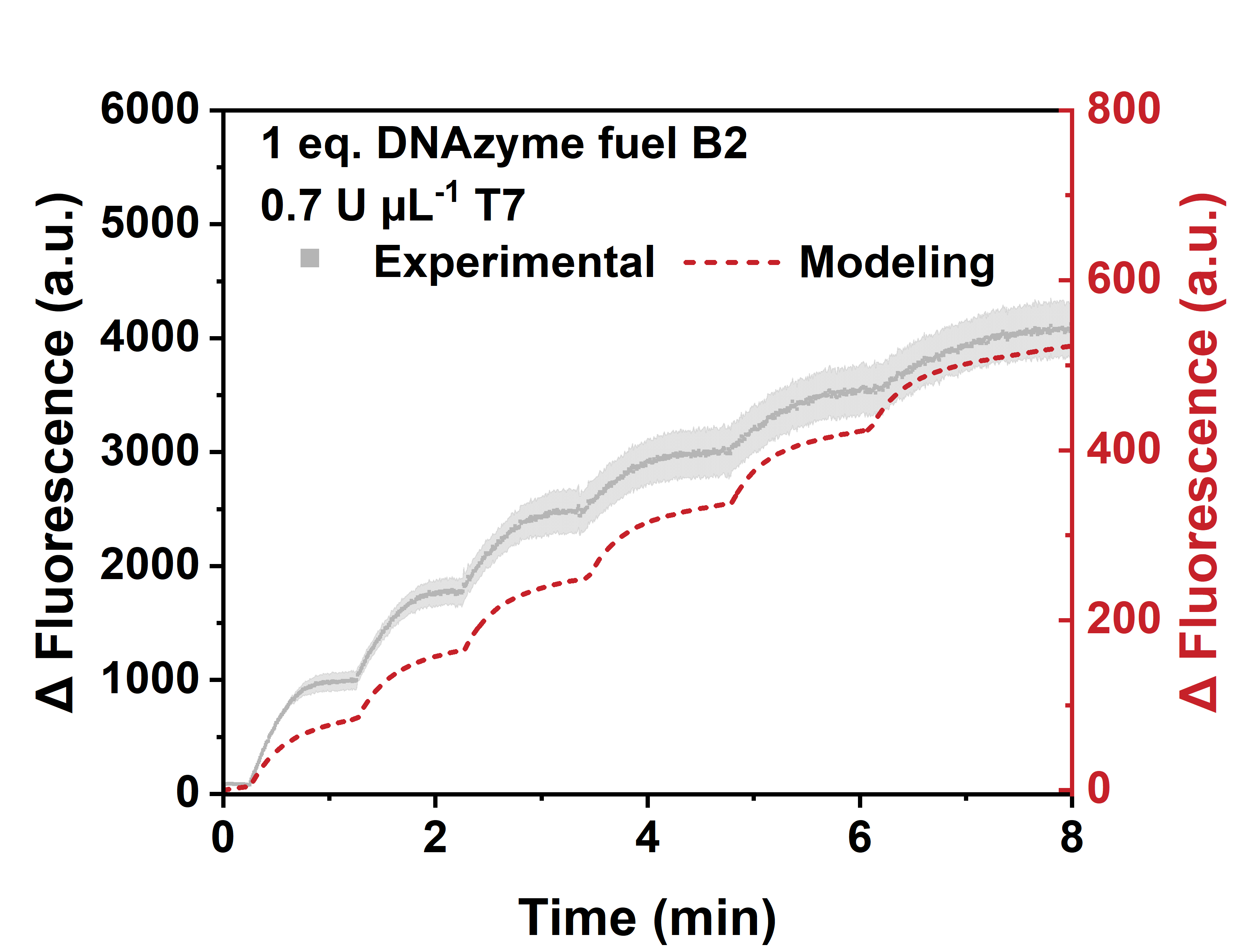


Figure S19. Successive additions of ssDNAzyme fuel B2 in the presence of 0.7 U µL^-1^ T7. Fluorescence intensity at 520 nm was monitored for 15 min before adding ssDNAzyme fuel B2 (1 eq.) after excitation at 490 nm. Consecutive additions of ssDNAzyme fuel B2 (1 eq.) were performed at 15, 76, 137, 206.5, 287.4, and 366 min in the presence of DNAzyme reporter strand B (2.5 µM) and T7 (0.7 U µL^-1^). Grey: Δ fluorescence intensity at 520 nm after excitation at 490 nm (left y-axis) of dsDNAzyme B1/B2 (2.5 µM) activity in the presence of T7 (0.7 U µL^-1^), plotted against time (experimental data). Red: Modeled Δ fluorescence intensity (right y-axis) plotted against time for dsDNAzyme B1/B2 (2.5 µM) activity in the presence of T7 (0.7 U µL^-1^). The Δ fluorescence intensity of the modeled data was rescaled due to higher starting fluorescence in the successive addition experiment compared to the single experiments used to determine the kinetic parameters for the model. Data are presented as mean ± standard deviation (SD) of three independent experiments (n = 3); error bars represent the SD.


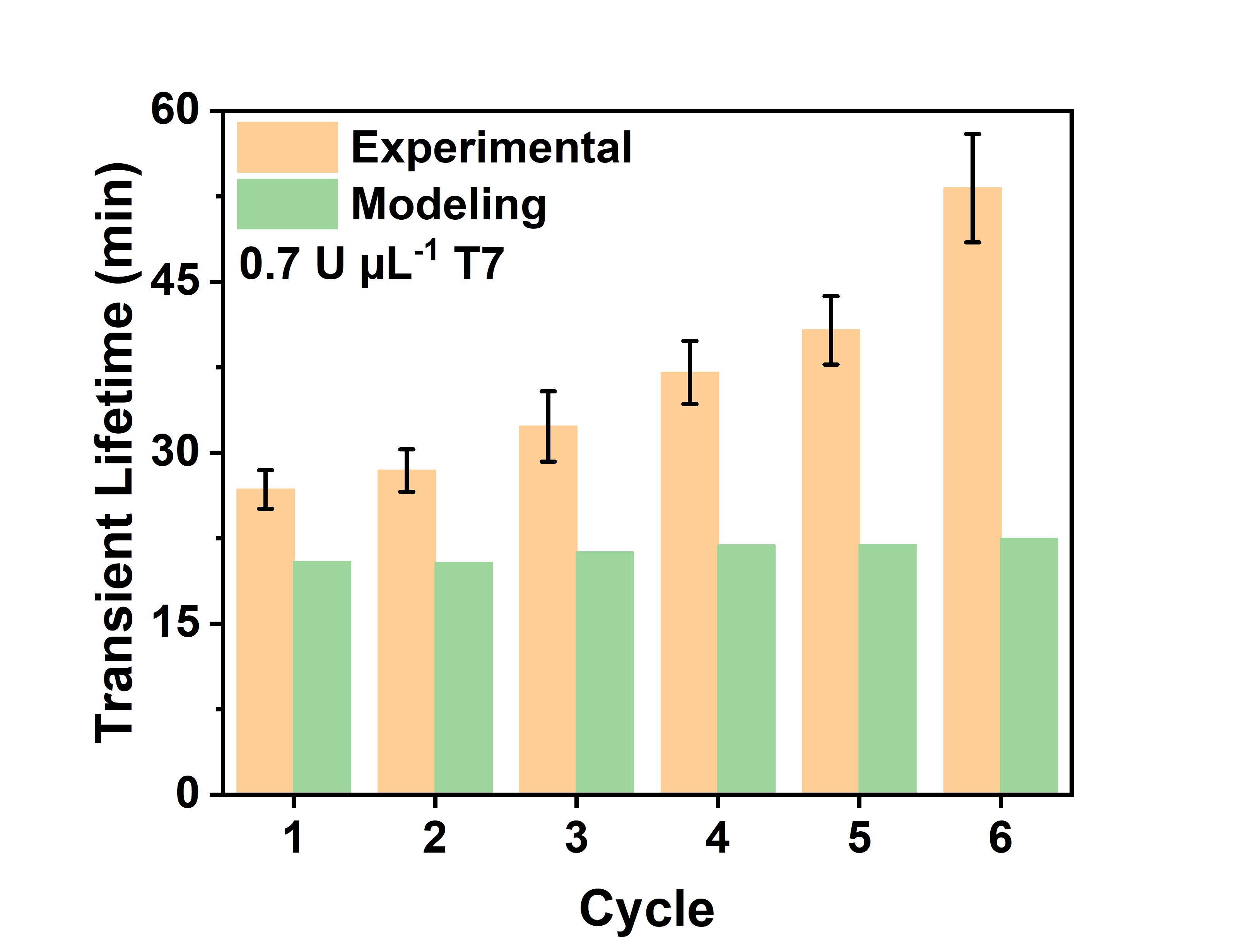
Figure S20. Transient lifetime of dsDNAzyme B1/B2 in the presence of 0.7 U µL^-1^ T7 as a function of consecutive fuel additions. Transient lifetimes of dsDNAzyme B1/B2 (2.5 µM) in the presence of T7 (0.7 U µL^-1^) determined from experimental data (yellow) and predicted transient lifetimes (green) obtained from the kinetic model. Data are presented as mean ± standard deviation (SD) of three independent experiments (n = 3); error bars represent the SD.

Table S6. Statistical significance determined by analysis of variance (ANOVA) using the Origin 2024b software for the successive additions of the ssDNAzyme fuel B2 in the presence of 0.7 U µL^-1^ T7.

| Cycle Nr. | 1 | 2 | 3 | 4 | 5 | 6 |
| --- | --- | --- | --- | --- | --- | --- |
| 1 |  | n.s. | n.s. | * | ** | *** |
| 2 | n.s. |  | n.s. | n.s. | * | *** |
| 3 | n.s. | n.s. |  | n.s. | n.s. | *** |
| 4 | * | n.s. | n.s. |  | n.s. | ** |
| 5 | ** | * | n.s. | n.s. |  | * |
| 6 | *** | *** | *** | ** | * |  |

Significance: n.s. = not significant; * = p < 0.05; ** = p < 0.01; *** = p < 0.001.


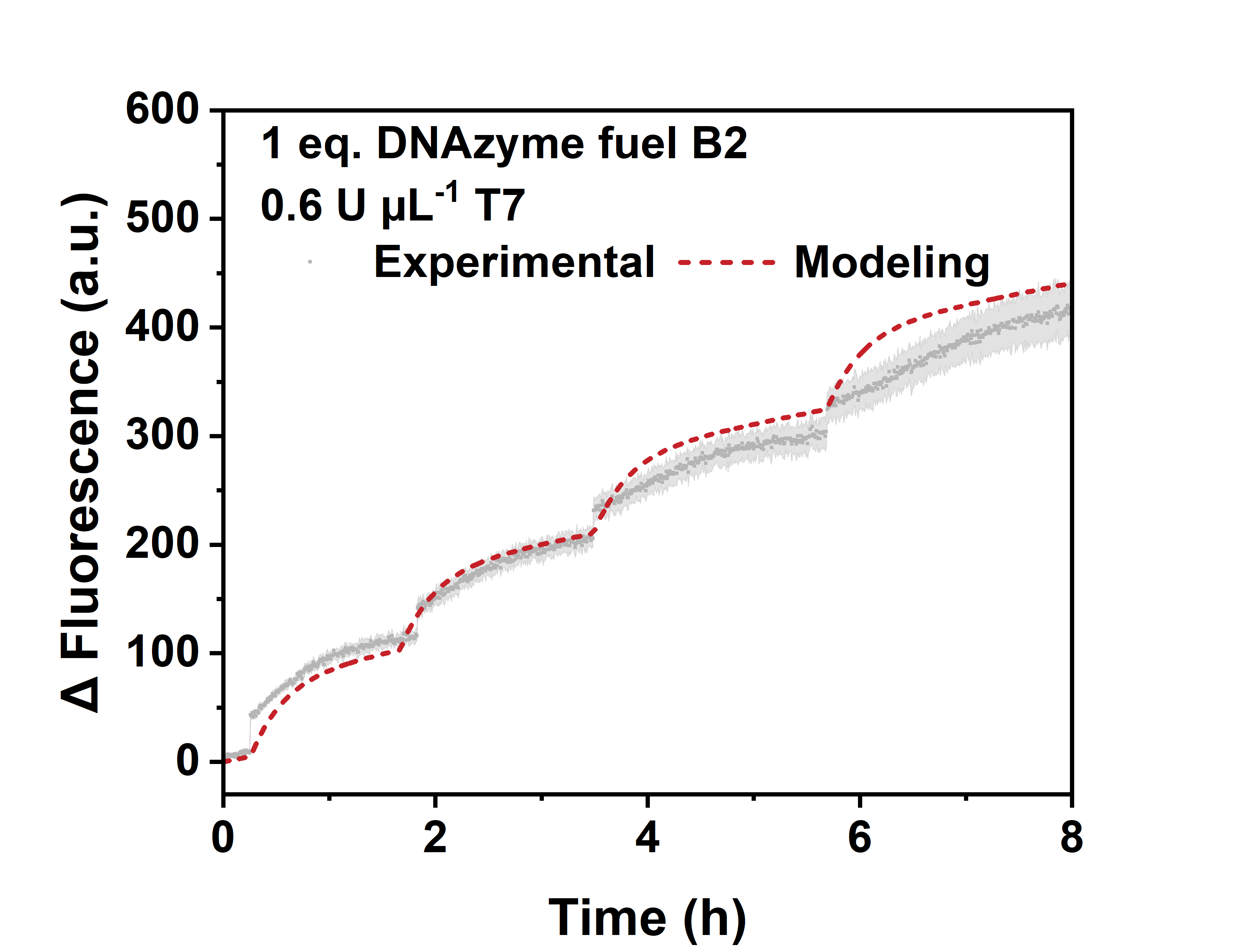


Figure S21. Successive additions of ssDNAzyme fuel B2 in the presence of 0.6 U µL^-1^ T7. Fluorescence intensity at 520 nm was monitored for 15 min before the addition of ssDNAzyme fuel B2 (1 eq.) after excitation at 490 nm. Consecutive additions of ssDNAzyme fuel B2 (1 eq.) were performed at 15, 110, 209.5, and 341.5 min in the presence of DNAzyme reporter strand B (2.5 µM) and T7 (0.6 U µL^-1^). Grey: Δ fluorescence intensity at 520 nm after excitation at 490 nm of dsDNAzyme B1/B2 (2.5 µM) activity in the presence of T7 (0.6 U µL^-1^) plotted against time (experimental data). Red: Modeled Δ fluorescence intensity plotted against time for dsDNAzyme B1/B2 (2.5 µM) activity in the presence of T7 (0.6 U µL^-1^). Data are presented as mean ± standard deviation (SD) of three independent experiments (n = 3); error bars represent the SD.


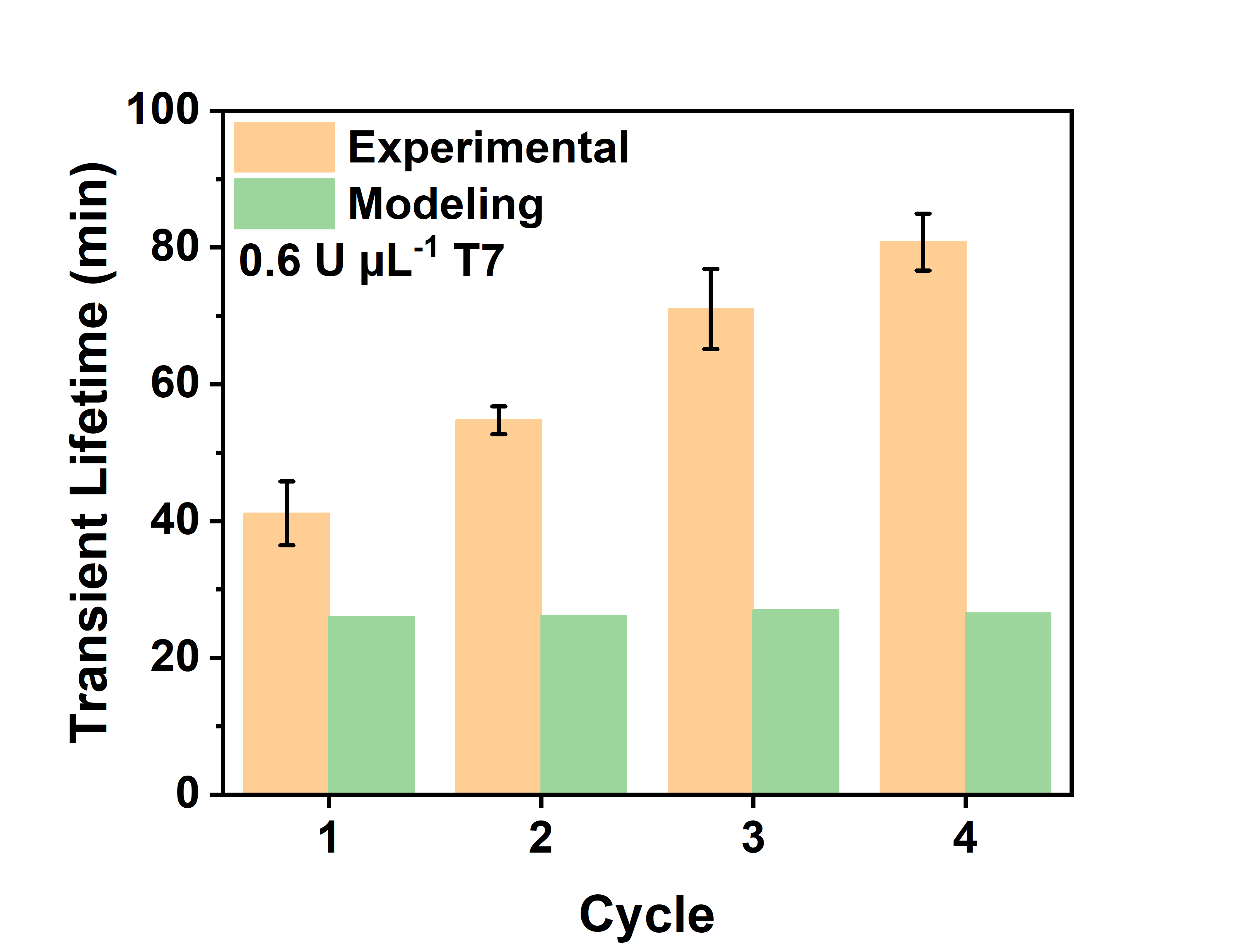


Figure S22. Transient lifetime of dsDNAzyme B1/B2 in the presence of 0.6 U µL^-1^ T7 as a function of consecutive fuel additions. Transient lifetimes of dsDNAzyme B1/B2 (2.5 µM) in the presence of T7 (0.6 U µL^-1^) determined from experimental data (yellow) and the predicted transient lifetimes (green) obtained from the kinetic model. Data are presented as mean ± standard deviation (SD) of three independent experiments (n = 3); error bars represent the SD.

Table S7. Statistical significance determined by analysis of variance (ANOVA) using the Origin 2024b software for the successive additions of the ssDNAzyme fuel B2 in the presence of 0.6 U µL^-1^ T7.

| Cycle Nr. | 1 | 2 | 3 | 4 |
| --- | --- | --- | --- | --- |
| 1 |  | n.s. | *** | *** |
| 2 | n.s. |  | * | ** |
| 3 | *** | * |  | n.s. |
| 4 | *** | ** | n.s. |  |

Significance: n.s. = not significant; * = p < 0.05; ** = p < 0.01; *** = p < 0.001.


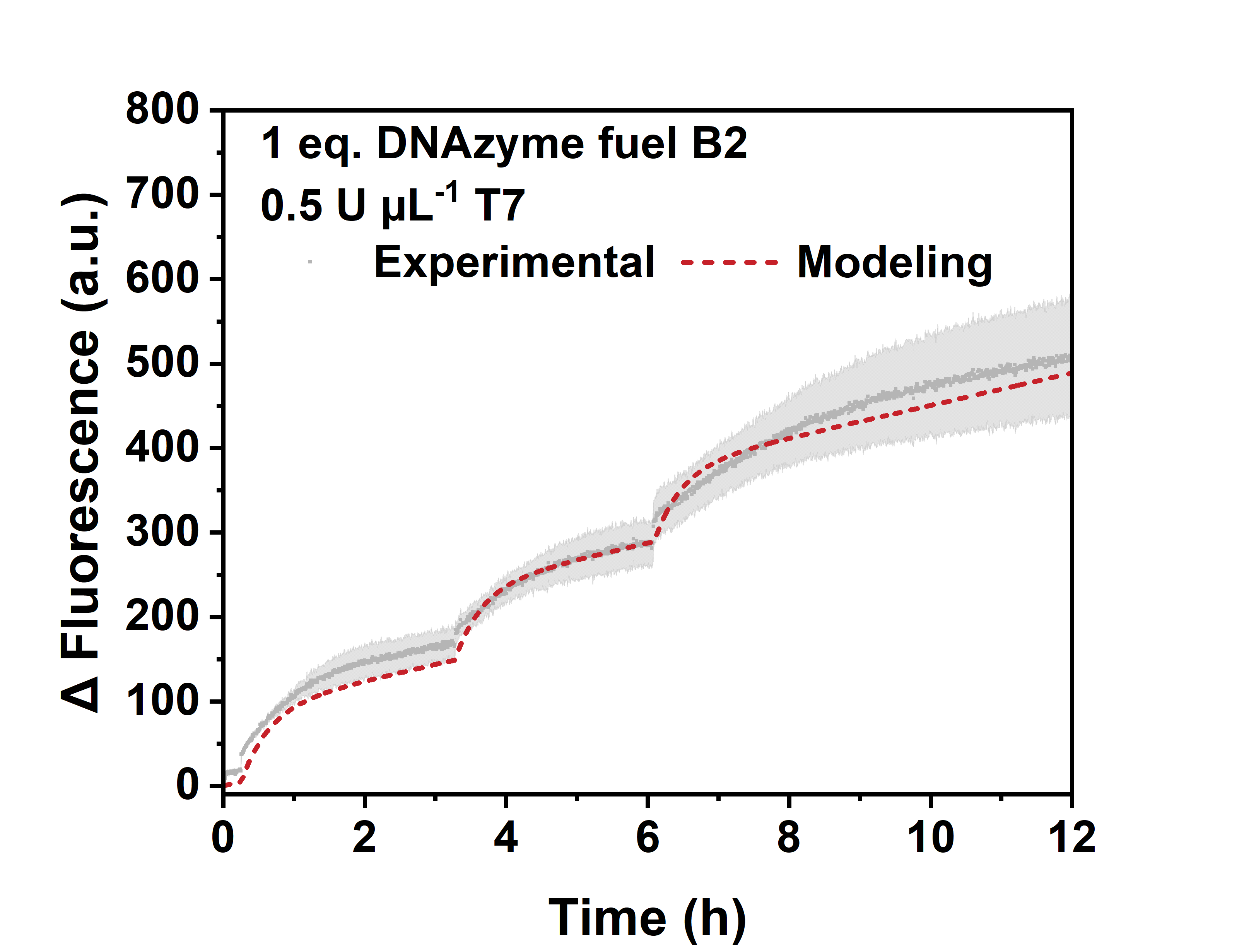


Figure S23. Successive additions of ssDNAzyme fuel B2 in the presence of 0.5 U µL^-1^ T7. Fluorescence intensity at 520 nm was monitored for 15 min before the addition of ssDNAzyme fuel B2 (1 eq.) after excitation at 490 nm. Consecutive additions of ssDNAzyme fuel B2 (1 eq.) were performed at 15, 197, and 365.5 min in the presence of DNAzyme reporter strand B (2.5 µM) and T7 (0.5 U µL^-1^). Grey: Δ fluorescence intensity at 520 nm after excitation at 490 nm for dsDNAzyme B1/B2 (2.5 µM) activity in the presence of T7 (0.5 U µL^-1^), plotted against time (experimental data). Red: Modeled Δ fluorescence intensity plotted against time for dsDNAzyme B1/B2 (2.5 µM) activity in the presence of T7 (0.5 U µL^-1^). Data are presented as mean ± standard deviation (SD) of three independent experiments (n = 3); error bars represent the SD.

**
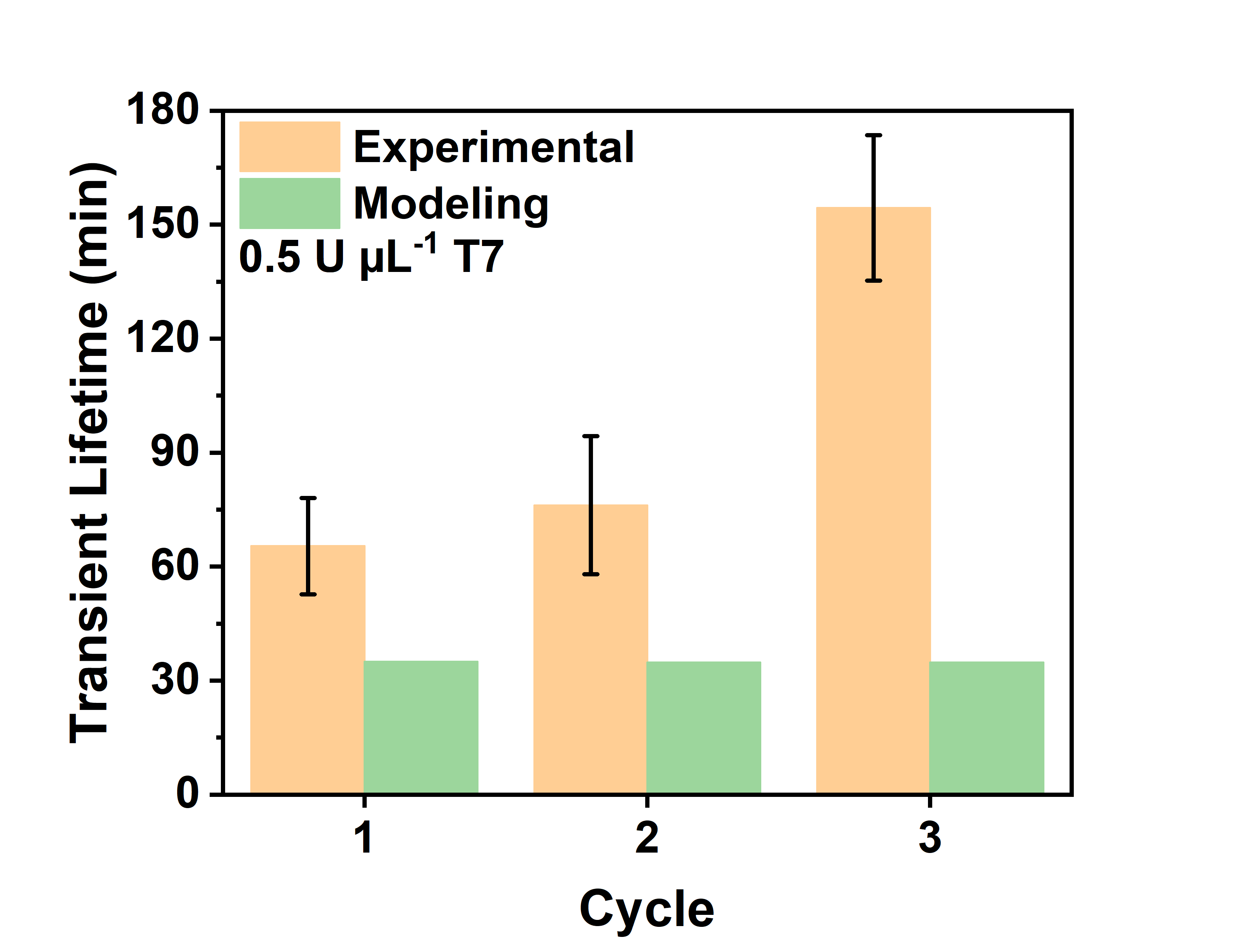
**

Figure S24. Transient lifetime of dsDNAzyme B1/B2 in the presence of 0.5 U µL^-1^ T7 as a function of consecutive fuel additions. Transient lifetimes of dsDNAzyme B1/B2 (2.5 µM) in the presence of T7 (0.5 U µL^-1^) determined from experimental data (yellow) and predicted transient lifetimes (green) obtained from the kinetic model. Data are presented as mean ± standard deviation (SD) of three independent experiments (n = 3); error bars represent the SD.

Table S8. Statistical significance determined by analysis of variance (ANOVA) using the Origin 2024b software for the successive additions of the ssDNAzyme fuel B2 in the presence of 0.5 U µL^-1^ T7.

| Cycle Nr. | 1 | 2 | 3 |
| --- | --- | --- | --- |
| 1 |  | n.s. | ** |
| 2 | n.s. |  | ** |
| 3 | ** | ** |  |

Significance: n.s. = not significant; * = p < 0.05; ** = p < 0.01; *** = p < 0.001.


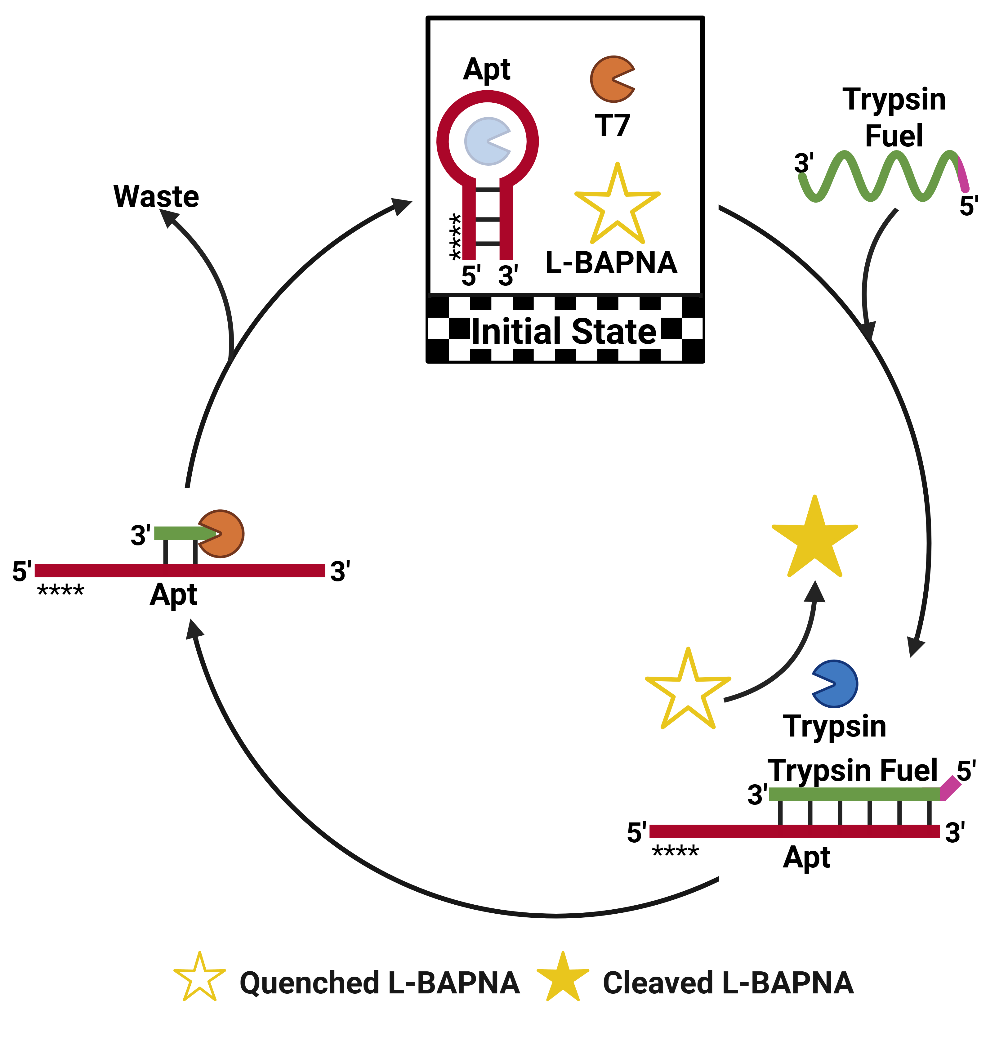


**Figure S25.** Schematic representation of T7-governed dissipative regulation of trypsin. **Step 1:** Addition of the trypsin fuel activates trypsin. **Step 2:** T7 digests the trypsin fuel, leading to dissipation. **Step 3:** Rebinding of the aptamer deactivates trypsin. Four consecutive PS bonds at the 5′-end protect the trypsin aptamer from T7 digestion (indicated by four black stars). Created by Biorender.com


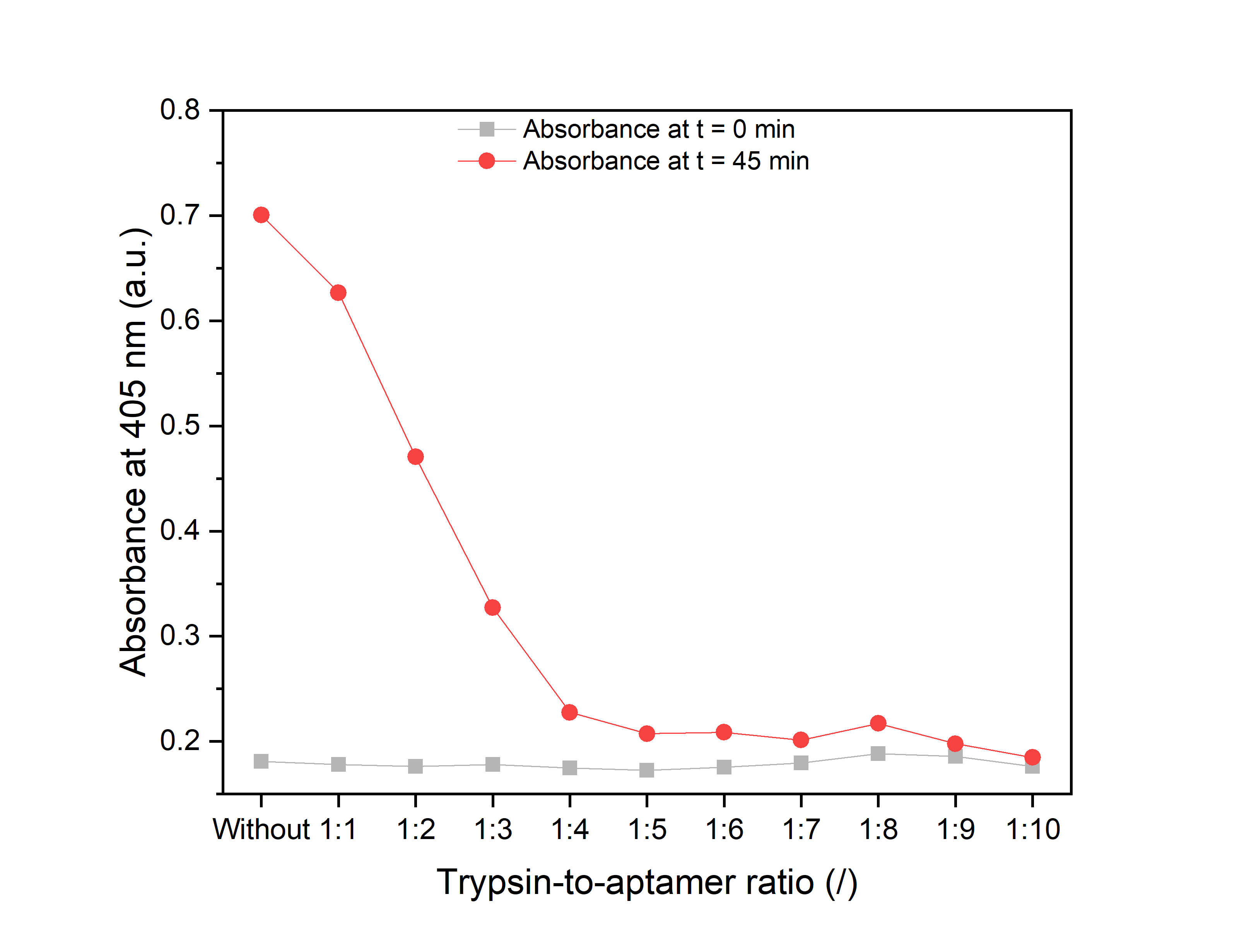
Figure S26. Determination of the trypsin-to-aptamer ratio required for the complete deactivation of trypsin. Gray: Initial absorbance at 405 nm for the various aptamer-to-trypsin ratio mixtures in the presence of L-BAPNA (100 µM). Red: Absorbance at 405 nm after 45 min for the same aptamer-to-trypsin ratio mixtures in the presence of L-BAPNA (100 µM).


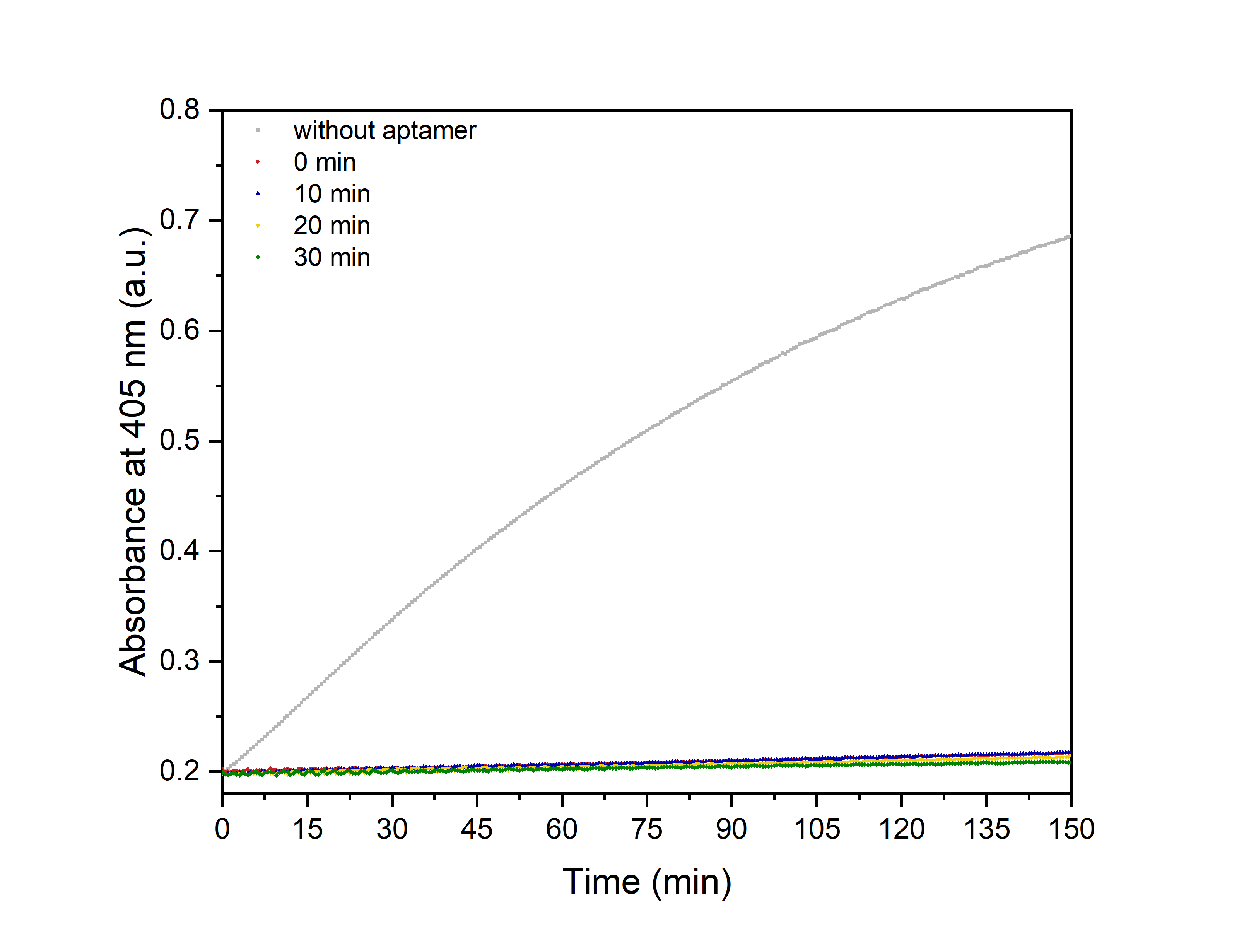
Figure S27. Incubation time required for complete deactivation of trypsin using 10 eq. of aptamer. Gray: Absorbance at 405 nm over time in the presence of L-BAPNA (100 µM) and trypsin (0.1 µM) (positive control). Red/Blue/Yellow/Green: Absorbance at 405 nm over time in the presence of L-BAPNA (100 µM) and trypsin (0.1 µM), incubated with the aptamer (1 µM) for 0, 10, 20, and 30 min, respectively.


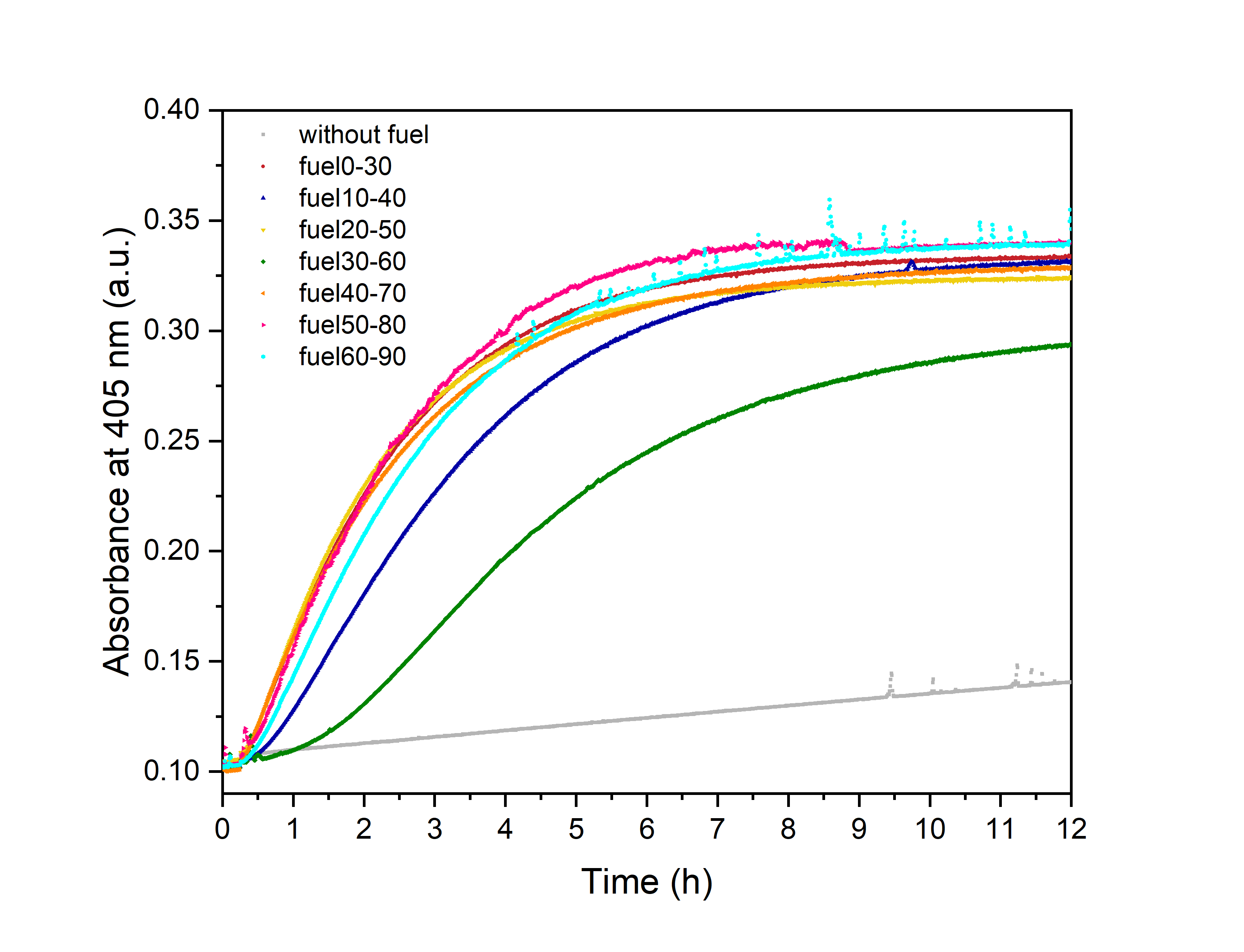
**Figure S28.** Shortening of the trypsin fuel. To reduce the amount of waste generated with each DNA fuel addition, the 90-nt complementary strand of the trypsin aptamer was shortened to 30-nt fragments and tested for their ability to reactivate trypsin through hybridization with the aptamer. Gray: Absorbance at 405 nm over time in the presence of L-BAPNA (100 µM) and trypsin (0.1 µM) after a 30 min incubation with the aptamer (1 µM) (negative control). Red/Blue/Yellow/Green/Orange/Pink/Cyan: Absorbance at 405 nm over time showing trypsin (0.1 µM) activity after 30 min of incubation with the aptamer (1 µM) in the presence of various DNA fragments (2 µM).


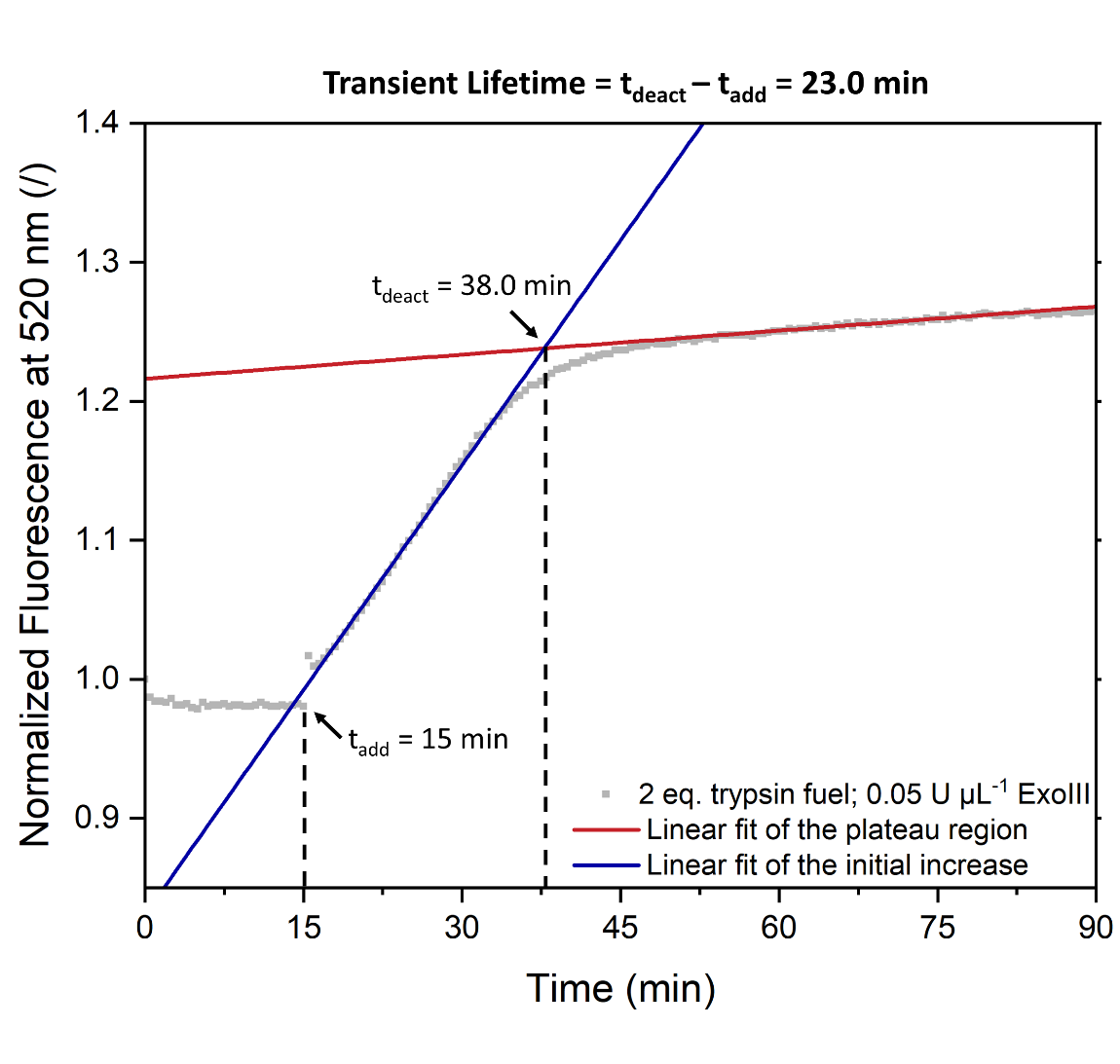
Figure S29. Graphical determination of the transient lifetime exemplified with the Exo III-regulated trypsin system. Gray: Normalized absorbance at 405 nm of trypsin activity in the presence of Exo III (0.05 U µL^-1^), plotted against time following excitation at 490 nm. Red: Linear fit of the plateau region of the curve. Blue: Linear fit of the initial increase of the curve. The fits were performed using OriginPro software (2024b).

**
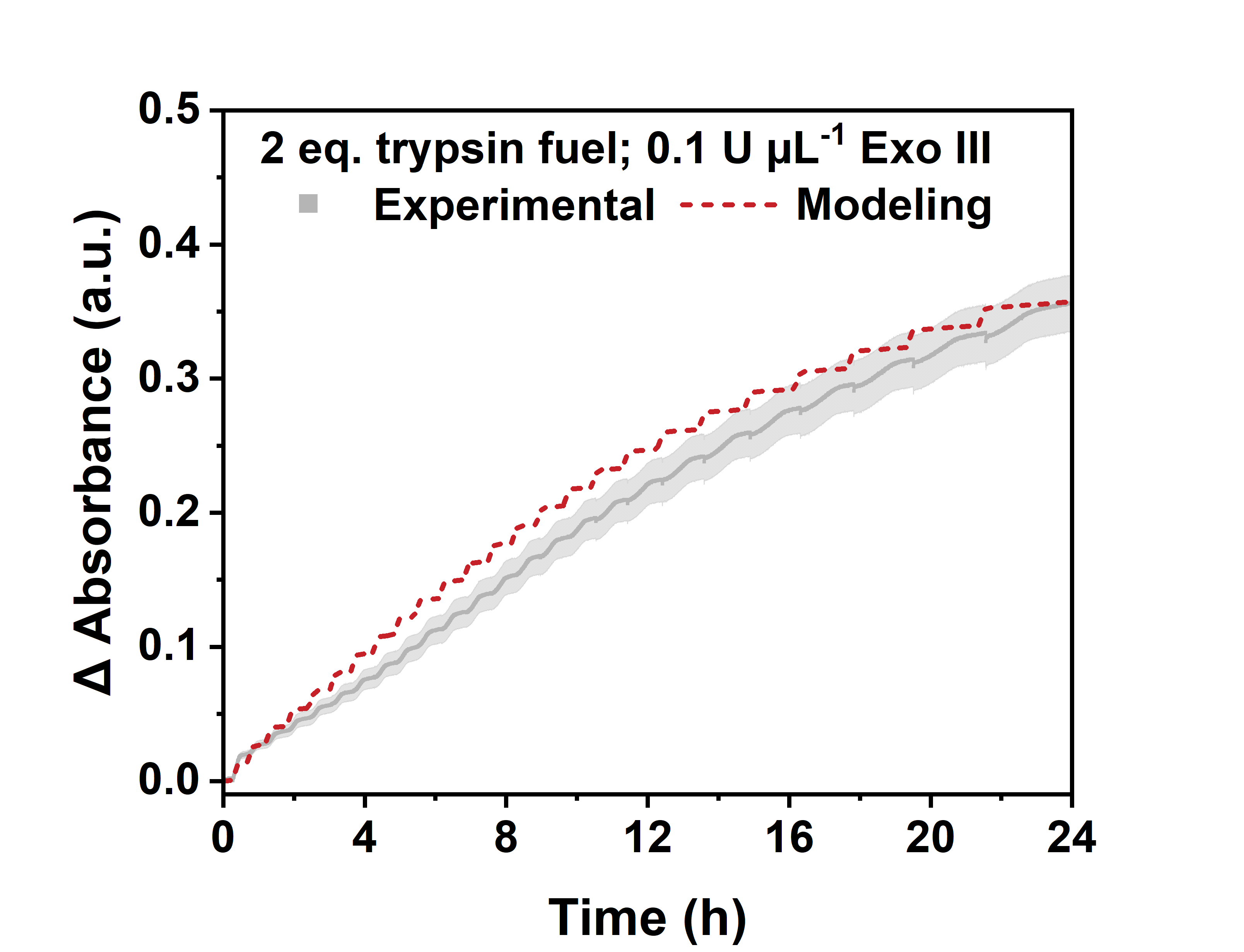
**

Figure S30. Successive additions of trypsin fuel in the presence of 0.1 U µL^-1^ Exo III. Δ Absorbance at 405 nm was monitored for 15 min before the first addition of trypsin fuel (2 eq.). Consecutive additions of trypsin fuel (2 eq.) were performed at 15, 40, 71, 107, 144, 180.5, 216, 252.5, 288.5, 323.5, 365, 407, 449, 488.5, 530.5, 576.5, 624, 677, 765.5, 806, 884, 968.5, 1058.5, 1158.5, and 1281.5 min in the presence of Exo III (0.1 U µL^-1^). Grey: Δ absorbance at 405 nm reflecting trypsin activity (0.1 µM) activity in the presence of Exo III (0.1 U µL^-1^) plotted against the time (experimental data). Red: Δ absorbance at 405 nm reflecting trypsin (0.1 µM) activity in the presence of Exo III (0.1 U µL^-1^) plotted against the time (modeled data). Data are presented as mean ± standard deviation (SD) of three independent experiments (n = 3); error bars represent the SD.


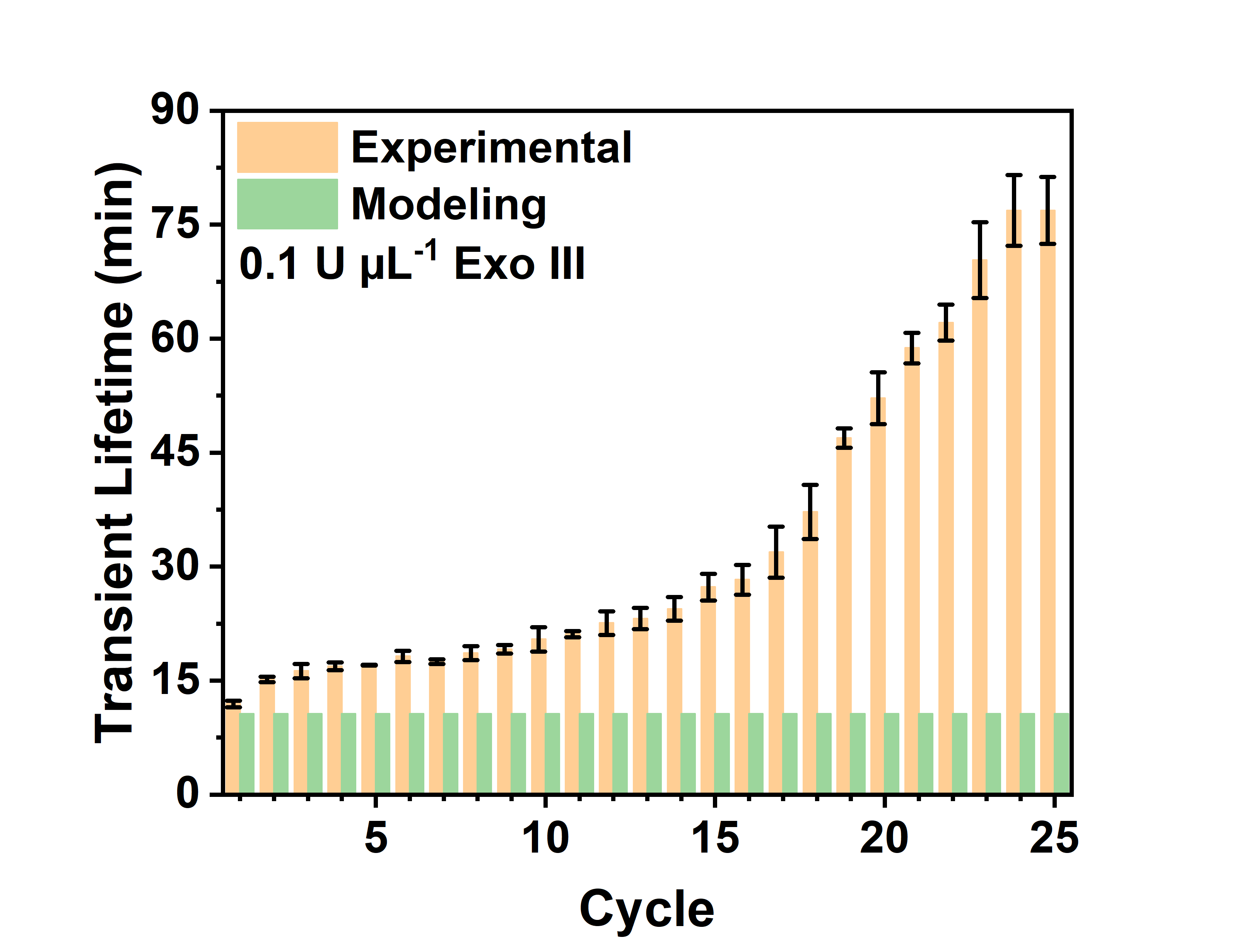


Figure S31. Transient lifetime of trypsin in the presence of 0.1 U µL^-1^ Exo III as a function of consecutive fuel additions. The transient lifetimes of trypsin (0.1 µM) in the presence of Exo III (0.1 U µL^-1^) were determined from experimental data (yellow) and compared to the predicted transient lifetimes (green) derived from the kinetic model. Data are presented as mean ± standard deviation (SD) of three independent experiments (n = 3); error bars represent the SD.

**Table S9.** Statistical significance determined by analysis of variance (ANOVA) using the Origin 2024b software for the successive additions of trypsin fuel in the presence of 0.1 U µL^-1^ ExoIII.

| Cycle Nr. | 1 | 2 | 3 | 4 | 5 | 6 | 7 | 8 | 9 | 10 | 11 | 12 | 13 | 14 | 15 | 16 | 17 | 18 | 19 | 20 | 21 | 22 | 23 | 24 | 25 |
| --- | --- | --- | --- | --- | --- | --- | --- | --- | --- | --- | --- | --- | --- | --- | --- | --- | --- | --- | --- | --- | --- | --- | --- | --- | --- |
| 1 |  | n.s. | n.s. | n.s. | n.s. | n.s. | n.s. | n.s. | n.s. | n.s. | * | ** | ** | *** | *** | *** | *** | *** | *** | *** | *** | *** | *** | *** | *** |
| 2 | n.s. |  | n.s. | n.s. | n.s. | n.s. | n.s. | n.s. | n.s. | n.s. | n.s. | n.s. | n.s. | * | *** | *** | *** | *** | *** | *** | *** | *** | *** | *** | *** |
| 3 | n.s. | n.s. |  | n.s. | n.s. | n.s. | n.s. | n.s. | n.s. | n.s. | n.s. | n.s. | n.s. | n.s. | ** | *** | *** | *** | *** | *** | *** | *** | *** | *** | *** |
| 4 | n.s. | n.s. | n.s. |  | n.s. | n.s. | n.s. | n.s. | n.s. | n.s. | n.s. | n.s. | n.s. | n.s. | ** | ** | *** | *** | *** | *** | *** | *** | *** | *** | *** |
| 5 | n.s. | n.s. | n.s. | n.s. |  | n.s. | n.s. | n.s. | n.s. | n.s. | n.s. | n.s. | n.s. | n.s. | ** | ** | *** | *** | *** | *** | *** | *** | *** | *** | *** |
| 6 | n.s. | n.s. | n.s. | n.s. | n.s. |  | n.s. | n.s. | n.s. | n.s. | n.s. | n.s. | n.s. | n.s. | * | ** | *** | *** | *** | *** | *** | *** | *** | *** | *** |
| 7 | n.s. | n.s. | n.s. | n.s. | n.s. | n.s. |  | n.s. | n.s. | n.s. | n.s. | n.s. | n.s. | n.s. | * | ** | *** | *** | *** | *** | *** | *** | *** | *** | *** |
| 8 | n.s. | n.s. | n.s. | n.s. | n.s. | n.s. | n.s. |  | n.s. | n.s. | n.s. | n.s. | n.s. | n.s. | n.s. | * | *** | *** | *** | *** | *** | *** | *** | *** | *** |
| 9 | n.s. | n.s. | n.s. | n.s. | n.s. | n.s. | n.s. | n.s. |  | n.s. | n.s. | n.s. | n.s. | n.s. | n.s. | * | *** | *** | *** | *** | *** | *** | *** | *** | *** |
| 10 | n.s. | n.s. | n.s. | n.s. | n.s. | n.s. | n.s. | n.s. | n.s. |  | n.s. | n.s. | n.s. | n.s. | n.s. | n.s. | ** | *** | *** | *** | *** | *** | *** | *** | *** |
| 11 | * | n.s. | n.s. | n.s. | n.s. | n.s. | n.s. | n.s. | n.s. | n.s. |  | n.s. | n.s. | n.s. | n.s. | n.s. | ** | *** | *** | *** | *** | *** | *** | *** | *** |
| 12 | ** | n.s. | n.s. | n.s. | n.s. | n.s. | n.s. | n.s. | n.s. | n.s. | n.s. |  | n.s. | n.s. | n.s. | n.s. | * | *** | *** | *** | *** | *** | *** | *** | *** |
| 13 | ** | n.s. | n.s. | n.s. | n.s. | n.s. | n.s. | n.s. | n.s. | n.s. | n.s. | n.s. |  | n.s. | n.s. | n.s. | n.s. | *** | *** | *** | *** | *** | *** | *** | *** |
| 14 | *** | * | n.s. | n.s. | n.s. | n.s. | n.s. | n.s. | n.s. | n.s. | n.s. | n.s. | n.s. |  | n.s. | n.s. | n.s. | *** | *** | *** | *** | *** | *** | *** | *** |

Significance: n.s. = not significant; * = p < 0.05; ** = p < 0.01; *** = p < 0.001.

| 15 | *** | *** | ** | ** | ** | * | * | n.s. | n.s. | n.s. | n.s. | n.s. | n.s. | n.s. |  | n.s. | n.s. | * | *** | *** | *** | *** | *** | *** | *** |
| --- | --- | --- | --- | --- | --- | --- | --- | --- | --- | --- | --- | --- | --- | --- | --- | --- | --- | --- | --- | --- | --- | --- | --- | --- | --- |
| 16 | *** | *** | *** | ** | ** | ** | ** | * | * | n.s. | n.s. | n.s. | n.s. | n.s. | n.s. |  | n.s. | n.s. | * | *** | *** | *** | *** | *** | *** |
| 17 | *** | *** | *** | *** | *** | *** | *** | *** | *** | ** | ** | * | n.s. | n.s. | n.s. | n.s. |  | n.s. | n.s. | *** | *** | *** | *** | *** | *** |
| 18 | *** | *** | *** | *** | *** | *** | *** | *** | *** | *** | *** | *** | *** | *** | * | * | n.s. |  | * | *** | *** | *** | *** | *** | *** |
| 19 | *** | *** | *** | *** | *** | *** | *** | *** | *** | *** | *** | *** | *** | *** | *** | *** | *** | * |  | n.s. | ** | *** | *** | *** | *** |
| 20 | *** | *** | *** | *** | *** | *** | *** | *** | *** | *** | *** | *** | *** | *** | *** | *** | *** | *** | n.s. |  | n.s. | * | *** | *** | *** |
| 21 | *** | *** | *** | *** | *** | *** | *** | *** | *** | *** | *** | *** | *** | *** | *** | *** | *** | *** | ** | n.s. |  | n.s. | ** | *** | *** |
| 22 | *** | *** | *** | *** | *** | *** | *** | *** | *** | *** | *** | *** | *** | *** | *** | *** | *** | *** | *** | * | n.s. |  | n.s. | *** | *** |
| 23 | *** | *** | *** | *** | *** | *** | *** | *** | *** | *** | *** | *** | *** | *** | *** | *** | *** | *** | *** | *** | ** | n.s. |  | n.s. | n.s. |
| 24 | *** | *** | *** | *** | *** | *** | *** | *** | *** | *** | *** | *** | *** | *** | *** | *** | *** | *** | *** | *** | *** | *** | n.s |  | n.s. |
| 25 | *** | *** | *** | *** | *** | *** | *** | *** | *** | *** | *** | *** | *** | *** | *** | *** | *** | *** | *** | *** | *** | *** | n.s. | n.s. |  |


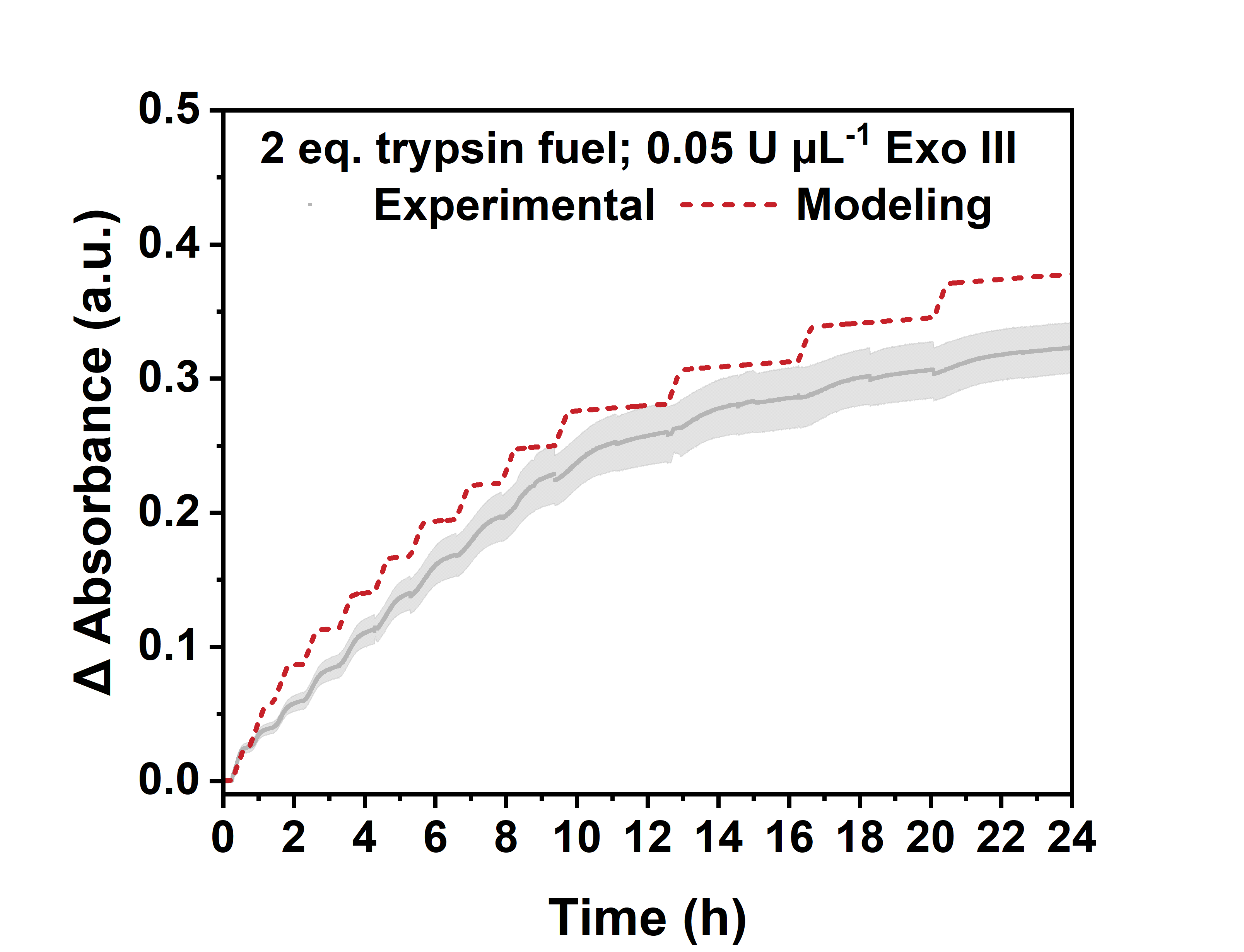


Figure S32. Successive additions of trypsin fuel in the presence of 0.05 U µL^-1^ Exo III. Δ Absorbance at 405 nm was monitored for 15 min before the first addition of trypsin fuel (2 eq.). Consecutive additions of trypsin fuel (2 eq.) were made at 15, 45, 86, 136, 197, 257, 317.5, 395, 472, 563, 754, 975.5, and 1205.5 min in the presence of Exo III (0.05 U µL^-1^). Grey: Δ absorbance at 405 nm reflecting trypsin activity (0.1 µM) activity in the presence of Exo III (0.05 U µL^-1^) plotted against the time (experimental data). Red: Δ absorbance at 405 nm reflecting trypsin (0.1 µM) activity in the presence of Exo III (0.05 U µL^-1^) plotted against the time (modeled data). Data are presented as mean ± standard deviation (SD) of three independent experiments (n = 3); error bars represent the SD.


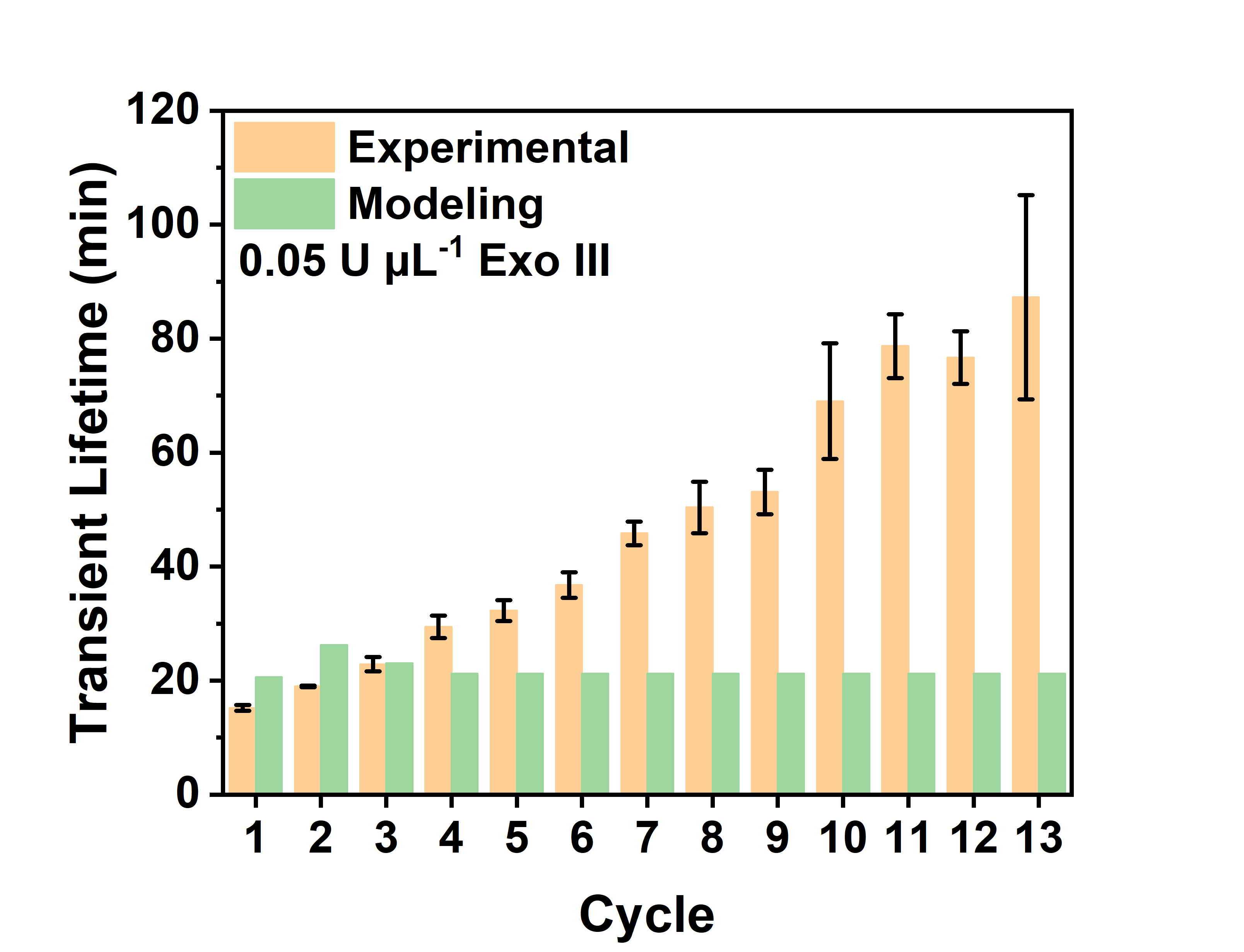


**Figure S33**. Transient lifetime of trypsin in the presence of 0.05 U µL^-1^ Exo III as a function of consecutive fuel additions. The transient lifetimes of trypsin (0.1 µM) in the presence of Exo III (0.05 U µL^-1^) were determined from experimental data (yellow) and compared to the predicted transient lifetimes (green) derived from the kinetic model. Data are presented as mean ± standard deviation (SD) of three independent experiments (n = 3); error bars represent the SD.

Table S10. Statistical significance determined by analysis of variance (ANOVA) using the Origin 2024b software for the successive additions of trypsin fuel in the presence of 0.05 U µL^-1^ Exo III.

| Cycle Nr. | 1 | 2 | 3 | 4 | 5 | 6 | 7 | 8 | 9 | 10 | 11 | 12 | 13 |
| --- | --- | --- | --- | --- | --- | --- | --- | --- | --- | --- | --- | --- | --- |
| 1 |  | n.s. | n.s. | n.s. | n.s. | n.s. | ** | *** | *** | *** | *** | *** | *** |
| 2 | n.s. |  | n.s. | n.s. | n.s. | n.s. | * | ** | *** | *** | *** | *** | *** |
| 3 | n.s. | n.s. |  | n.s. | n.s. | n.s. | n.s. | * | ** | *** | *** | *** | *** |
| 4 | n.s. | n.s. | n.s. |  | n.s. | n.s. | n.s. | n.s. | * | *** | *** | *** | *** |
| 5 | n.s. | n.s. | n.s. | n.s. |  | n.s. | n.s. | n.s. | n.s. | *** | *** | *** | *** |
| 6 | n.s. | n.s. | n.s. | n.s. | n.s. |  | n.s. | n.s. | n.s. | ** | *** | *** | *** |
| 7 | ** | * | n.s. | n.s. | n.s. | n.s. |  | n.s. | n.s. | * | ** | ** | *** |
| 8 | *** | ** | * | n.s. | n.s. | n.s. | n.s. |  | n.s. | n.s. | * | * | *** |
| 9 | *** | *** | ** | * | n.s. | n.s. | n.s. | n.s. |  | n.s. | * | * | *** |
| 10 | *** | *** | *** | *** | *** | ** | * | n.s. | n.s. |  | n.s. | n.s. | n.s. |
| 11 | *** | *** | *** | *** | *** | *** | ** | * | * | n.s. |  | n.s. | n.s. |
| 12 | *** | *** | *** | *** | *** | *** | ** | * | * | n.s. | n.s. |  | n.s. |
| 13 | *** | *** | *** | *** | *** | *** | *** | *** | *** | n.s. | n.s. | n.s. |  |

Significance: n.s. = not significant; * = p < 0.05; ** = p < 0.01; *** = p < 0.001.


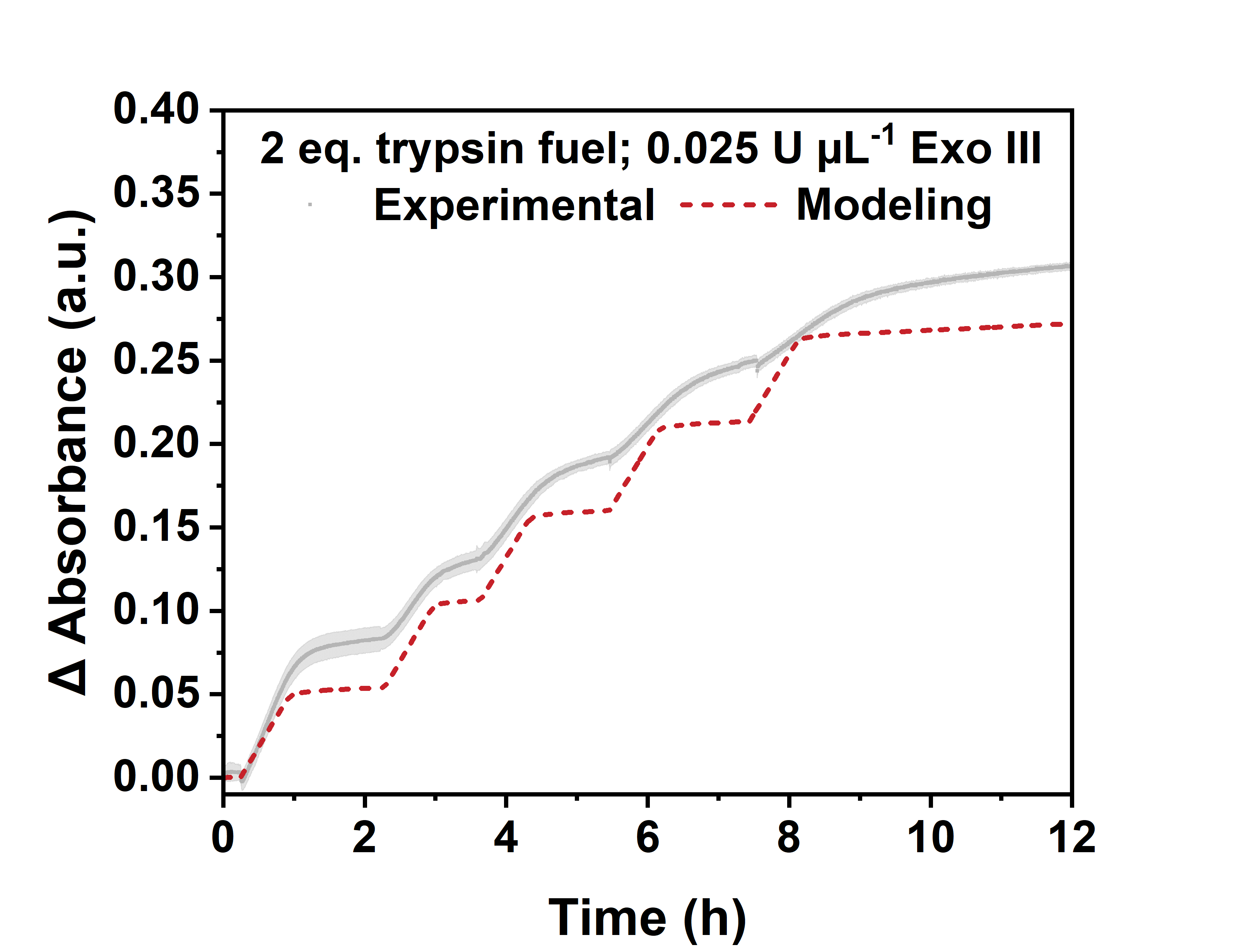
**Figure S34.** Successive additions of trypsin fuel in the presence of 0.025 U µL^-1^ Exo III. Δ Absorbance at 405 nm was monitored for 15 min before the first addition of trypsin fuel (2 eq.). Consecutive additions of trypsin fuel (2 eq.) were performed at 15, 137, 218.5, 327.5, and 446.5 min in the presence of Exo III (0.025 U µL^-1^). Grey: Δ absorbance at 405 nm reflecting trypsin activity (0.1 µM) activity in the presence of Exo III (0.025 U µL^-1^) plotted against the time (experimental data). Red: Δ absorbance at 405 nm reflecting trypsin (0.1 µM) activity in the presence of Exo III (0.025 U µL^-1^) plotted against the time (modeled data). Data are presented as mean ± standard deviation (SD) of three independent experiments (n = 3); error bars represent the SD.


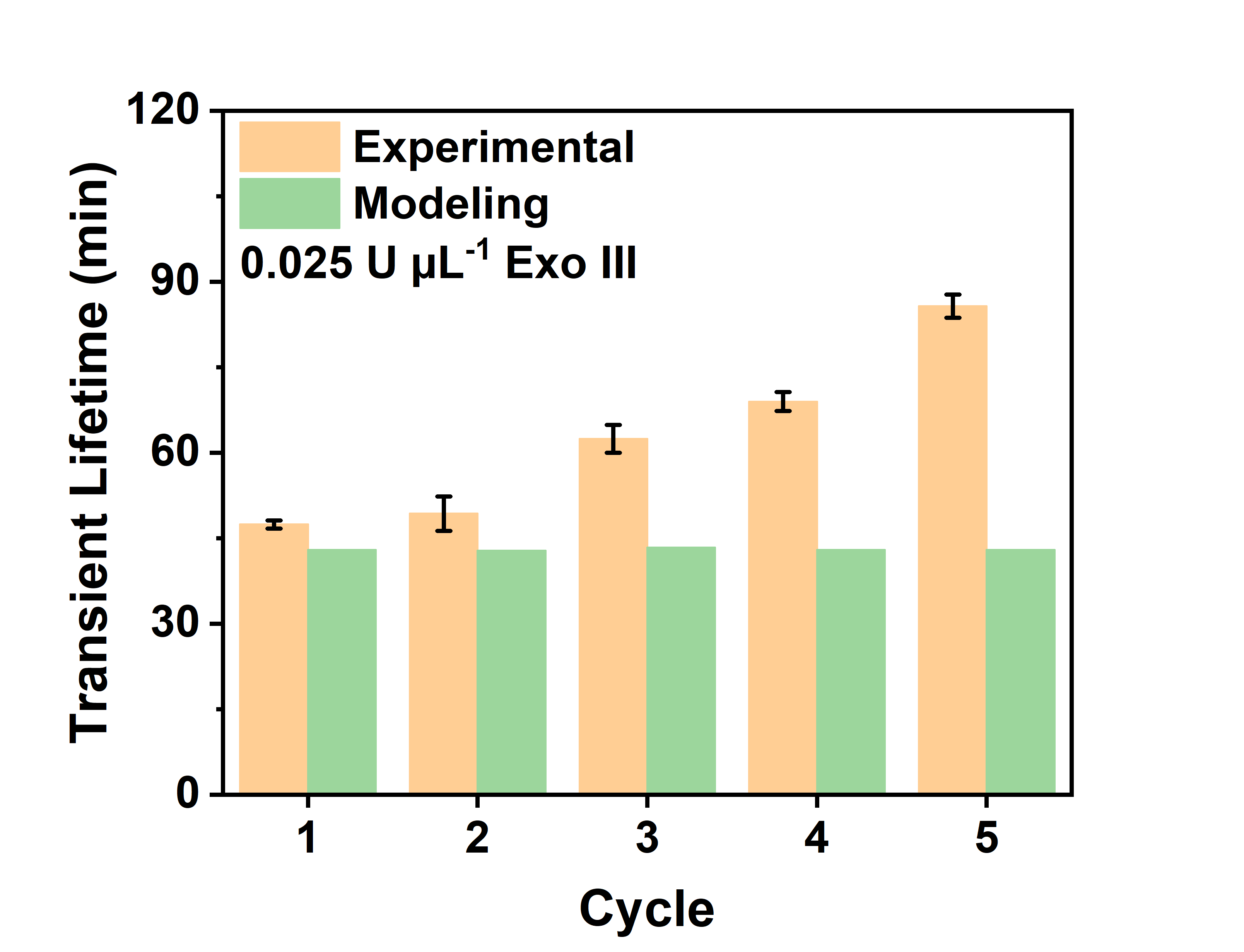


**Figure S35.** Transient lifetime of trypsin in the presence of 0.025 U µL^-1^ Exo III as a function of consecutive fuel additions. The transient lifetimes of trypsin (0.1 µM) in the presence of Exo III (0.025 U µL^-1^) were determined from experimental data (yellow) and compared to the predicted transient lifetimes (green) derived from the kinetic model. Data are presented as mean ± standard deviation (SD) of three independent experiments (n = 3); error bars represent the SD.

Table S11. Statistical significance determined by analysis of variance (ANOVA) using the Origin 2024b software for the successive additions of trypsin fuel in the presence of 0.025 U µL^-1^ Exo III.

| Cycle Nr. | 1 | 2 | 3 | 4 | 5 |
| --- | --- | --- | --- | --- | --- |
| 1 |  | n.s. | *** | *** | *** |
| 2 | n.s. |  | *** | *** | *** |
| 3 | *** | *** |  | n.s. | *** |
| 4 | *** | *** | n.s. |  | *** |
| 5 | *** | *** | *** | *** |  |

Significance: n.s. = not significant; * = p < 0.05; ** = p < 0.01; *** = p < 0.001.


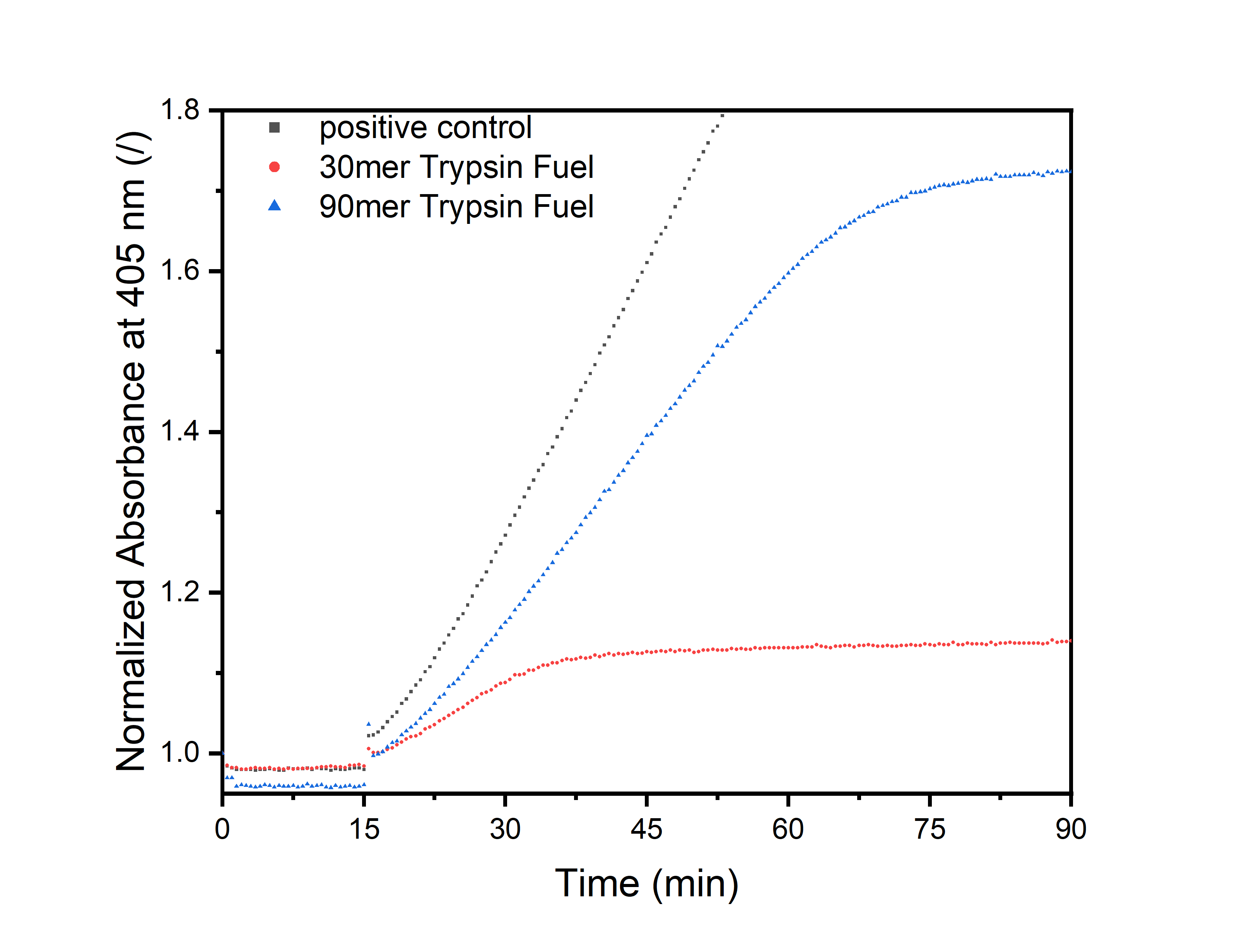


**Figure S36. Dissipative control over trypsin activity in the presence of Exo III and two different** different trypsin fuels: a shortened 30-mer and a 90-mer fully complementary to the trypsin aptamer**.** Δ absorbance at 405 nm plotted against the time in the presence of 2 eq. trypsin fuel, L-BAPNA (100 µM), and Exo III (0.05 U µL^-1^).


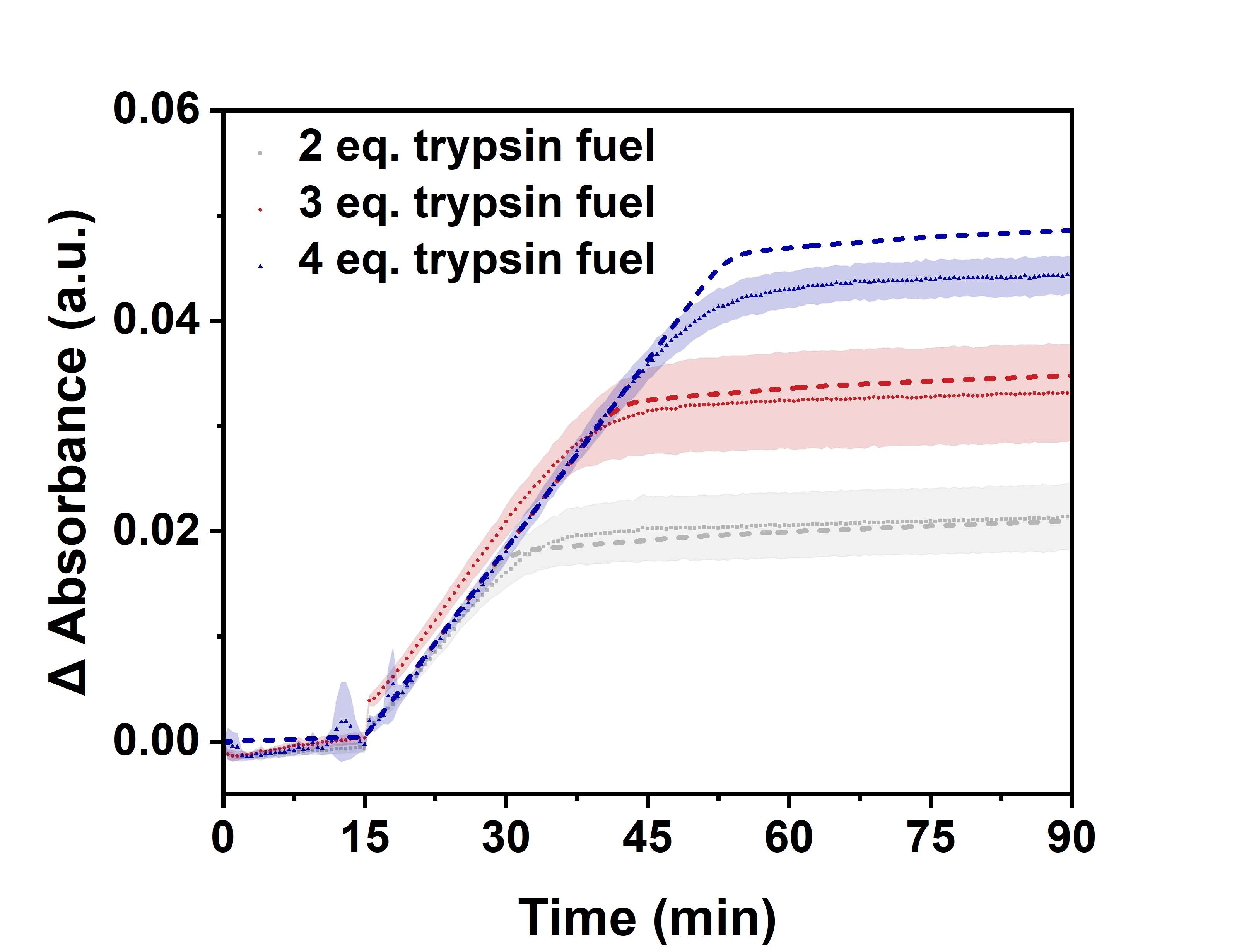


**Figure S37. Dissipative control over trypsin activity in the presence of T7 and different amounts of trypsin fuel.** Δ absorbance at 405 nm plotted against the time in the presence of 2, 3, or 4 eq. trypsin fuel, L-BAPNA (100 µM), and T7 (0.6 U µL^-1^). Data are presented as mean ± standard deviation (SD) of three independent experiments (n = 3); error bars represent the SD.


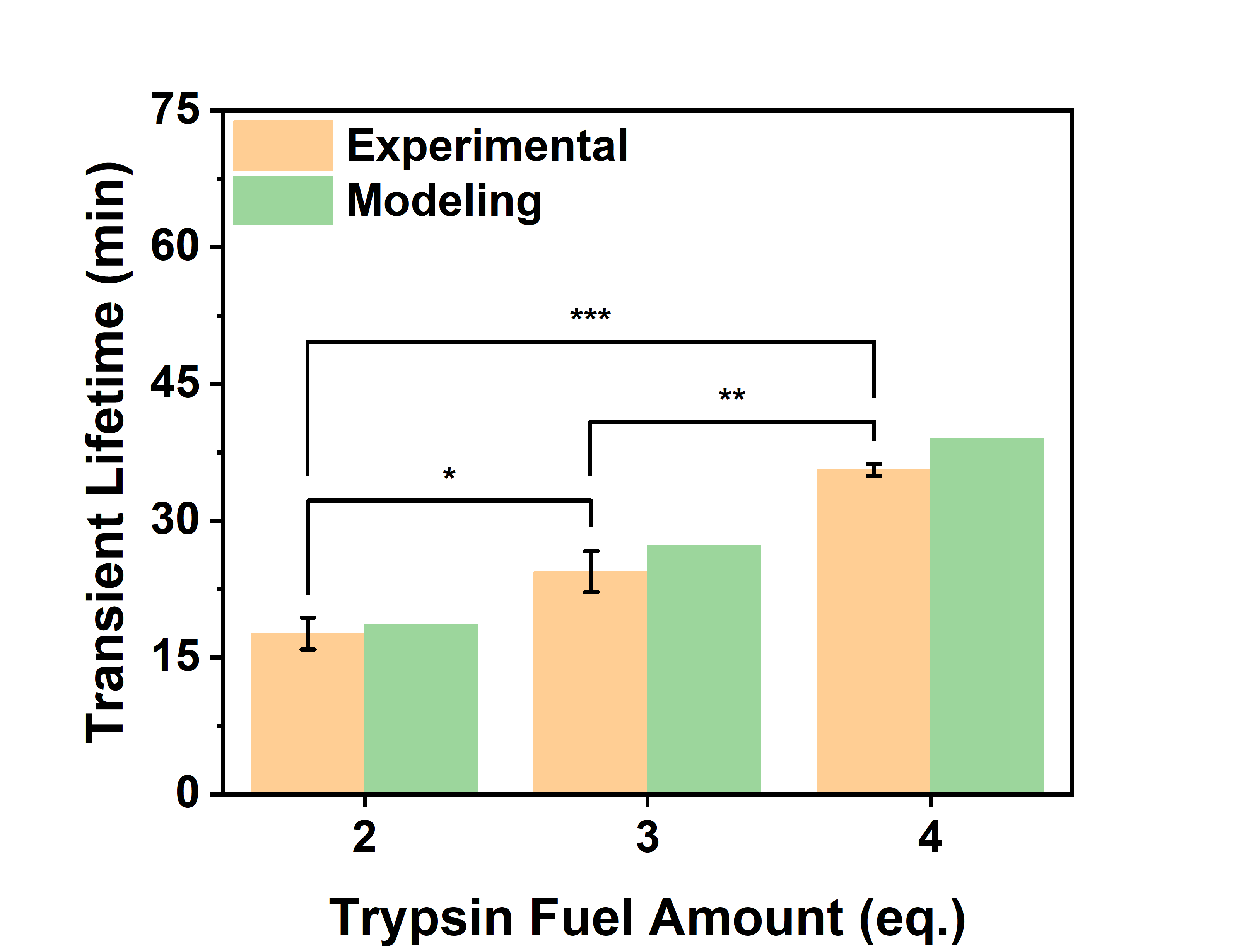


**Figure S38.** Transient lifetimes of trypsin after adding different amounts of trypsin fuel. Transient lifetime determined experimentally (yellow) and from kinetic modeling (green) in the presence of 2, 3, or 4 eq. trypsin fuel, L-BAPNA (100 µM), and T7 (0.6 U µL^-1^). Data are presented as mean ± standard deviation (SD) of three independent experiments (n = 3); error bars represent the SD. The statistical significance was determined via analysis of variance (ANOVA) using the Origin 2024b software. Significance: n.s. = not significant; * = p < 0.05; ** = p < 0.01; *** = p < 0.001.


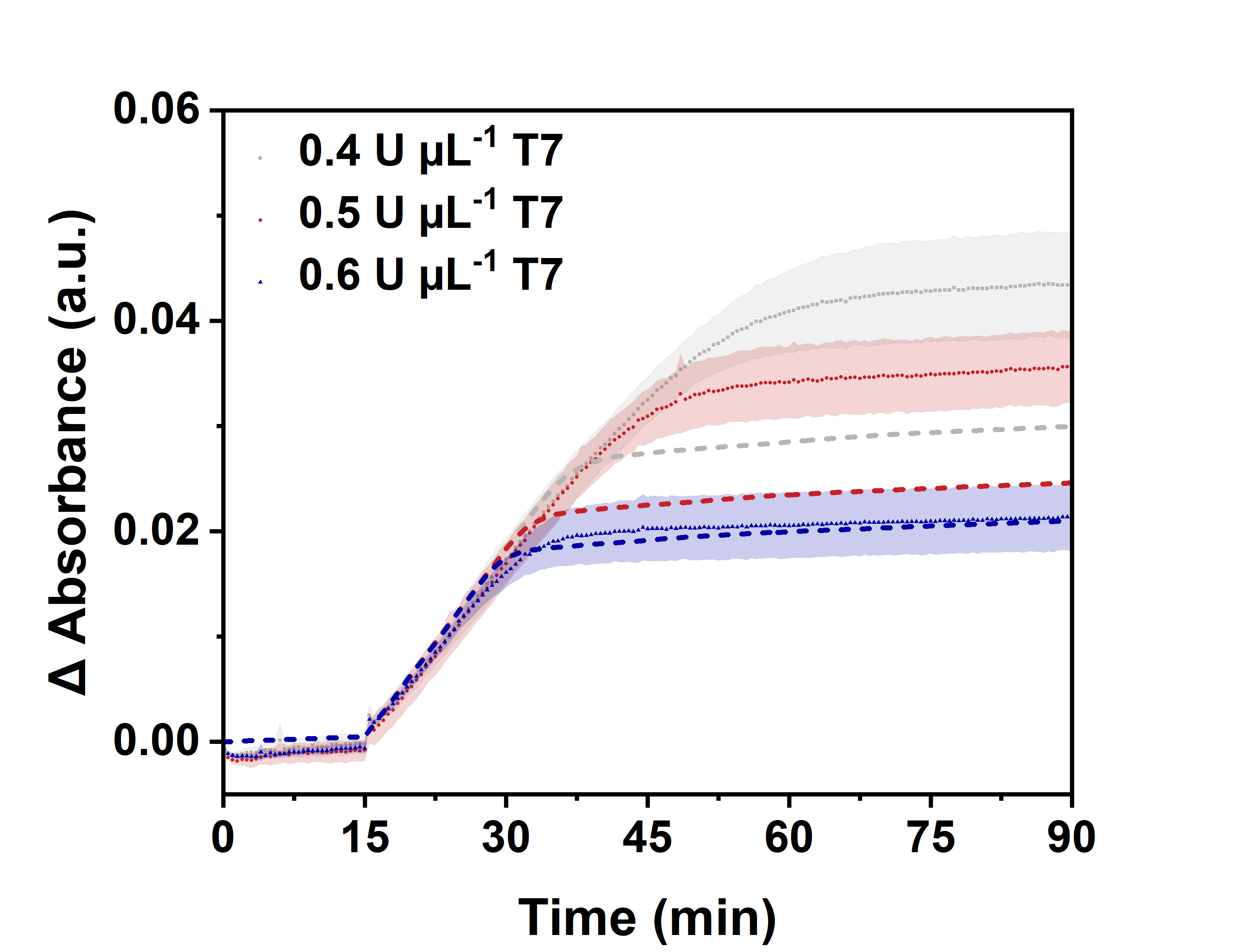


**Figure S39. Dissipative control over trypsin activity in the presence of different T7 concentrations.** Δ absorbance at 405 nm plotted against the time in the presence of 0.4, 0.5, or 0.6 U µL^-1^ T7, L-BAPNA (100 µM) and trypsin fuel (2 eq.). Data are presented as mean ± standard deviation (SD) of three independent experiments (n = 3); error bars represent the SD.


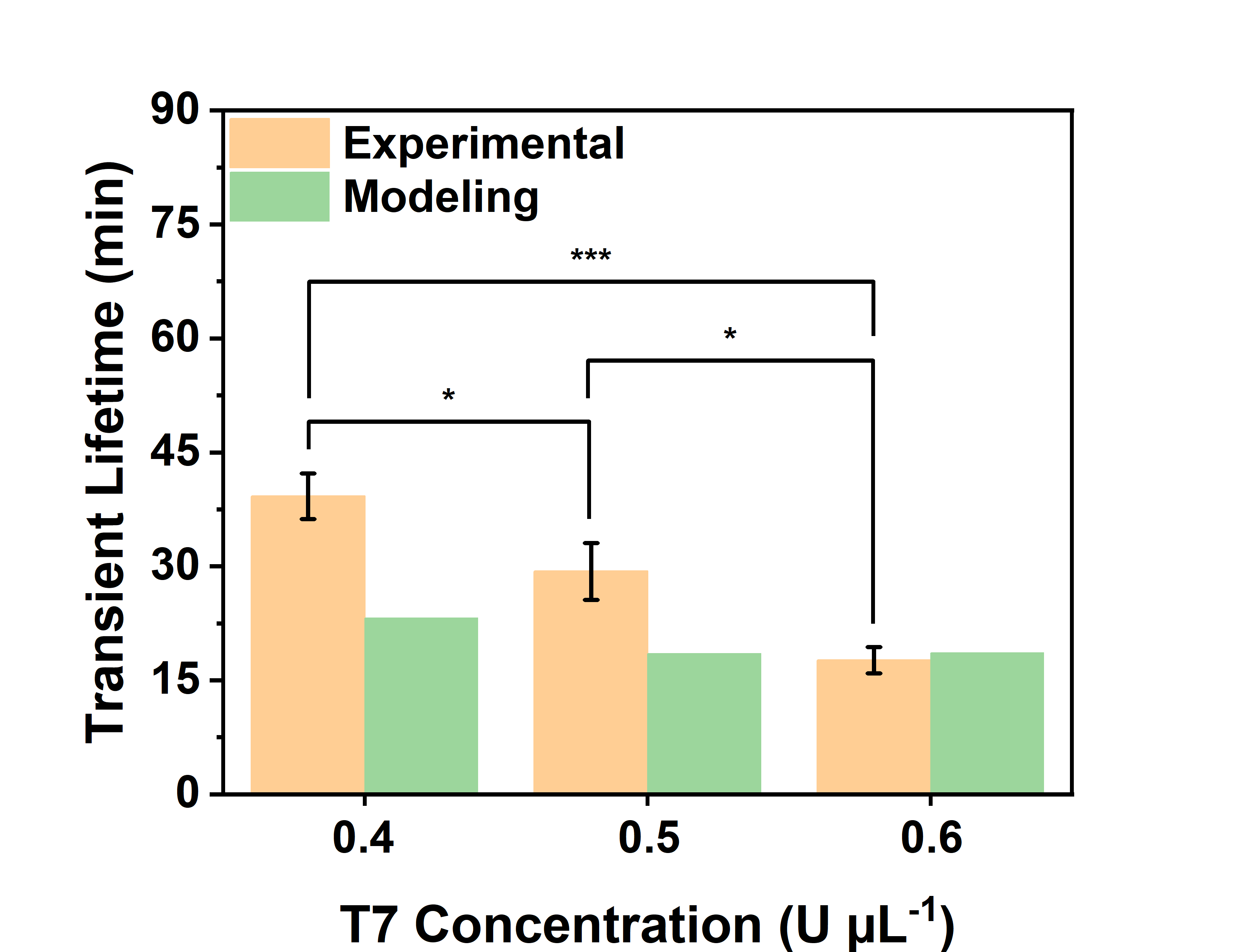


**Figure S40.** Transient lifetimes of trypsin in the presence of different T7 concentrations. Transient lifetime determined experimentally (yellow) and from kinetic modeling (green) in the presence of 0.4, 0.5, and 0.6 U µL^-1^, L-BAPNA (100 µM), and trypsin fuel (2 eq.). Data are presented as mean ± standard deviation (SD) of three independent experiments (n = 3); error bars represent the SD. The statistical significance was determined via analysis of variance (ANOVA) using the Origin 2024b software. Significance: n.s. = not significant; * = p < 0.05; ** = p < 0.01; *** = p < 0.001.


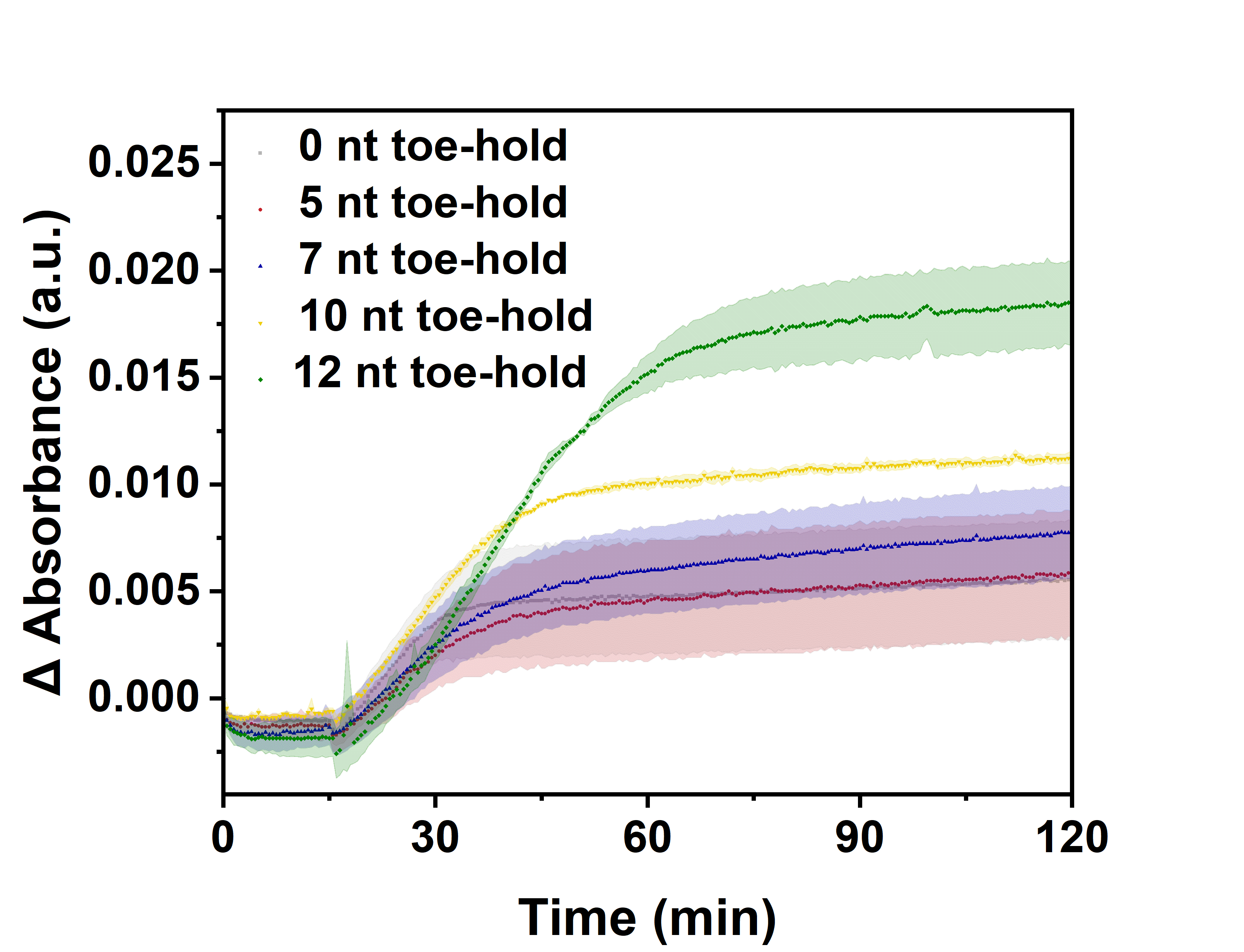


**Figure S41. Dissipative control over trypsin activity in the presence of trypsin fuels with different toe-hold lengths.** Δ absorbance at 405 nm plotted against the time after the addition of trypsin fuels (2 eq.) with toe-holds of 0, 5, 7, 10, or 12 nts in the presence of L-BAPNA (100 µM), and T7 (0.6 U µL^-1^). Data are presented as mean ± standard deviation (SD) of three independent experiments (n = 3); error bars represent the SD.


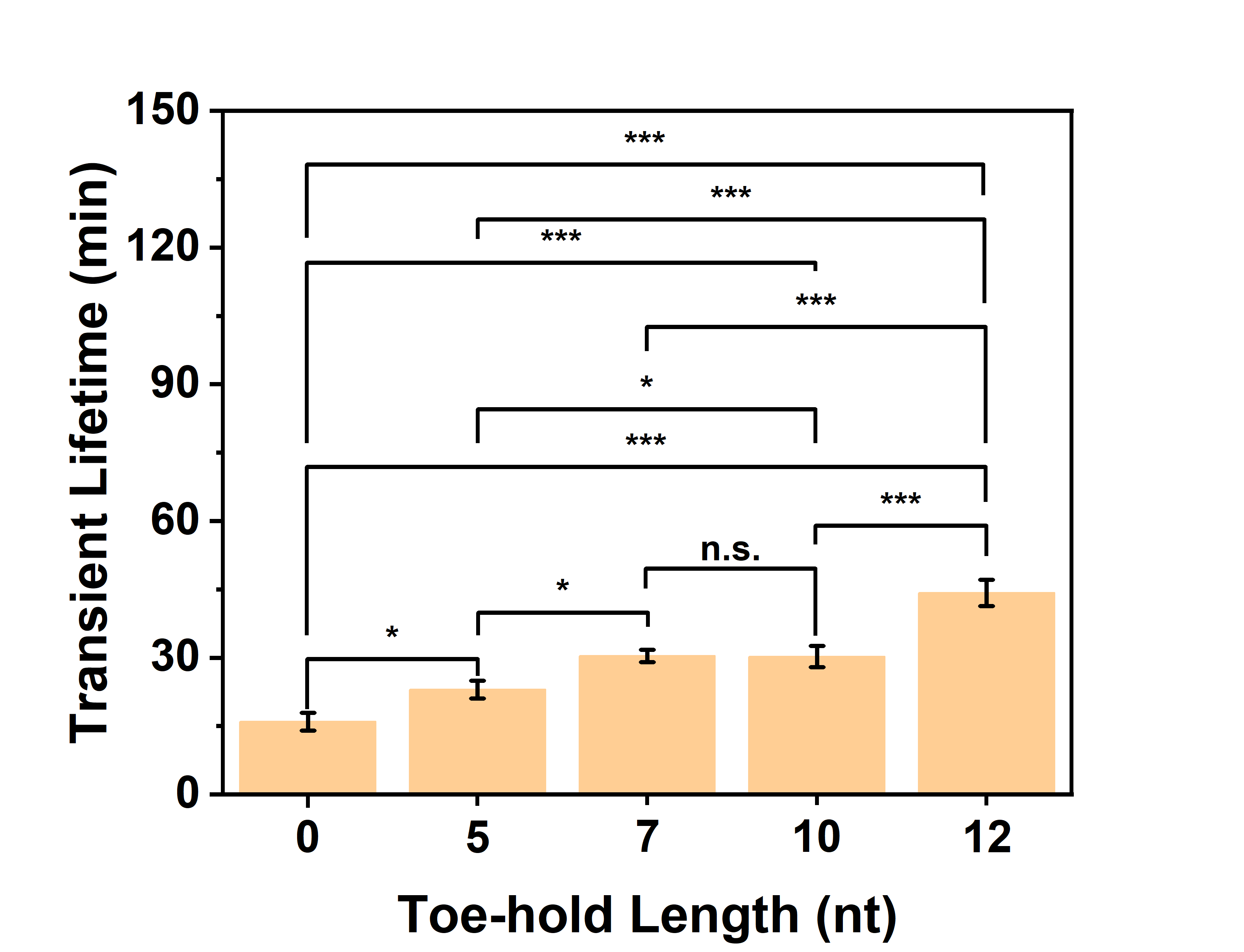


**Figure S42.** Transient lifetimes of trypsin **in the presence of trypsin fuels with different toe-hold lengths**. Transient lifetime determined experimentally (yellow) and from kinetic modeling (green) in the presence of trypsin fuels (2 eq.) containing toe-holds of 0, 5, 7, 10 and 12 nts, L-BAPNA (100 µM) and T7 (0.6 U µL^-1^). Data are presented as mean ± standard deviation (SD) of three independent experiments (n = 3); error bars represent the SD. The statistical significance was determined via analysis of variance (ANOVA) using the Origin 2024b software. Significance: n.s. = not significant; * = p < 0.05; ** = p < 0.01; *** = p < 0.001.


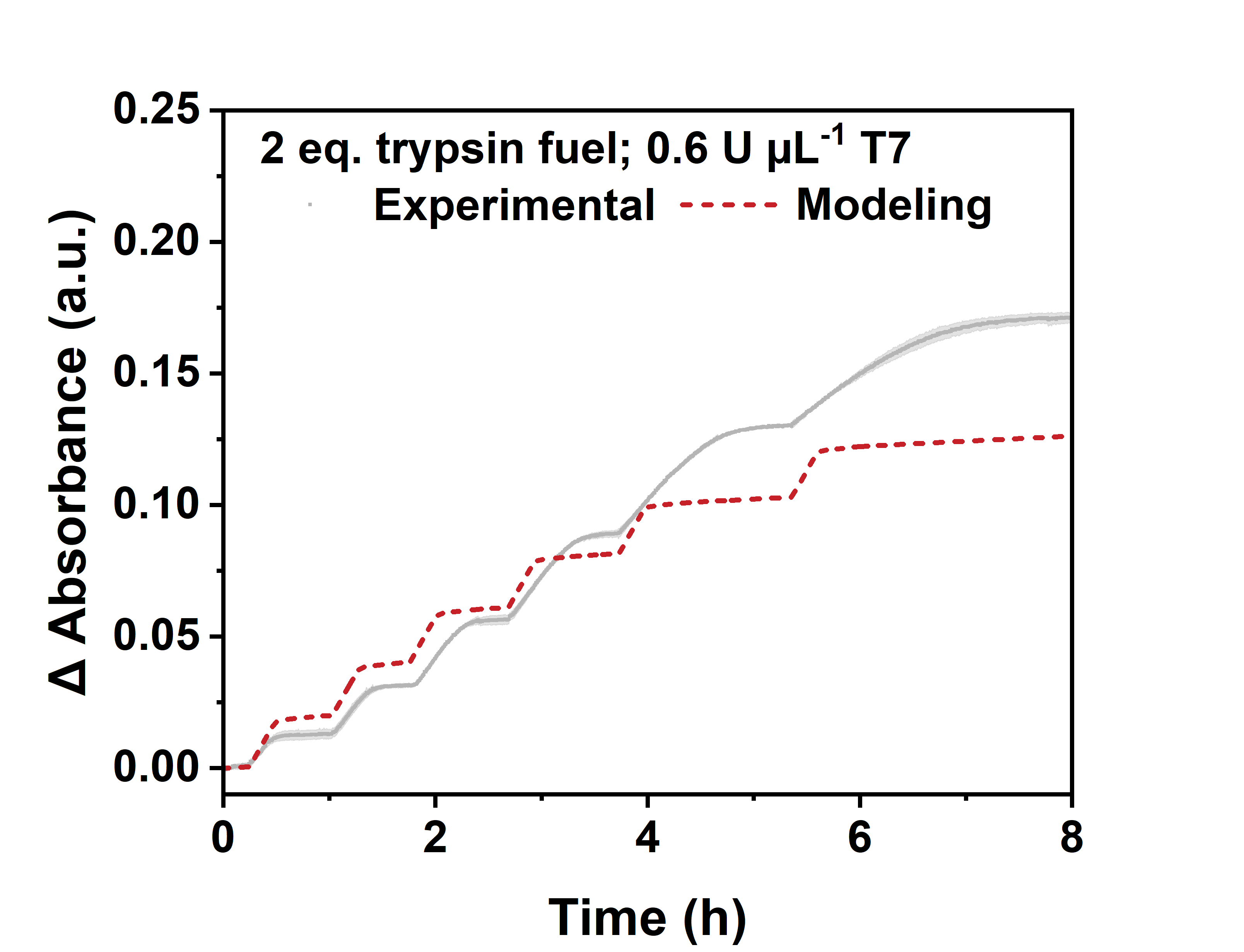


Figure S43. Successive additions of trypsin fuel in the presence of 0.6 U µL^-1^ T7. The normalized absorbance at 405 nm was monitored for 15 min before adding the trypsin fuel (2 eq.). Consecutive trypsin fuel (2 eq.) additions were performed at 15, 62, 105.5, 161.5, 224, and 321.5 min in the presence of T7 (0.6 U µL^-1^). Grey: Δ absorbance at 405 nm reflecting trypsin activity (0.1 µM) activity in the presence of T7 (0.6 U µL^-1^) plotted against the time (experimental data). Red: Δ absorbance at 405 nm reflecting trypsin (0.1 µM) activity in the presence of T7 (0.6 U µL^-1^) plotted against the time (modeled data). Data are presented as mean ± standard deviation (SD) of three independent experiments (n = 3); error bars represent the SD.


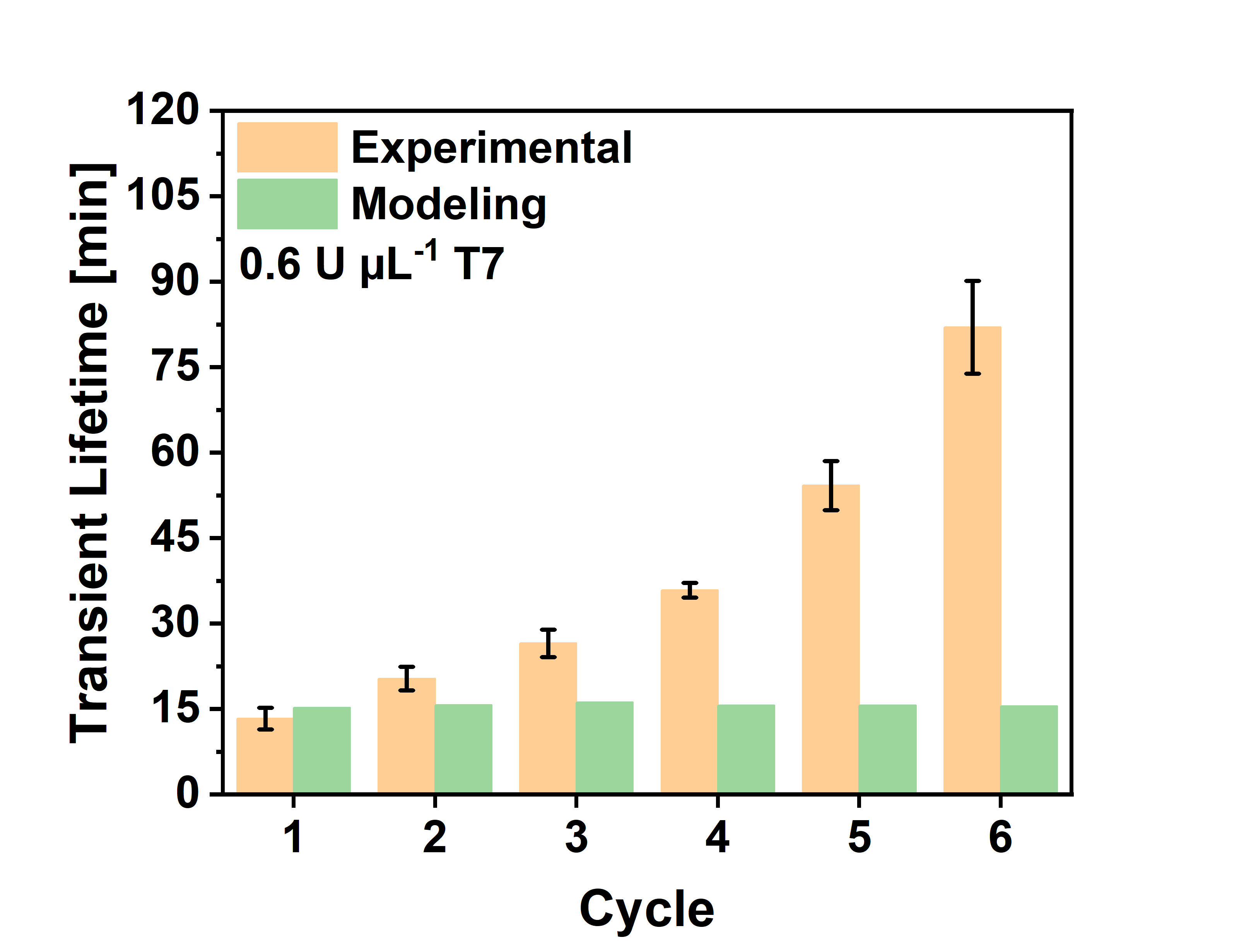


Figure S44. Transient lifetime of trypsin in the presence of 0.6 U µL^-1^ T7 as a function of consecutive fuel additions. Transient lifetimes of trypsin (0.1 µM) in the presence of T7 (0.6 U µL^-1^) were determined from the experimental data (yellow) and the predicted transient lifetimes (green) were determined from the kinetic model. Data are presented as mean ± standard deviation (SD) of three independent experiments (n = 3); error bars represent the SD.

Table S12. Statistical significance determined by analysis of variance (ANOVA) using the Origin 2024b software for the successive additions of trypsin fuel in the presence of 0.6 U µL^-1^ T7.

| Cycle Nr. | 1 | 2 | 3 | 4 | 5 | 6 |
| --- | --- | --- | --- | --- | --- | --- |
| 1 |  | n.s. | n.s. | ** | *** | *** |
| 2 | n.s. |  | n.s. | * | *** | *** |
| 3 | n.s. | n.s. |  | n.s. | *** | *** |
| 4 | ** | * | n.s. |  | ** | *** |
| 5 | *** | *** | *** | ** |  | *** |
| 6 | *** | *** | *** | *** | *** |  |

Significance: n.s. = not significant; * = p < 0.05; ** = p < 0.01; *** = p < 0.001.


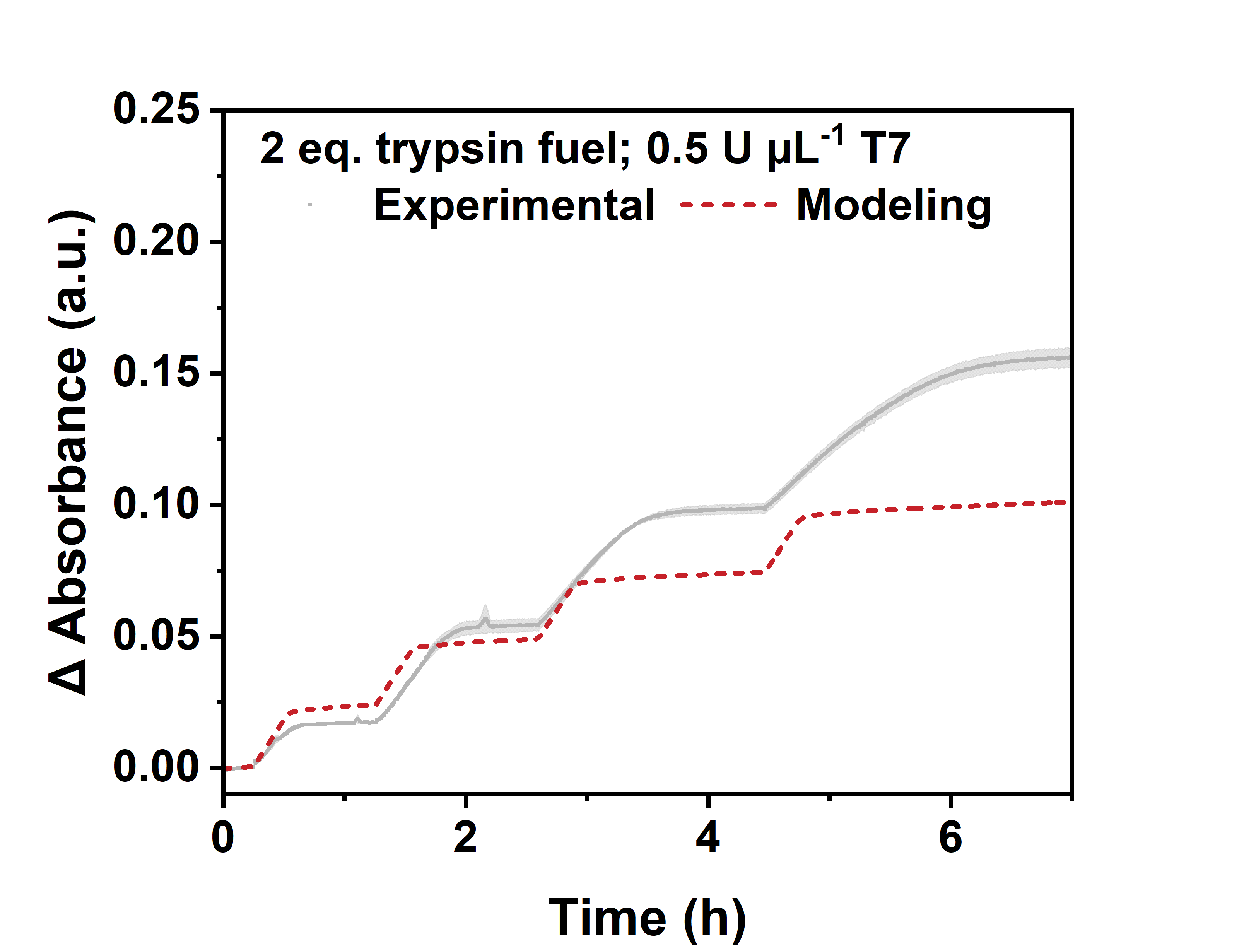


Figure S45. Successive additions of trypsin fuel in the presence of 0.5 U µL^-1^ T7. The normalized absorbance at 405 nm was monitored for 15 min before adding the trypsin fuel (2 eq.). Consecutive trypsin fuel (2 eq.) additions were performed at 15, 75.5, 156, and 268 min in the presence of T7 (0.5 U µL^-1^). Grey: Δ absorbance at 405 nm reflecting trypsin activity (0.1 µM) activity in the presence of T7 (0.5 U µL^-1^) plotted against the time (experimental data). Red: Δ absorbance at 405 nm reflecting trypsin (0.1 µM) activity in the presence of T7 (0.5 U µL^-1^) plotted against the time (modeled data). Data are presented as mean ± standard deviation (SD) of three independent experiments (n = 3); error bars represent the SD.


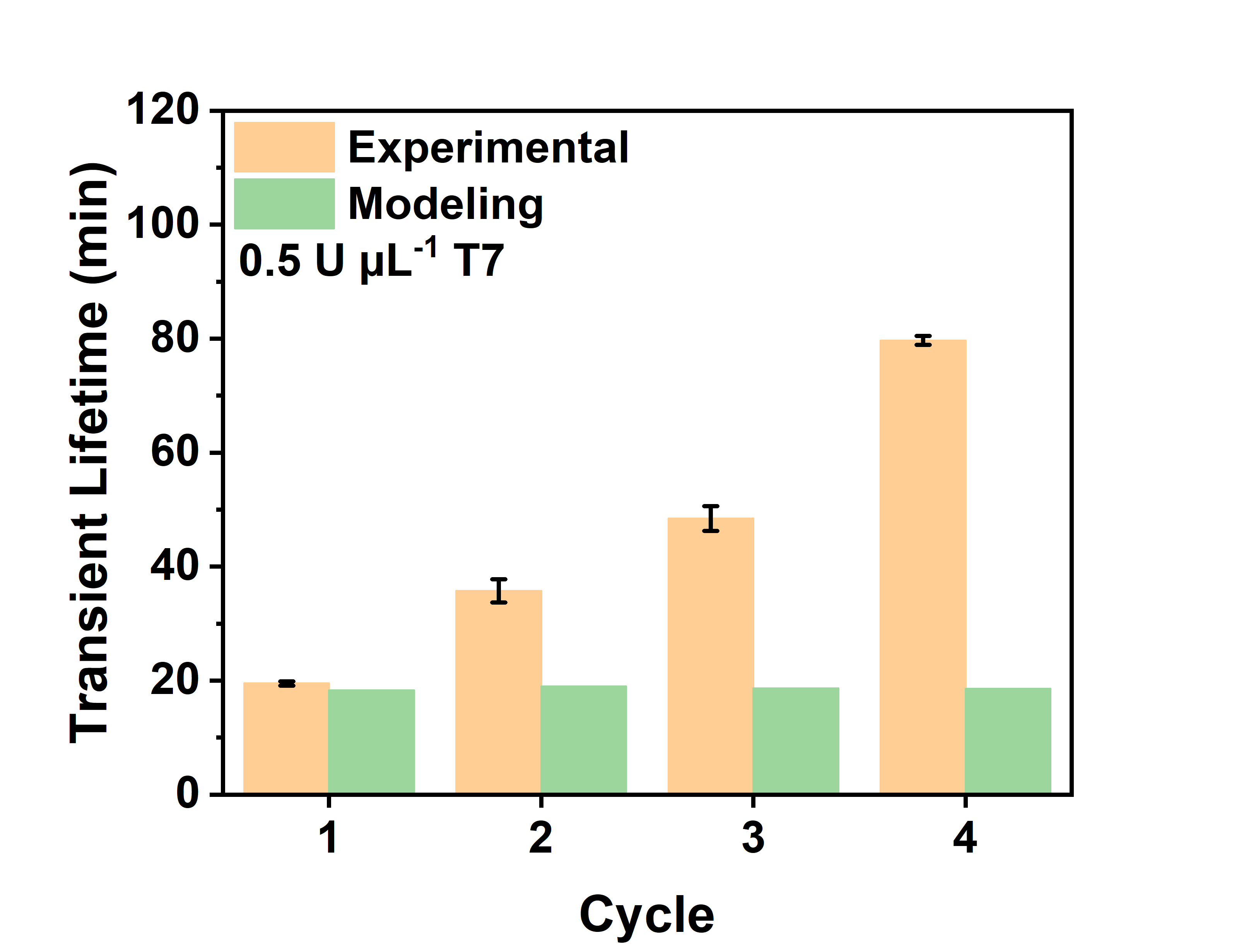


Figure S46. Transient lifetime of trypsin in the presence of 0.5 U µL^-1^ T7 as a function of consecutive fuel additions. Transient lifetimes of trypsin (0.1 µM) in the presence of T7 (0.5 U µL^-1^) were determined from the experimental data (yellow) and the predicted transient lifetimes (green) were determined from the kinetic model. Data are presented as mean ± standard deviation (SD) of three independent experiments (n = 3); error bars represent the SD.

Table S13. Statistical significance determined by analysis of variance (ANOVA) using the Origin 2024b software for the successive additions of trypsin fuel in the presence of 0.5 U µL^-1^ T7.

| Cycle Nr. | 1 | 2 | 3 | 4 |
| --- | --- | --- | --- | --- |
| 1 |  | *** | *** | *** |
| 2 | *** |  | *** | *** |
| 3 | *** | ***. |  | *** |
| 4 | *** | *** | *** |  |

Significance: n.s. = not significant; * = p < 0.05; ** = p < 0.01; *** = p < 0.001.


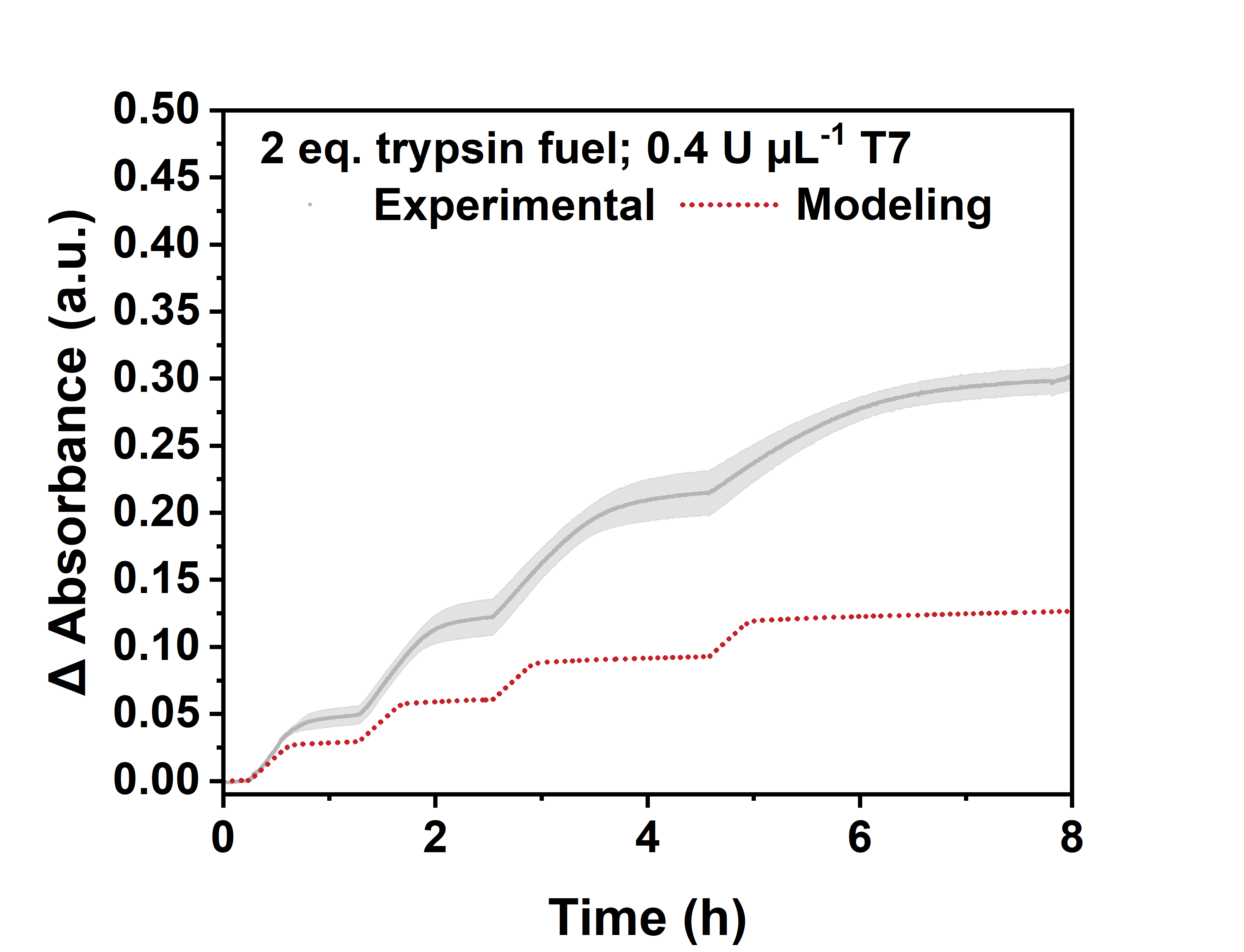


Figure S47. Successive additions of trypsin fuel in the presence of 0.4 U µL^-1^ T7. The normalized absorbance at 405 nm was monitored for 15 min before adding the trypsin fuel (2 eq.). Consecutive trypsin fuel (2 eq.) additions were performed at 15, 77, 152.5, and 275 min in the presence of T7 (0.4 U µL^-1^). Grey: Δ absorbance at 405 nm reflecting trypsin activity (0.1 µM) activity in the presence of T7 (0.4 U µL^-1^) plotted against the time (experimental data). Red: Δ absorbance at 405 nm reflecting trypsin (0.1 µM) activity in the presence of T7 (0.4 U µL^-1^) plotted against the time (modeled data). Data are presented as mean ± standard deviation (SD) of three independent experiments (n = 3); error bars represent the SD.


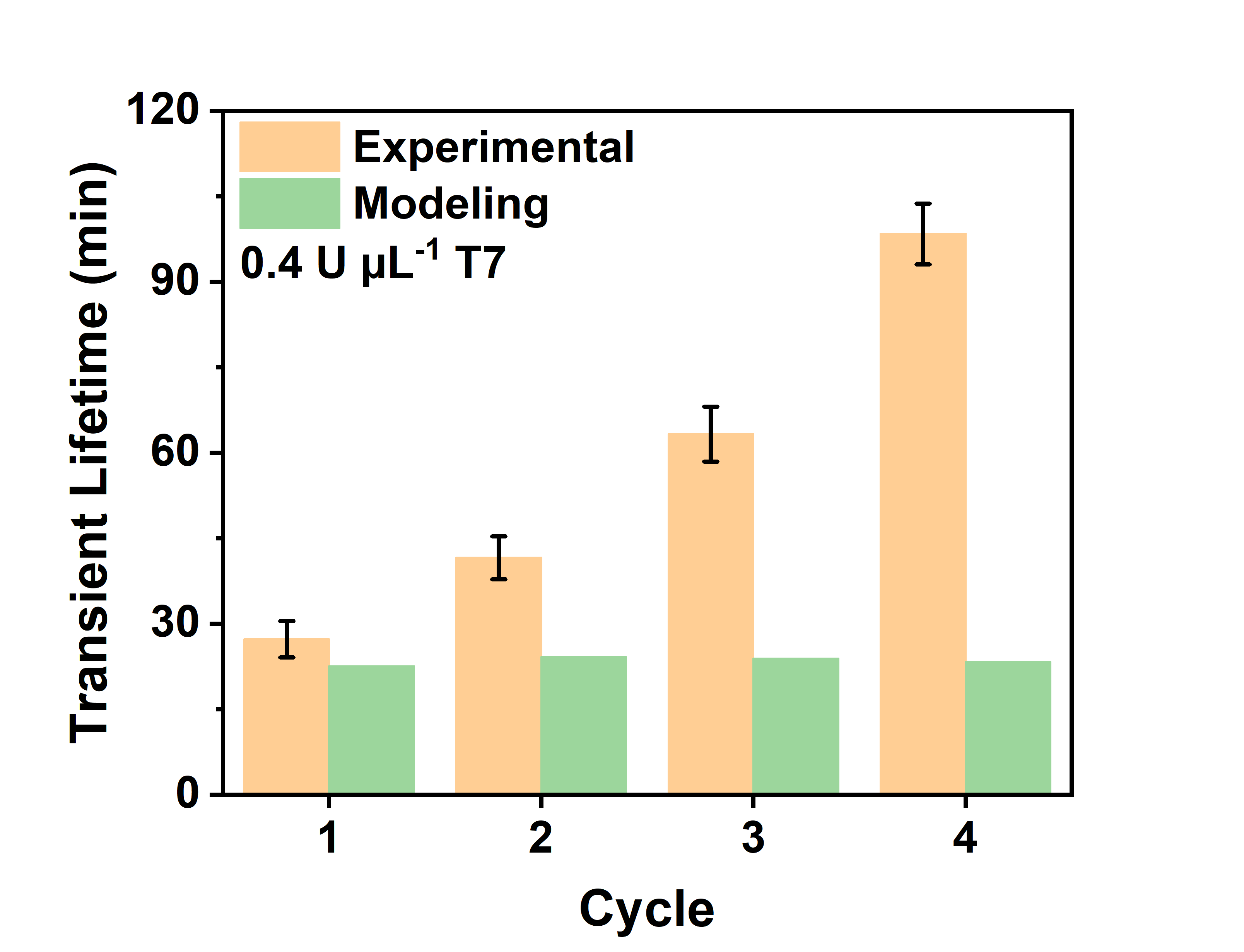


Figure S48. Transient lifetime of trypsin in the presence of 0.4 U µL^-1^ T7 as a function of consecutive fuel additions. Transient lifetimes of trypsin (0.1 µM) in the presence of T7 (0.4 U µL^-1^) were determined from the experimental data (yellow) and the predicted transient lifetimes (green) were determined from the kinetic model. Data are presented as mean ± standard deviation (SD) of three independent experiments (n = 3); error bars represent the SD.

Table S14. Statistical significance determined by analysis of variance (ANOVA) using the Origin 2024b software for the successive additions of trypsin fuel in the presence of 0.4 U µL^-1^ T7.

| Cycle Nr. | 1 | 2 | 3 | 4 |
| --- | --- | --- | --- | --- |
| 1 |  | * | *** | *** |
| 2 | * |  | ** | *** |
| 3 | *** | ** |  | *** |
| 4 | *** | *** | *** |  |

Significance: n.s. = not significant; * = p < 0.05; ** = p < 0.01; *** = p < 0.001

**
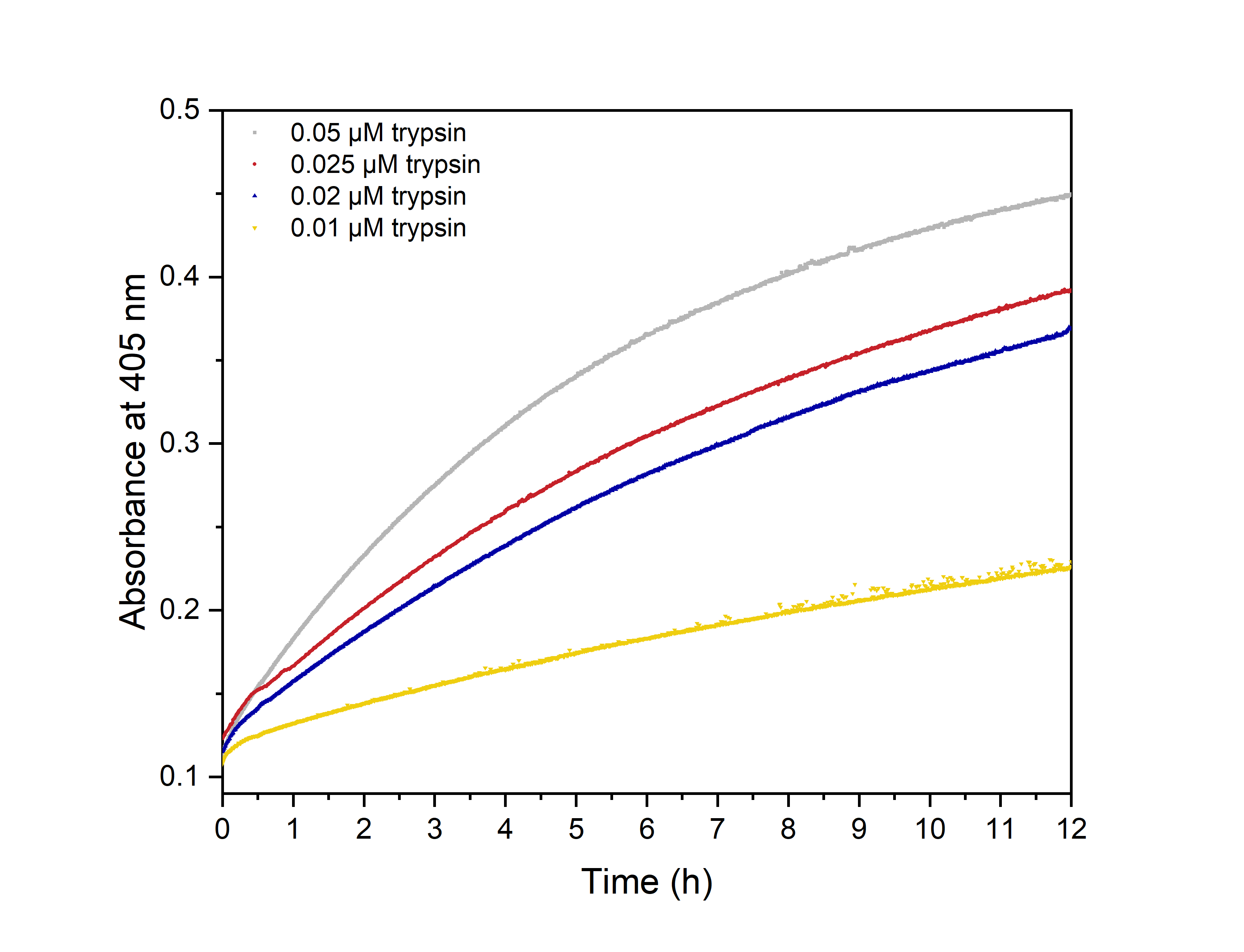
**

Figure S49. Reduction of the trypsin concentration. The absorbance intensity at 405 nm of different trypsin concentrations in the presence of L-BAPNA (100 µM) was monitored for 12 h.


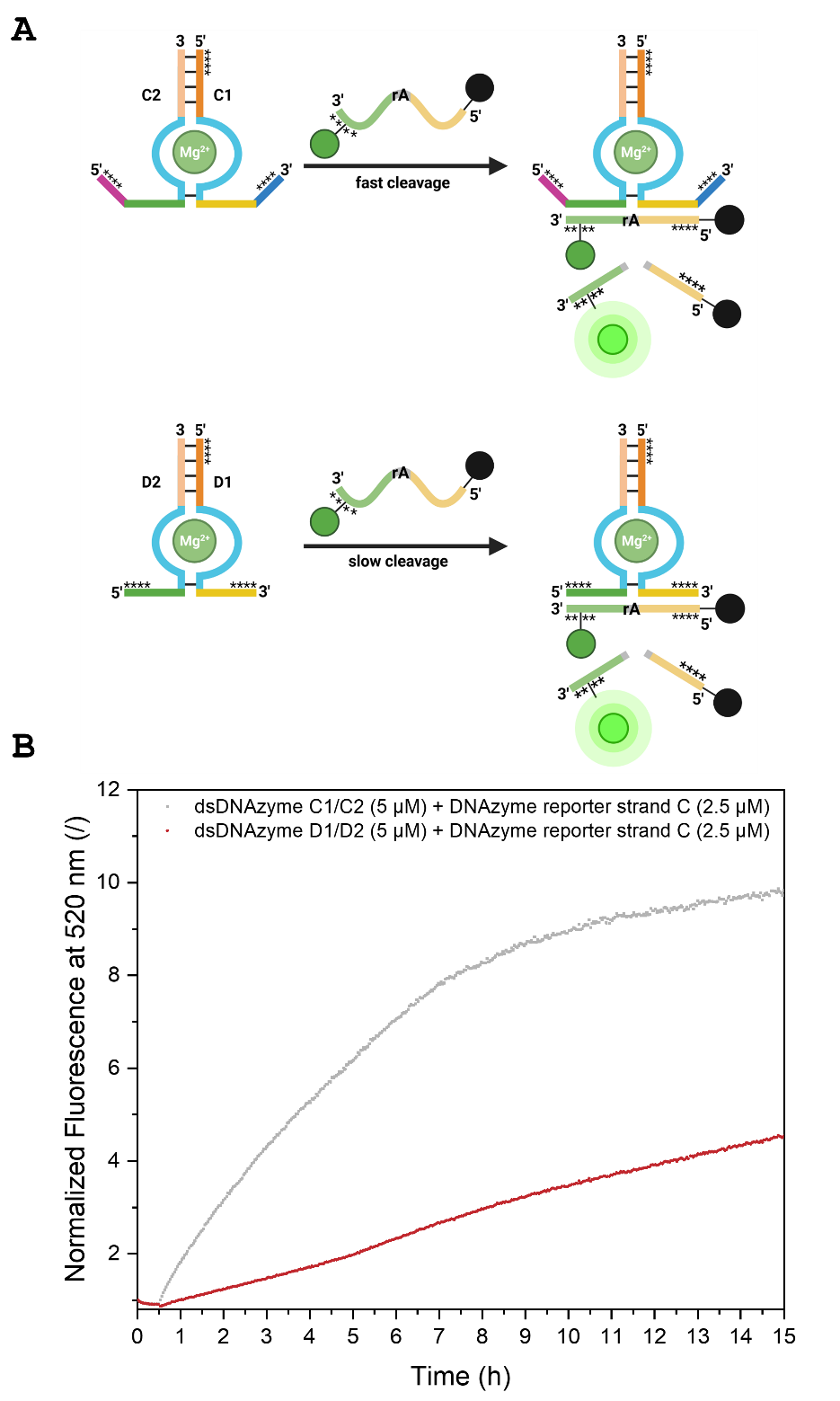
Figure S50. Comparison of the RNA cleavage kinetics of the extended dsDNAzyme C1/C2 and the non-extended dsDNAzyme D1/D2. (A) Schematic representation of the hybridization of the dsDNAzyme C1/C2 or D1/D2 (5 µM) and the DNAzyme reporter strand, followed by the reporter strand cleavage. DsDNAzyme C1/C2 was extended at the 3’-end of DNAzyme C1 and the 5’-end of DNAzyme C2 to avoid PS modification in the recognition site, leading to an increased DNAzyme reporter strand cleavage rate, compared to dsDNAzyme D1/D2. (B) The normalized fluorescence intensity at 520 nm after excitation at 490 nm was monitored for 15 min before adding the ssDNAzyme fuel C2 or D2 (5 µM). Panel A created by Biorender.com

Table S15. Summary of the sample compositions corresponding to the colored traces in Figure S51. Grey: Serves as a positive control demonstrating that trypsin and the DNAzyme can be active simultaneously in the same solution. Red: contains trypsin deactivated by the aptamer in the presence of the dsInhib/trypsin fuel duplex and is used to check for leaching and unwanted trypsin activation. The yellow trace corresponds to the same composition as the red trace but in the presence of T7, allowing DNA digestion–mediated activation of trypsin. The remaining graphs correspond to the same composition in the presence of only T7 (Blue) and both T7 and Exo III (Yellow).

| Color | Trypsin |  | Aptamer | DNAzyme C1 | DNAzyme fuel C2 | DNAzyme reporter strand C | Trypsin fuel | dsInhib/trypsin fuel | BAPNA | T7 | ExoIII |
| --- | --- | --- | --- | --- | --- | --- | --- | --- | --- | --- | --- |
| Gray | ✓ |  | ✓ | ✓ | ✓ | ✓ | ✓ | – | ✓ | – | – |
| Red | ✓ |  | ✓ | – | – | – | – | ✓ | ✓ | – | – |
| Blue | ✓ |  | ✓ | – | – | – | – | ✓ | ✓ | ✓ | – |
| Yellow | ✓ |  | ✓ | – | – | – | – | ✓ | ✓ | ✓ | ✓ |


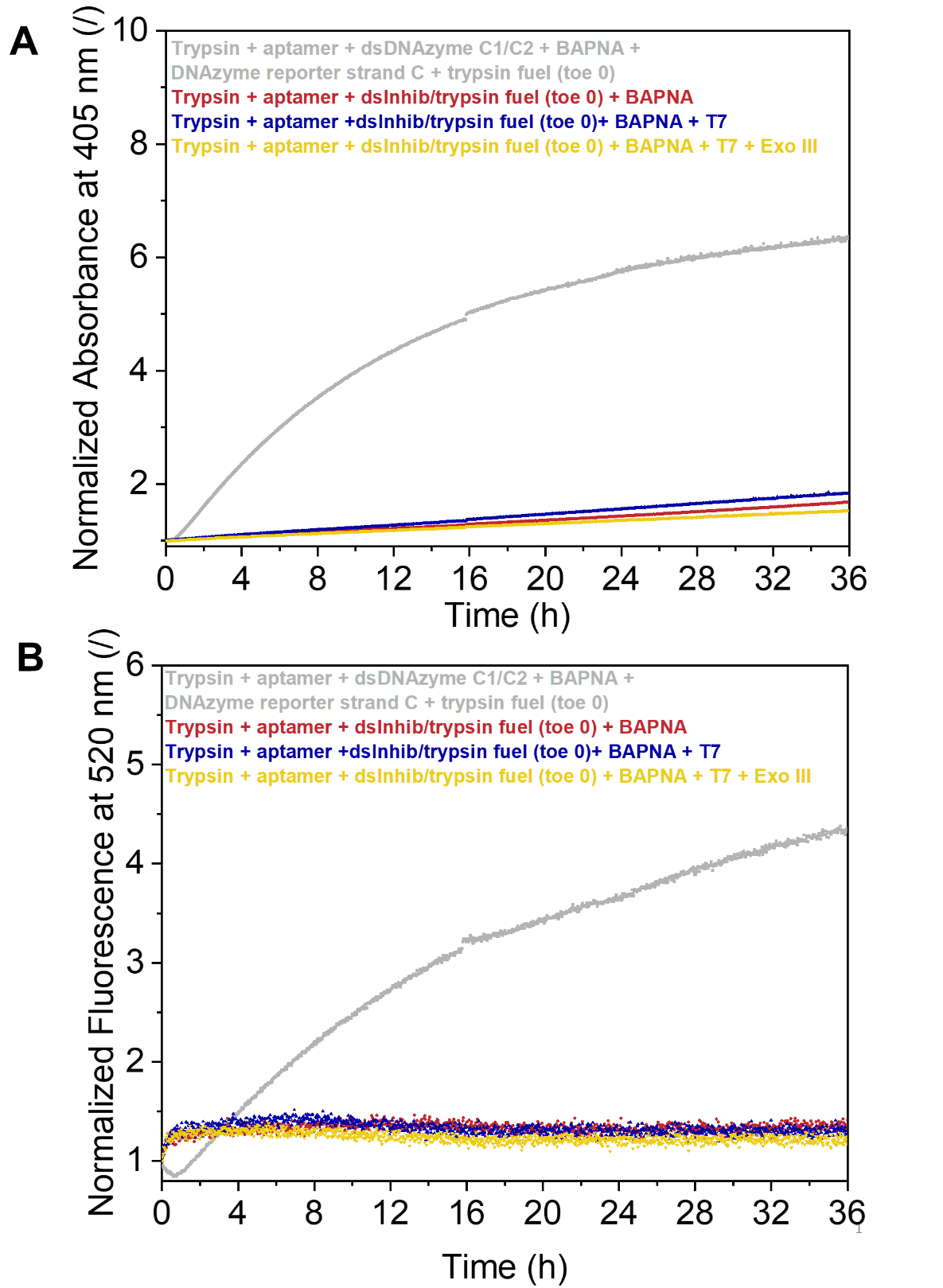
Figure S51. Control experiments are conducted to ensure that no unwanted spontaneous strand displacement between the inhibitor/trypsin fuel duplex and the aptamer occurs in the presence or absence of Exo III and T7. (A) Monitoring of the absorbance intensity at 405 nm over time. (B) Monitoring of the fluorescence intensity at 520 nm after excitation at 490 nm. Gray: Trypsin (0.025 µM) and dsDNAzyme C1/C2 (5 µM) activity in the presence of aptamer (0.25 µM), trypsin fuel (5 µM), L-BAPNA (250 µM), and DNAzyme reporter strand C (1 µM) under non-dissipative conditions (positive control). Red: dsInhib/trypsin fuel (toe0) (5 µM) in the presence of the aptamer (0.25 µM)-deactivated trypsin (0.025 µM), and L-BAPNA (250 µM). Blue: dsInhib/trypsin fuel (toe0) (5 µM) in the presence of the aptamer-deactivated (0.25 µM) trypsin (0.025 µM), T7 (0.6 U µL^-1^), and L-BAPNA (250 µM). Yellow: dsInhib/trypsin fuel (toe0) (5 µM) in the presence of the aptamer-deactivated (0.25 µM) trypsin (0.025 µM), T7 (0.6 U µL^-1^), Exo III (0.025 U µL^-1^), and L-BAPNA (250 µM).

Table S16. Summary of the sample compositions corresponding to the colored traces in Figure S52. Gray: serves as a positive control demonstrating that trypsin and the DNAzyme can be active simultaneously in the same solution. Red: contains deactivated trypsin in the presence of the Inhib/trypsin fuel duplex together with both Exo III and T7, but only the single-stranded DNAzyme C1 (no DNAzyme fuel C2), and is used to test for unspecific activation. Blue: corresponds to the same composition but without Exo III and with the fully assembled dsDNAzyme C1/C2 present. Yellow: represents the complete interlinked system, containing both exonucleases and all components required for coupled DNAzyme/trypsin activation.

| Color | Trypsin |  | Aptamer | DNAzyme C1 | DNAzyme fuel C2 | DNAzyme reporter strand C | Trypsin fuel | dsInhib/trypsin fuel | BAPNA | T7 | ExoIII |
| --- | --- | --- | --- | --- | --- | --- | --- | --- | --- | --- | --- |
| Gray | ✓ |  | ✓ | ✓ | ✓ | ✓ | ✓ | – | ✓ | – | – |
| Red | ✓ |  | ✓ | ✓ | – | ✓ | – | ✓ | ✓ | ✓ | – |
| Blue | ✓ |  | ✓ | ✓ | ✓ | ✓ | – | ✓ | ✓ | ✓ | – |
| Yellow | ✓ |  | ✓ | ✓ | ✓ | ✓ | – | ✓ | ✓ | ✓ | ✓ |


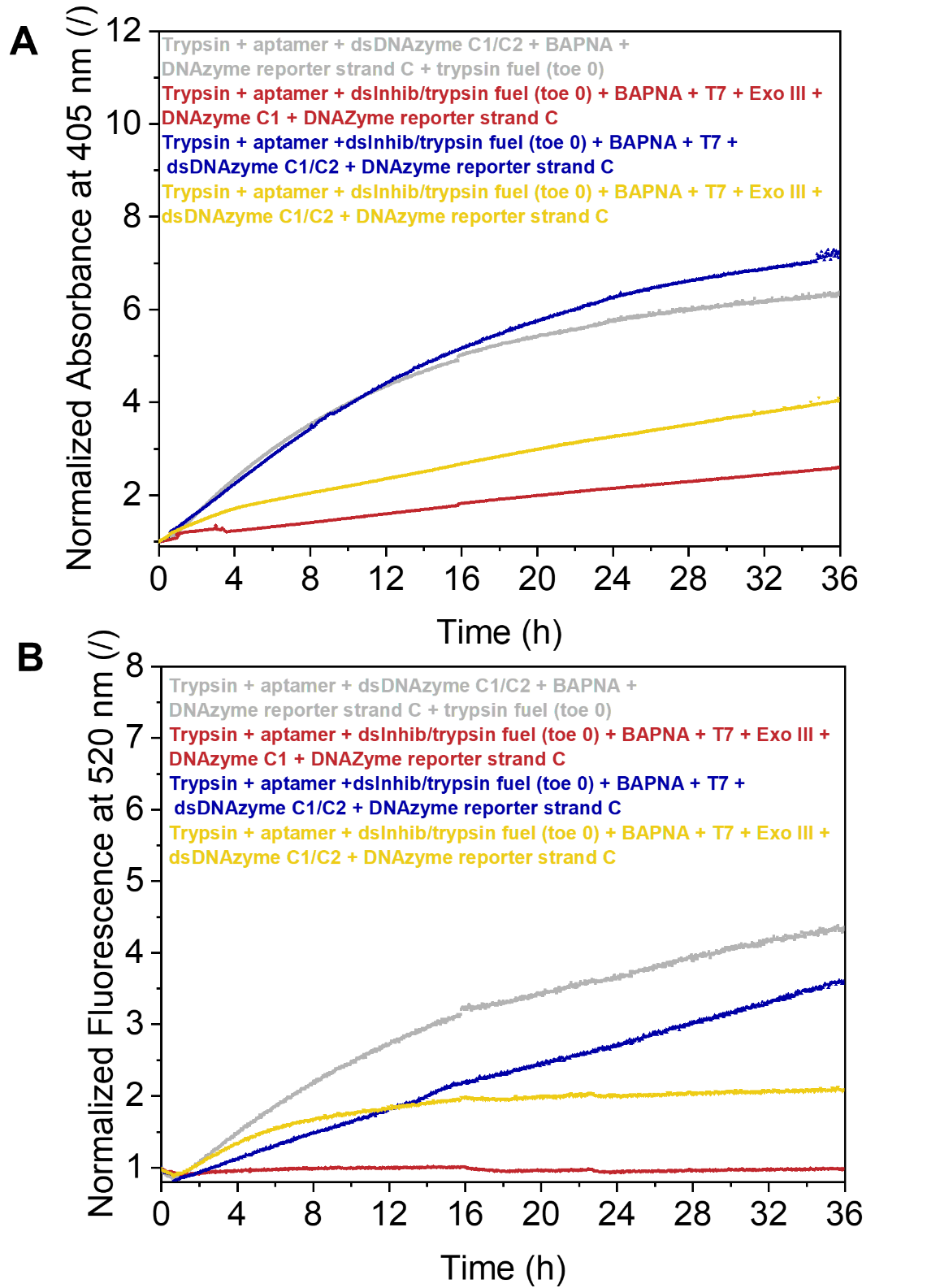
Figure S52. Proof of concept for the dissipative control over a biocatalytic DNA cascade reaction. (A) Monitoring of the absorbance intensity at 405 nm over time. (B) Monitoring of the fluorescence intensity at 520 nm after excitation at 490 nm. Gray: Trypsin (0.025 µM) and dsDNAzyme C1/C2 (5 µM) activity in the presence of aptamer (0.25 µM), trypsin fuel (5 µM), L-BAPNA (250 µM), and DNAzyme reporter strand C (1 µM) under non-dissipative conditions (positive control). Red: Trypsin (0.025 µM) and ssDNAzyme C1 (5 µM) activity in the presence of aptamer (0.25 µM), L-BAPNA (250 µM), DNAzyme reporter strand C (1 µM), T7 (0.6 U µL^-1^), Exo III (0.025 U µL^-1^), and dsInhib/trypsin fuel (toe0) (5 µM). Blue: Trypsin (0.025 µM) and dsDNAzyme C1/C2 (5 µM) activity in the presence of aptamer (0.25 µM), L-BAPNA (250 µM), DNAzyme reporter strand C (1 µM), dsInhib/trypsin fuel (toe0) (5 µM), and T7 (0.6 U µL^-1^). Yellow: Trypsin (0.025 µM) and dsDNAzyme C1/C2 (5 µM) activity in the presence of aptamer (0.25 µM), L-BAPNA (250 µM), DNAzyme reporter strand C (1 µM), dsInhib/trypsin fuel (toe0) (5 µM), T7 (0.6 U µL^-1^), and Exo III (0.025 U µL^-1^).

**References**

[1] Q. Xu, A. Cao, L. Zhang, C. Zhang, ”Rapid and Label-Free Monitoring of Exonuclease III-Assisted Target Recycling Amplification,” *Anal. Chem. 84* **( 2012)**: 10845, <https://doi.org/10.1021/ac303095z>.

[12] P. Virtanen, R. Gommers, T. E. Oliphant, M. Haberland, T. Reddy, D. Cournapeau, E. Burovski, P. Peterson, W. Weckesser, J. Bright et al., ”SciPy 1.0: fundamental algorithms for scientific computing in Python,” *Nat. Methods 17* **(2020)**: 261, https://doi.org/10.1038/s41592-019-0686-2.
